# Structure-activity relationship of BMS906024 derivatives for *Cryptosporidium parvum* growth inhibition

**DOI:** 10.1016/j.bmcl.2023.129328

**Published:** 2023-06-15

**Authors:** Seungheon Lee, Melissa S. Love, Ramkumar Modukuri, Arnab K. Chatterjee, Lauren Huerta, Ann P. Lawson, Case W. McNamara, Jan R. Mead, Lizbeth Hedstrom, Gregory D. Cuny

**Affiliations:** a Department of Pharmacological and Pharmaceutical Sciences, University of Houston, Health Building 2, Houston, TX 77204, USA; b Calibr, a Division of The Scripps Research Institute, La Jolla, CA, 92037, USA; c Department of Biology, Brandeis University, 415 South St., Waltham, MA 02454, USA; d Department of Chemistry, Brandeis University, 415 South St., Waltham, MA 02454, USA; e Atlanta VA Medical Center and Department of Pediatrics, Emory University School of Medicine, Atlanta, GA 30322, USA

## Abstract

BMS906024, a γ-secretase inhibitor that blocks Notch signaling, was previously shown to inhibit *Cryptosporidium parvum* growth *in vitro*. A structure–activity relationship (SAR) analysis of BMS906024 reported herein demonstrates the importance of the stereochemistry of the C-3 benzodiazepine and the succinyl β-substituent. However, concomitant removal of the succinyl α-substituent and switching the primary amide with secondary amides was tolerated. For example, **32** (**SH287**) inhibited *C. parvum* growth in HCT-8 host cells with an EC_50_ = 6.4 nM and an EC_90_ = 16 nM; however, blocking *C. parvum* growth with BMS906024 derivatives was correlative with inhibition of Notch signaling, highlighting that additional SAR analysis will be needed to separate these two activities.

## Graphical abstract

**Figure:**
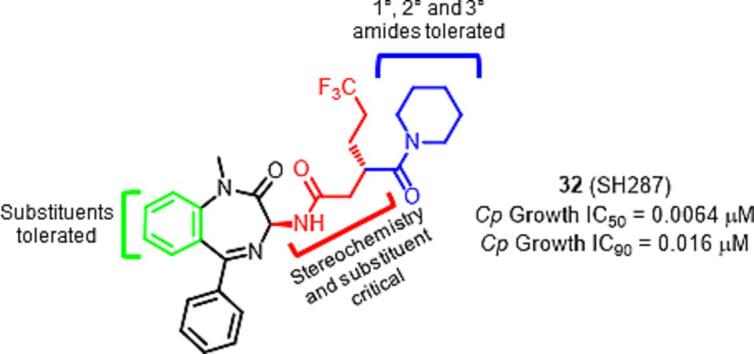

*Cryptosporidium parvum* (*C. parvum*) is an intracellular protozoan parasite that causes cryptosporidiosis primarily in the gastrointestinal (GI) tract of infected hosts, including humans. The common symptoms of infection are watery diarrhea, vomiting, nausea, dehydration, and abdominal cramps.^1^ Cryptosporidiosis can lead to death in immunocompromised individuals and malnourished young children.^2^ Infection is caused by ingestion of sporulated oocysts transmitted through contaminated water or food, or by direct contact with infected animals or humans.^3^ In addition, the oocysts are remarkably resistant to disinfectants, including chlorination, making them difficult to eliminate from the environment that can result in widespread outbreaks.^4^, ^5^

Nitazoxanide (NTZ) is an FDA-approved drug for the treatment of cryptosporidiosis, but it has very limited efficacy for vulnerable populations.^6^, ^7^, ^8^ Unfortunately, clofazimine, an FDA-approved drug for leprosy, failed a recent phase 2a clinical trial for the treatment of cryptosporidiosis in human immunodeficiency virus (HIV)-infected adults, illustrating the need for additional therapeutic options.^9^, ^10^, ^11^, ^12^ Several molecular targets and associated inhibitors have emerged as promising approaches, including phosphatidylinositol 4-kinase (PI(4)K),^13^, ^14^ calcium-dependent protein kinases (CDPKs)^15^, ^16^, ^17^, ^18^, ^19^, ^20^, ^21^, ^22^, ^23^ and *t*-RNA synthetases, although as yet no new therapies have emerged from these efforts.^24^, ^25^, ^26^, ^27^, ^28^, ^29^ The need for alternative strategies continues in order to establish a diverse array of therapeutics for the treatment of cryptosporidiosis.

BMS906024 (**1**, Fig. 1) is a potent orally active γ-secretase inhibitor that blocks Notch cell signaling (e.g. Notch 1 IC_50_ = 2 nM; Notch 3 IC_50_ = 3 nM).^30^ Notch cell signaling, which is a downstream pathway of γ-secretase, has emerged as a potential therapeutic target for leukemia and solid tumors.^30^ In addition, γ-secretase has been one of the most explored human proteases for potentially treating Alzheimer’s disease.^31^, ^32^, ^33^, ^34^, ^35^ Interestingly, **1** was recently discovered to also potently inhibit *C. parvum* growth (EC_50_ = 1.8 nM) through a ReFRAME comprehensive library study by Janes, *et al*.^36^ Intrigued by this later finding, we initiated a structure–activity relationship (SAR) analysis of **1** for *C. parvum* growth inhibition anti-*Cryptosporidium* activity from Notch inhibition, the results of which are reported herein.

**Fig. 1:**
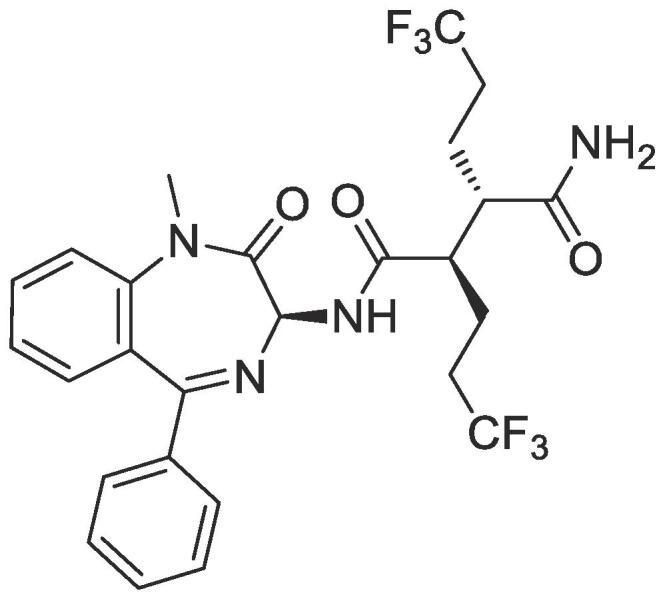
Structure of BMS906024 (**1**).

Three regions of **1** were the focus of the SAR study for *C. parvum* growth inhibition. This included the terminal primary amide, the alkyl substituents on the succinyl group and several positions on the benzodiazepine fragment. The general synthetic approach employed coupled enantiomerically enriched 3-amino benzodiazepine fragments to various succinyl moieties. Therefore, many of the enantiomerically enriched benzodiazepine intermediates were prepared as outlined in Scheme 1. Initially, 2-aminobenzophenones were cyclized using a glycine derivative to give **2**. Amide alkylation gave **3**, followed by installation of an azide to produce **4**. Reduction of the azide generated amine **5** that was coupled to Boc-phenylalanine to give **6**. This material was then deprotected and crystalized as the HCl salt to produce amine **7**. Finally, removal of the phenylalanine gave the 3-amino benzodiazepine intermediates **8** and **9**.^37^.

**Scheme 1:**
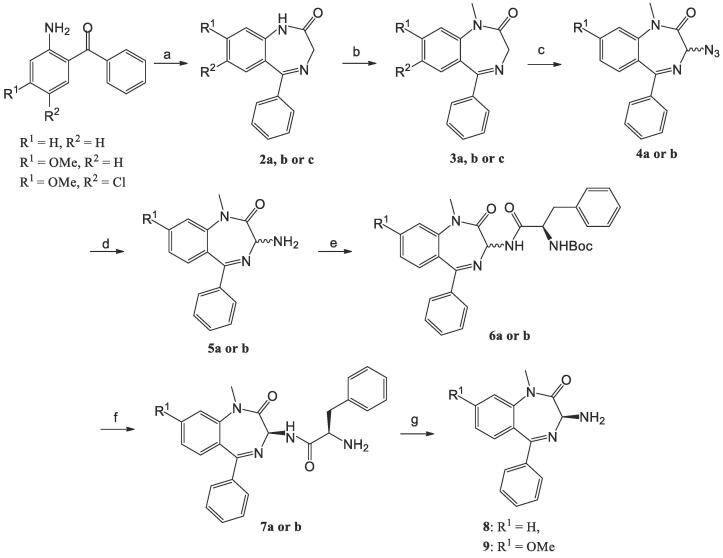
Synthesis of benzodiazepine fragments. (a) i) Boc-Gly-OH, EEDQ, DCM, rt, 16 h. ii) TFA, DCM, rt, 2.5 h. iii) CH_3_COONH_4_, AcOH, rt, 24 h, 25–71%. Or i) bromoacetyl chloride, TEA (or Na_2_CO_3_), DCM (or ACN), 0 °C - rt, 58–82%. ii) 7 N NH_3_ in MeOH, rt, 16 h, 35–86%. (b) NaH, MeI, DMF (or THF), rt, 6.5 h, 34–94%. (c) 0.5 M KHMDS, 2,4,6-triisopropylbenzenesulfonyl azide, AcOH, THF, rt, 2.5 h, 80–85%. (d) PPh_3_, THF/H_2_O, rt, 24 h, 81–98%. (e) Boc-d-Phe, EDC∙HCl, HOBt hydrate, TEA, DMF (or DCM), rt, 16 h, 60–85%. (f) i) HCl_(g)_, EtOAc, 0 °C, 1 h. ii) crystalized from EtOH, 24–35%. (g) i) phenyl isothiocyanate, DCM, 30 ∼ 40 °C, 1.5 h. ii) TFA, 50 °C, 1 h, 49–84%.

Derivatives with a 7-chloro substituent on the benzodiazepine were prepared from 4-chloro-3-methoxyaniline following a published method^38^, ^39^ and then incorporated into the procedure outlined above to give **3c**. De-alkylation of the ether using BBr_3_ produced phenol **10**, which was coupled with 3-bromooxetane to yield **11** (Scheme 2). Finally, installation of an amine in the 3-position was accomplished as previously described generating **13**.

**Scheme 2:**
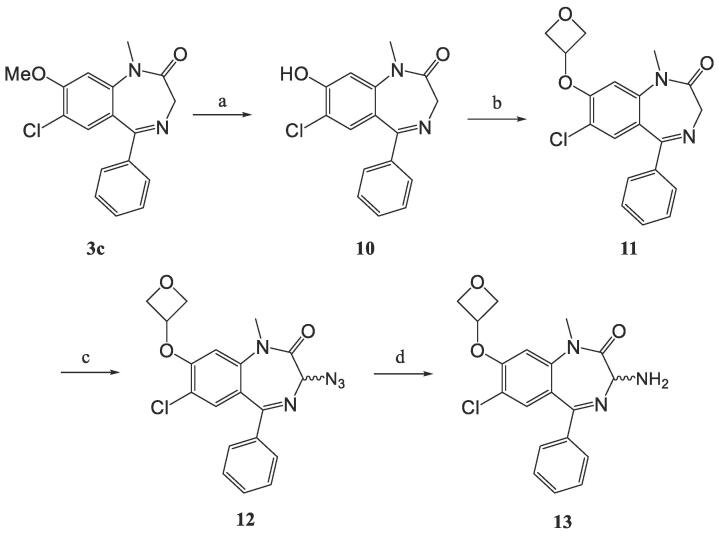
The additional substituents on the benzodiazepine. (a) 1 M BBr_3_, DCM, 0 °C ∼ rt, 16 h, 75%. (b) 3-bromooxetane, K_2_CO_3_, DMF, 100 °C, 16 h, 73%. (c) 0.5 M KHMDS, 2,4,6-triisopropylbenzenesulfonyl azide, AcOH, THF, rt − 30 °C, 2.5 h, 84%. (d) PPh_3_, THF/H_2_O, rt, 24 h, 83%.

The succinyl carboxylic acid derivatives were synthesized utilizing chiral auxiliaries. For example, 5,5,5-trifluoropentanoic acid was converted to **14**, which was then stereoselectively alkylated to give **15** (Scheme 3). The chiral auxiliary was removed (e.g. **16**), followed by primary amide formation (e.g. **20a**) and then removal of the *t*-Bu ester to provide **21a**. Alternatively, the *t*-Bu ester was removed, followed by secondary or tertiary amide formation and then removal of the chiral auxiliary to give **23a-e**. The same procedures were used to generate **21b** and **25**, except that the (*R*)-enantiomer of the chiral auxiliary was used.

**Scheme 3:**
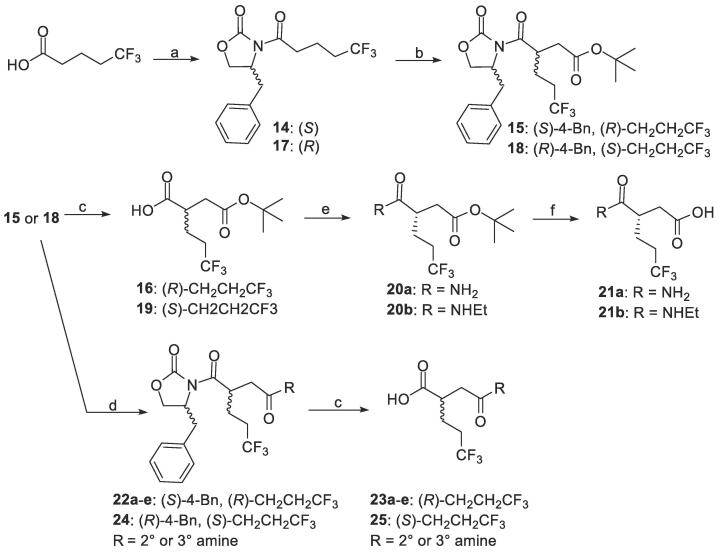
Synthesis of intermediates for succinyl carboxylic acid derivatives. (a) i) oxalyl chloride, DMF (cat.), DCM, rt, 2 h. ii) (*S*)-4-benzyloxazolidin-2-one, THF, −78 °C, then 2.5 M *n*-BuLi in hexane, −78 °C, 1.5 h, 60–94%. (b) *t*-butyl bromoacetate, 1 M LiHMDS in THF (or 1 M NaHMDS), THF, −78 °C, 9 h, 33–65% (c) 30% H_2_O_2_, LiOH, THF/H_2_O, rt, 1.5 h. (d) i) TFA, DCM, rt, 3.5 h. ii) disuccinimidyl carbonate, TEA, THF, rt, 3.5 h. iii) amines (or cyclic amines), THF, rt, 5 h, 55–75% (e) amines (or ammonium chloride), EDC∙HCl, HOBt hydrate, TEA, DMF, rt, 16 h, 32–85%. (f) TFA, DCM, rt, 3.5 h.

Succinyl carboxylic acid derivatives containing *gem*-dimethyl substituents were also synthesized. Dimethylsuccinic anhydride was allowed to react with *n*-propylamine in THF at room temperature to produce the separable amides **26a** and **26b** in a 3.5:1 ratio (30% yield), respectively, with the structure assignment being confirmed by HMBC analysis (Scheme 4).

**Scheme 4:**
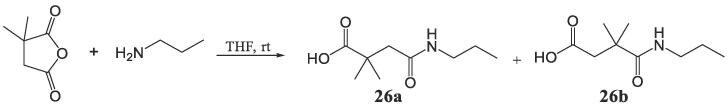
Synthesis of *gem*-dimethyl carboxylic acid intermediates.

Next, the benzodiazepine fragments were coupled with the succinyl carboxylic acid intermediates using TBTU or EDC∙HCl/HOBt hydrate mediated amide coupling (Scheme 5). In the case of the oxetane derivative **13**, coupled with a succinic carboxylic acid derivative followed by preparative HPLC gave diastereomers **46a** and **46b**.

**Scheme 5:**
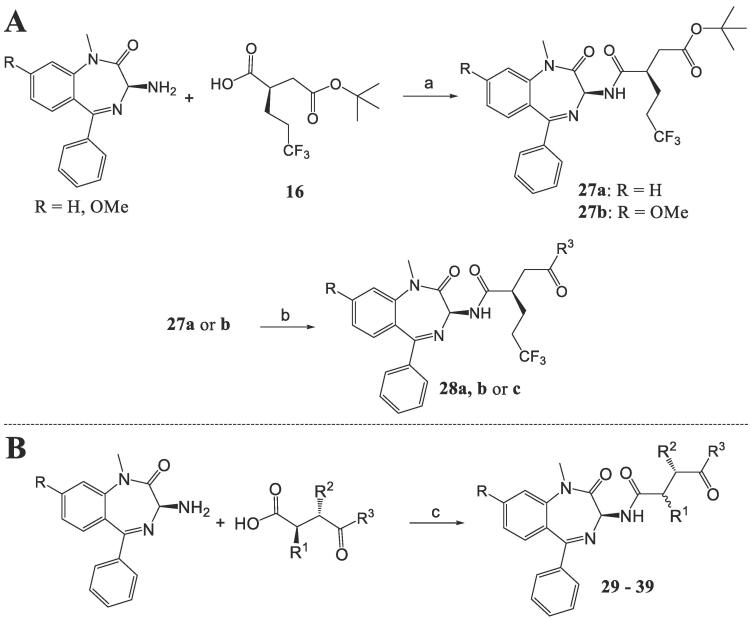
Synthesis of **28**–**39**. (a) TBTU (or EDC∙HCl, HOBt hydrate), TEA, DMF, rt, 16 h, 63–81%. (b) i) TFA, DCM, rt, 3.5 h. ii) disuccinimidyl carbonate, TEA, THF, rt, 3.5 h. iii) amine, THF, rt, 6 h, 38–80%. (c) EDC∙HCl, HOBt hydrate, TEA, DCM, rt, 16 h, 36–92%.

Finally, several other ether derivatives were synthesized by following the route outlined in Scheme 6. Compounds **28c** and **34** were de-methylated to give phenols **40** and **41**, respectively. Then alkylation with various halogenated substrates under basic conditions generated **42** – **44**. Alternatively, **41** was coupled with phenyl boronic acid to give **45**. However, these reactions were accompanied by epimerization at C3. Subsequently, the pure diastereomers were separated by preparative HPLC.

**Scheme 6:**
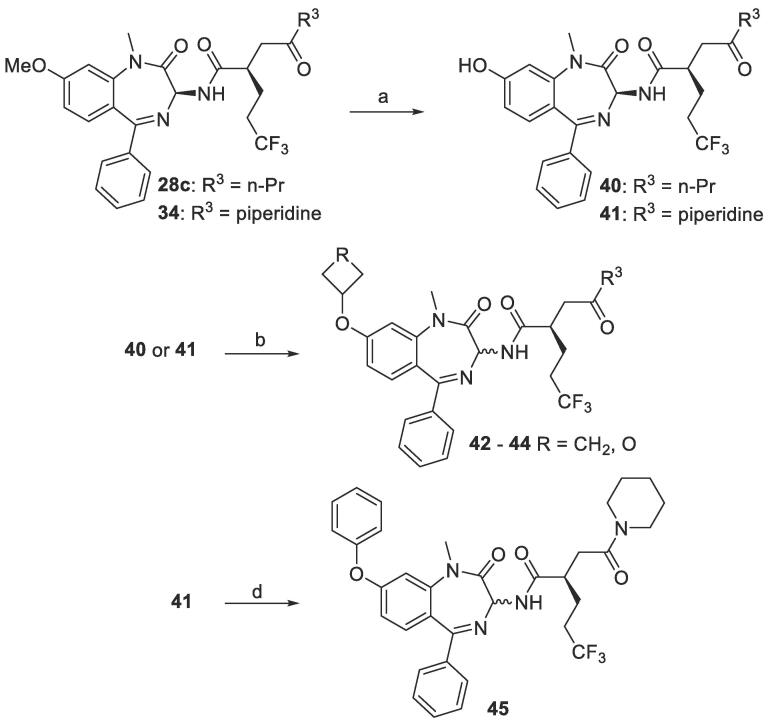
General scheme for switching ether group on benzodiazepine ring. (a) 1 M BBr_3_ (in DCM), 0 °C - rt, 16 h. (b) 3-bromooxetane or bromocyclobutane, K_2_CO_3_, DMF, 50–100 °C, 16 h, 53–75%. (c) EDC∙HCl, HOBt hydrate, TEA, DCM, rt, 16 h, 91%. (d) phenylboronic acid, Cu(OAc)_2_, pyridine, DCM, rt − 40 °C, 16 h, 57%.

The compounds were assessed for *C. parvum* growth inhibition in HCT-8 cells using a reported method.^12^ Briefly, HCT-8 host cells were plated and grown for 24 h. Then test compounds were added at various concentrations. Two hours later *C. parvum* oocysts (Iowa strain) isolated from infected calves were added. After 48 h incubation, cells were fixed, stained with fluorescent markers, and imaged with a CX5 imager (Cellomics, Thermo). The images were processed by HCS Studio Scan software, and raw Selected Object Count (HCT-8 cells) and Spot Count (*C. parvum*) values were analyzed in Genedata Screener (v13.0-Standard) to determine *C. parvum* growth compared to untreated controls. Three independent determinations of EC_50_ and EC_90_ values were performed and averaged.

Modifications of the succinyl and amide portions of BMS906024 were initially explored (Table 1). Deletion of either trifluoromethyl propyl group while retaining the primary amide (e.g. **36** and **CIS580**) eliminated *C. parvum* growth inhibition. However, removing the substituent on the α-C of the succinyl group and replacing the primary amide with a secondary propyl amide (**28a**) retained potent *C. parvum* growth inhibition (EC_50_ = 19 nM). Truncating the alkyl amide (**28b**) or replacing with *n*-alkyl tertiary amides (**29** and **30**) or a *t*-butyl ester (**27a**) resulted in moderate reduction of inhibitor activities. Whereas inversion of the stereochemistry of the alkyl group on the β-C of the succinyl group (**35**), retaining only the alkyl group on the α-C (**37**), incorporation of *gem*-dimethyl groups at the α-C (**38b**) or β-C (**38a**), or removal of both alkyl groups (**39**) eliminated *C. parvum* growth inhibition. Finally, three cyclic tertiary amides were also examined with the piperidine derivative (**32**) displaying the most potent *C. parvum* growth inhibitory activity (EC_50_ = 6.4 nM).

**Table 1:** SAR analysis of succinyl and amide portions of BMS906024 for *C. parvum* growth inhibition.

**Compound**	**R^1^**	**R^2^**	***R*^3^**	***C. parvum***	
				**EC_50_ (µM)**	**EC_90_ (µM)**
**BMS906024 (1)**	(*R*)-CH_2_CH_2_CF_3_	(*S*)-CH_2_CH_2_CF_3_	NH_2_	0.0018	0.0023
**36**	H	(*S*)-CH_2_CH_2_CF_3_	NH_2_	25	>25
**CIS580**	(*R*)-CH_2_CH_2_CF_3_	H	NH_2_	>25	>25
**28a**	(*R*)-CH_2_CH_2_CF_3_	H	NH-*n-*Pr	0.018 (±0.017)	0.056 (±0.0014)
**28b**	(*R*)-CH_2_CH_2_CF_3_	H	NHEt	0.28 (±0.21)	0.61 (±0.23)
**29**	(*R*)-CH_2_CH_2_CF_3_	H	N(*n-*Pr)_2_	1.45 (±1.26)	2.79 (±1.73)
**30**	(*R*)-CH_2_CH_2_CF_3_	H	N(Et)_2_	0.11 (±0.0077)	0.31 (±0.091)
**27a**	(*R*)-CH_2_CH_2_CF_3_	H	O-*t*-Bu	0.55 (±0.26)	1.12 (±0.22)
**35**	(*S*)-CH_2_CH_2_CF_3_	H	NH-*n-*Pr	>25	>25
**37**	H	(*S*)-CH_2_CH_2_CF_3_	NH-*n-*Pr	>25	>25
**38b**	H	(Me)_2_	NH-*n-*Pr	>25	>25
**38a**	(Me)_2_	H	NH-*n-*Pr	>25	>25
**39**	H	H	NH-*n-*Pr	>25	>25
**31**	(*R*)-CH_2_CH_2_CF_3_	H		0.11 (±0.062)	0.23 (±0.12)
**32**	(*R*)-CH_2_CH_2_CF_3_	H		0.0064 (±0.0022)	0.016 (±0.0088)
**33**	(*R*)-CH_2_CH_2_CF_3_	H		0.052 (±0.019)	0.10 (±0.029)

Prior work on the BMS906024 scaffold hinted that substituent on the C-8 position of the benzodiazepine portion might provide modulation of γ-secretase and Notch cell signaling activity.^40^ Therefore, several ethers with an array of steric bulk were examined (Table 2). The methoxy substituent (**28c** and **34**) was found to retain potent *C. parvum* growth inhibition. The cyclobutane and oxetane derivatives (**44a** and **43a**) were active, albeit less than **32**. As previously mentioned, epimerization of C-3 occurred during installation of the ether. However, the configuration was assigned based on the Notch inhibitory activity based on a prior study.^41^ Again, the tertiary cyclic amides were more active than the corresponding secondary amides. A phenyl ether (**45a**) was also tolerated and provided potent growth inhibition (EC_50_ = 25 nM). Installation of a chlorine at C-7 as a means of providing some conformation restriction of the ether was examined. Although this change was tolerated (**46a**), inhibitory activity decreased about 10-fold. In all three cases, the (*S*)-configuration at the 3-position of the benzodiazepine was more active than the corresponding (*R*)-configuration.

**Table 2:** Addition of substituents to the benzodiazepine portion of BMS906024 for *C. parvum* growth inhibition.

**Compound**	**R**	**C3**	**R^1^**	**R^2^**	***C. parvum***	
					**EC_50_ (µM)**	**EC_90_ (µM)**
**28c**	Me	*S*	NH-*n*-Pr	H	0.081 (±0.016)	0.15 (±0.057)
**34**	Me	*S*		H	0.031 (±0.016)	0.066 (±0.029)
**44a**	*c*-Bu	*S*^a^		H	0.077 (±0.018)	0.16 (±0.063)
**44b**	*c*-Bu	*R*^a^		H	2.52 (±1.33)	11.1 (±8.67)
**43a**	oxetane	*S*^a^		H	0.11 (±0.034)	0.21 (±0.10)
**43b**	oxetane	*R*^a^		H	2.29 (±0.089)	4.90 (±0.91)
**42a**	oxetane	*S*^a^	NH-*n*Pr	H	0.35 (±0.10)	0.97 (±0.79)
**42b**	oxetane	*R*^a^	NH-*n*Pr	H	>25	>25
**45a**	phenyl	*S*^a^		H	0.025 (±0.0028)	0.055 (±0.011)
**45b**	phenyl	*R*^a^		H	1.69 (±0.79)	3.61 (±0.42)
**46a**	oxetane	*S*^a^		Cl	0.32 (±0.014)	0.96 (±0.077)
**46b**	oxetane	*R*^a^		Cl	5.47 (±1.52)	12.8 (±2.12)

a The configuration has been assigned based on *C. parvum* inhibition activity.

Next, inhibition of *C. parvum* growth and Notch signaling were compared. Notch signaling was measured using a kit from BPS Bioscience.^40^ In brief, HEK293T/17 cells were transfected to express an extracellular domain-deleted variant of Notch1 (NotchΔ1E) in the presence of either a Notch-responsive firefly luciferase reporter vector (CSL (CBF1/RBP-JK)) or a nonresponsive control. Cells were treated with compounds 24 h post transfection and incubated for an additional 24 h before luciferase activity was measured. Normalized luciferase activities were calculated relative to DMSO (control: 100%). The inhibitory activities of BMS906024 derivatives for *C. parvum* growth and Notch signaling were found to be correlative (Table S1 and Fig. S1).

Given the correlation observed for BMS906024 derivatives, three other γ-secretase inhibitors or modulators (e.g. semagacestat, DAPT and MRK-560) were assessed for *C. parvum* growth inhibition. In all cases, they were significantly less active (EC_50_ = 4.2, 12.5 and 2.5 μM, respectively). These observations suggest that anticryptosporidial activity does not derive from inhibition of γ-secretase and instead may result from engagement of a parasite homolog. To further test this hypothesis, sporozoites were isolated from excysted oocysts by filtration as described.^42^ Sporozoites were then pre-treated with **30** for 45 min, followed by washings to remove excess drug, and then inoculated onto monolayers of HCT-8 cells. After 48 h, parasitic growth was assessed by microscopic examination and quantitated as described.^43^ *C. parvum* growth was inhibited in this experiment, albeit less efficiently (EC_50_ ∼ 0.50 µM) compared to having the compound in the presence of the host cells for an extended time. Nonetheless, these results are consistent with the compound engaging a parasite target.

In conclusion, an SAR analysis of BMS906024 demonstrated that retention of stereochemistry of the C-3 benzodiazepine and succinyl β-substituent were necessary for *C. parvum* growth inhibition. However, concomitant removal of the succinyl α-substituent and switching the primary amide with secondary amides was tolerated. Furthermore, addition of substituents to the 7- or 7,8-positions of the benzodiazepine was permitted. Finally, blocking *C. parvum* growth with BMS906024 derivatives was correlative with Notch signaling inhibition, indicating that further SAR analysis is needed to separate these two activities.

## Declaration of Competing Interest

The authors declare that they have no known competing financial interests or personal relationships that could have appeared to influence the work reported in this paper.

## Data Availability

Data will be made available on request.
