# Supplementary material for: Structure-activity relationship of BMS906024 derivatives for Cryptosporidium parvum growth inhibition
Source: Bioorg Med Chem Lett. 2023 Jun 15;90:129328. doi: 10.1016/j.bmcl.2023.129328 (PMC10290938; doi:10.1016/j.bmcl.2023.129328)
Supplement: Supplementary data 1 [file mmc1.pdf]

Structure-activity relationship of BMS906024 derivatives for *Cryptosporidium parvum* growth inhibition

Seungheon Lee[a], Melissa S. Love[b], Ramkumar Modukuri[b], Arnab K. Chatterjee[b], Lauren Huerta[b], Ann P. Lawson[c], Case W. McNamara[b], Jan R. Mead[e], Lizbeth Hedstrom[c,d], Gregory D. Cuny[a,*]

[a]*Department of Pharmacological and Pharmaceutical Sciences, University of Houston, Health Building 2, Houston, TX, 77204, USA*

[b]*Calibr, a division of The Scripps Research Institute, La Jolla, CA, 92037, USA*

[c]*Department of Biology, Brandeis University, 415 South St., Waltham, MA 02454, USA*

[d]*Department of Chemistry, Brandeis University, 415 South St., Waltham, MA 02454, USA*

[e]*Atlanta VA Medical Center and Department of Pediatrics, Emory University School of Medicine, Atlanta, Georgia, 30322, USA*

Table of Contents

General experimental conditions…………………………………………………………...…………..S2

Synthesis experimental procedures……………………………………………………………………..S4

Biological assay procedures…………………………………………………………………………...S66

Supporting information references…………………………………………………………..…..….S71

General experimental conditions

All reactions involving air-sensitive reagents were carried out in oven-dried glassware equipped with a magnetic stir bar and fitted with rubber septa under argon unless otherwise stated. All commercially available chemicals, reagent grade solvents, and anhydrous solvents were used directly without further purification unless otherwise specified. All reactions were monitored by thin-layer chromatography (TLC) on Baker-flex® silica gel plates (IB2-F) using UV-light (254 and 365 nm) detection or visualizing agents (ninhydrin or phosphomolybdic acid stain). Flash column chromatography was conducted on silica gel (230−400 mesh) using Teledyne Isco CombiFlash® Rf. NMR spectra were recorded at room temperature using a JEOL ECZ-600 (^{1}H NMR at 600 MHz and ^{13}C NMR at 150 MHz) with tetramethylsilane (TMS) as an internal standard. Chemical shifts (δ) are given in parts per million (ppm) with reference to solvent signals [^{1}H-NMR: $CDCl_3$ (7.26 ppm), CD_3OD (3.31 ppm), DMSO-d_6 (2.50 ppm); ^{13}C-NMR: $CDCl_3$ (77.0 ppm), CD_3OD (49.15 ppm), DMSO-d_6 (39.51 ppm)]. Signal patterns are reported as s (singlet), d (doublet), t (triplet), q (quartet), m (multiplet), and br (broad). Coupling constants (*J*) are given in Hz. In the case of diastereomers, NMR data is reported as major [minor] if the corresponding peaks were separated from each other or as a range for the major and minor diastereomers if the peaks were overlapping. By using Thomas Hoover Uni-Melt, melting points were measured and are uncorrected.

High-resolution mass spectra (HRMS) were measured by using Agilent 6530 Q-TOF instrument by Mass spectrometry facility at the Department of Chemistry, the University of Texas at Austin. Electrospray ionization (ESI) was used as an ionization source and the spectra were reported as *m/z* (relative intensity) for the molecular [M] or $[M + H]^+$ ion species. Additional HRMS were also obtained at the University of Massachusetts Mass Spectrometry Center.

All test compounds reported had a purity ≥95% as determined by high-performance liquid chromatography (HPLC) analyses using a Waters 1525 instrument equipped with a quaternary pump and a Proteo-C12 column (250 mm × 1 mm, 4 μm). UV absorption was monitored at λ = 220 nm. HPLC gradient went from 5% to 90% CH_3CN in H_2O (both solvents contain 0.1% trifluoroacetic acid) with a total run time of 30 min and a flow rate of 0.5 mL/min.

Method A. Waters 1525 instrument equipped with a quaternary pump and a Proteo-C12 column (250 mm × 1 mm, 4 μm). UV absorption was monitored at λ = 220 nm. HPLC gradient went from 5% to 90% CH_3CN in H_2O (both solvents contain 0.1% trifluoroacetic acid) with a total run time of 30 min and a flow rate of 0.5 mL/min.

Method B. Waters 1525 instrument equipped with Waters 2489 UV/Visible detector. Kinetex 5u C18 100A column (250 × 4.6 mm) used for analytical and Kinetex 5u C18 100A, AXIA (250 × 21.2 mm) used for preparative purification. HPLC gradient went from 2% to 98% CH_3CN in H_2O (both solvents contain 0.1% trifluoroacetic acid) with a total run time of 30 min and a flow rate of 1 mL/min for analytical analysis and 10 mL/min for preparative purification.

Synthesis experimental procedures

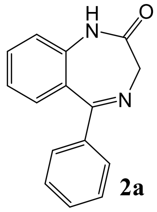

2a

5-Phenyl-1H-benzo[e][1,4]diazepin-2(3H)-one

2-Aminobenzophenone (100 mg, 0.51 mmol), EEDQ (125.4 mg, 0.51 mmol), and Boc-Gly-OH (89 mg, 0.51 mmol) were combined in DCM (3 mL) and stirred overnight at room temperature. The reaction mixture was washed with HCl (10% solution, 2 mL) and then saturated Na_2CO_3 (10 mL), dried over anhydrous Na_2SO_4, filtered and concentrated. The crude material was dissolved in TFA (1 mL) and DCM (2 mL) and stirred at room temperature for 2.5 h. The organic layer was concentrated several times with DCM (3 x 5 mL). To the resulting crude oil, NH_4OAc (192 mg) and AcOH (2 mL) were added, the mixture was stirred at room temperature for 24 h and then concentrated. Water (10 mL) was added and the mixture was extracted with EtOAc (2 × 20 mL). The combined organic extracts were washed with brine, dried over anhydrous Na_2SO_4, filtered, and concentrated. Diethyl ether was added and a precipitation (**2a**) was collected (40 mg, 33%) as a light yellow solid. **^{1}H-NMR** ($CDCl_3$, 600 MHz) δ 9.09 (s, 1H), 7.55-7.49 (m, 3H), 7.46-7.43 (m, 1H), 7.38 (t, J = 7.7 Hz, 2H), 7.33 (d, J = 7.6 Hz, 1H), 7.16 (dd, J = 13.9, 7.7 Hz, 2H), 4.34 (s, 2H); **^{13}C-NMR** ($CDCl_3$, 150 MHz) δ 172.0, 171.0, 139.4, 138.6, 131.7, 131.4, 130.3, 129.6, 128.2, 127.2, 123.3, 121.0, 56.6.

Note, the reaction was repeated with yields of 25–71%.

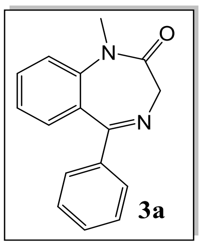

1-Methyl-5-phenyl-1H-benzo[e][1,4]diazepin-2(3H)-one

To solution of **2a** (36 mg, 0.15 mmol) in DMF (3 mL, THF was used for scale-up), NaH (5.4 mg, 0.23 mmol) was added and the reaction was maintained at room temperature for 1.5 h. MeI (14 µL, 0.23 mmol) was added and the resulting mixture was stirred at room temperature for overnight. Additional NaH (< 2 equiv) was added and stirred for 5 h. After completion of the reaction, the mixture was diluted with EtOAc (10 mL) and washed with water (10 mL). The aqueous layer was washed with EtOAc (2 × 5 mL), and the combined organic extracts were washed with brine, dried over anhydrous Na_2SO_4, filtered, and concentrated. The residue was purified by column chromatography on silica gel (EtOAc/hexane, 20:80 to 40:60) to afford **3a** (27 mg, 70%) as a yellow solid. **^{1}H-NMR** ($CDCl_3$, 600 MHz) δ 7.62-7.61 (m, 2H), 7.57-7.54 (m, 1H), 7.46 (t, J = 7.2 Hz, 1H), 7.41-7.31 (m, 4H), 7.21-7.18 (m, 1H), 4.81 (d, J = 10.7 Hz, 1H), 3.79 (d, J = 10.7 Hz, 1H), 3.42 (s, 3H); **^{13}C-NMR** ($CDCl_3$, 150 MHz) δ 170.4, 170.2, 144.0, 138.8, 131.3, 130.5, 130.3, 129.5, 128.7, 128.2, 123.7, 121.0, 56.9, 34.8.

Note, the reaction was repeated with yields of 45–78%.

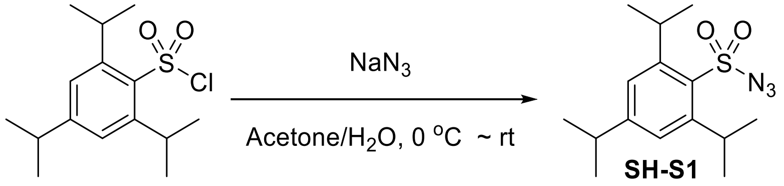

2,4,6-Triisopropylbenzenesulfonyl azide

To a solution of 2,4,6-triisopropylbenzene-1-sulfonyl chloride (100 mg, 0.33 mmol) in acetone (2 mL) at 0 °C was added a solution of NaN_3 (25.8 mg, 0.40 mmol) in water (1 mL). After 30 minutes, the mixture was warmed to room temperature and maintained for 1.5 h. After completion of the reaction, the mixture was diluted with DCM (10 mL) and washed with water (10 mL). The aqueous layer was washed with DCM (2 × 5 mL) and the combined organic extracts were washed with brine, dried over anhydrous Na_2SO_4, filtered, and concentrated. The residue was purified by column chromatography on silica gel (EtOAc/hexane, 5:95 to 10:90) to afford **SH-S1** (96.7 mg, 94%) as a yellow solid. **^{1}H-NMR** ($CDCl_3$, 600 MHz) δ 7.23 (s, 2H), 4.08-4.03 (m, 2H), 2.96-2.91 (m, 1H), 1.29 (d, J = 6.9 Hz, 12H), 1.27 (d, J = 6.9 Hz, 6H); **^{13}C-NMR** ($CDCl_3$, 150 MHz) δ 154.8, 150.8, 132.0, 124.1, 34.3, 29.8, 24.7, 23.5.

Note, the reaction was repeated with yields of 87–94%.

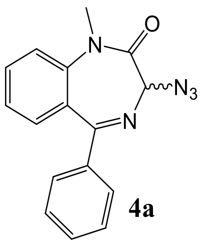

4a

3-Azido-1-methyl-5-phenyl-1H-benzo[e][1,4]diazepin-2(3H)-one

To solution of **3a** (60 mg, 0.24 mmol) in THF (2 mL) at – 78 °C was added 0.5 M KHMDS in toluene (580 µL, 0.29 mmol). After 15 minutes, 2,4,6-triisopropylbenzenesulfonyl azide (**SH-S1,** 150 mg, 0.48 mmol) in THF was added in the mixture. After 5 minutes, AcOH (55 µL, 0.97 mmol) was added and reaction was warmed to 30 °C for 2 h. After the completion of the reaction, aqueous $NaHCO_3$ (10 mL) was added. The mixture was extracted with DCM (2 x 10 mL), dried

over anhydrous Na_2SO_4, filtered and concentrated. The residue was purified by column chromatography on silica gel (EtOAc/hexane, 20:80 to 40:60) to afford **4a** (66 mg, 90%) as a white solid. ^{1}**H-NMR** ($CDCl_3$, 600 MHz) δ 7.70 (d, J = 7.6 Hz, 2H), 7.61 (t, J = 7.7 Hz, 1H), 7.50 (t, J = 7.4 Hz, 1H), 7.41 (dt, J = 19.1, 7.6 Hz, 4H), 7.25 (t, J = 7.7 Hz, 1H), 4.56 (s, 1H), 3.47 (s, 3H); ^{13}**C-NMR** ($CDCl_3$, 150 MHz) δ 167.1, 167.0, 142.8, 137.3, 132.1, 131.0, 130.2, 129.8, 128.6, 128.2, 124.4, 121.5, 75.8, 35.3.

Note, the reaction was repeated with yields of 80–85%.

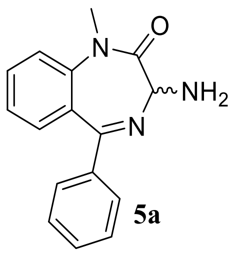

5a

3-Amino-1-methyl-5-phenyl-1H-benzo[e][1,4]diazepin-2(3H)-one

To solution of **4a** (60 mg, 0.21 mmol) in THF (2 mL) and H_2O (70 µL) was added PPh_3 (162 mg, 0.62 mmol) and stirred at room temperature for 24 h. After completion of reaction, the mixture was diluted with EtOAc (10 mL) and washed with NH_4Cl (5 mL). The aqueous layer was washed with EtOAc (2 × 5 mL), and the combined organic extracts were washed with brine, dried over anhydrous Na_2SO_4, filtered and concentrated. The residue was purified by column chromatography on silica gel (MeOH/DCM, 5:95 to 10:90) to afford **5a** (42 mg, 76%) as a yellow solid. ^{1}**H-NMR** ($CDCl_3$, 600 MHz) δ 7.62 (d, J = 7.6 Hz, 2H), 7.58 (t, J = 7.7 Hz, 1H), 7.46 (t, J = 7.2 Hz, 1H), 7.41-7.33 (m, 4H), 7.21 (t, J = 7.4 Hz, 1H), 4.48 (s, 1H), 3.47 (s, 3H), 2.37 (s, 2H); ^{13}**C-NMR** ($CDCl_3$, 150 MHz) δ 170.4, 166.0, 143.2, 138.2, 131.6, 130.4, 130.2, 129.6, 129.2, 128.2, 124.0, 121.3, 70.3, 35.2.

Note, the reaction was repeated with yields of 85–98%.

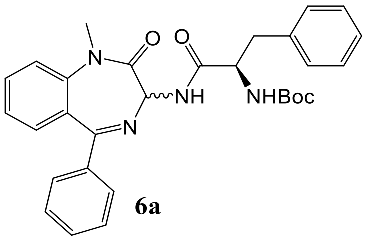

6a

***tert*-Butyl ((2R)-1-((1-methyl-2-oxo-5-phenyl-2,3-dihydro-1H-benzo[e][1,4]diazepin-3-yl)amino)-1-oxo-3-phenylpropan-2-yl)carbamate**

To solution of **5a** (40 mg, 0.15 mmol) in DMF (2 mL, DCM was used for scale-up) was added Boc-D-Phe (44 mg, 0.166 mmol), EDC·HCl (31.8 mg, 0.166 mmol), HOBt hydrate (22.5 mg, 0.166 mmol) and triethylamine (42 µl, 0.30 mmol). The reaction was maintained at room temperature for overnight. After completion, the mixture was diluted with water (20 mL). The aqueous layer was washed with EtOAc (2 × 20 mL), and the combined organic extracts were washed with brine, dried over anhydrous Na_2SO_4, filtered, and concentrated. The residue was purified by column chromatography on silica gel (EtOAc/DCM, 5:95 to 15:85) to afford **6a** (33 mg, 42%) as a white solid. **^{1}H-NMR** ($CDCl_3$, 600 MHz) Note: data were reported as major [minor] if the corresponding peaks were separated from each other or as a range for both of the major and minor diastereomers if the peaks were overlapping. δ 7.81 (d, J = 6.2 Hz, $1H_{minor}$), 7.65 (d, J = 7.9 Hz, $1H_{major}$), 7.60 (t, J = 7.1 Hz, 3H), 7.47 (dd, J = 12.9, 6.0 Hz, 1H), 7.40 (q, J = 7.1 Hz, 4H), 7.32-7.28 (m, 5H), 7.24 (d, J = 7.6 Hz, 1H), 5.50-5.47 (m, 1H), 5.01 (m, J = 7.6 Hz, 1H), 4.60 (m, 1H), 3.45 (s, 3H), 3.10-3.28 (m, 2H), 1.40 [1.41] (s, 9H); **^{13}C-NMR** ($CDCl_3$, 150 MHz) (Note: Due to many similar peaks and intensities, all peaks were written without assignment for major or

minor diastereomers): δ 171.4, 167.5, 167.4, 167.2, 142.7, 138.0, 137.9, 136.6, 136.5, 131.9, 130.7, 130.7, 130.6, 130.6, 129.7, 129.6, 129.5, 128.9, 128.9, 128.5, 128.5, 128.2, 126.8, 126.8, 124.5, 121.6, 121.6, 67.4, 67.2, 55.5, 55.4, 38.5, 38.3, 35.4, 35.4, 28.2.

Note, the reaction was repeated with yields of 60–85%.

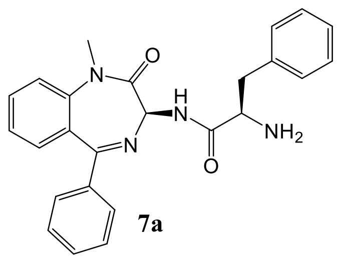

7a

(R)-2-Amino-N-((S)-1-methyl-2-oxo-5-phenyl-2,3-dihydro-1H-benzo[e][1,4]diazepin-3-yl)-3-phenylpropanamide

6a (200 mg) was dissolved in anhydrous EtOAc (2 mL). The mixture was cooled to 0 °C and then saturated with $HCl_{(g)}$ for 1 h. The precipitant was collected and washed with anhydrous EtOAc (3 x 5 mL). The resulting solid was placed in absolute ethanol (3 mL) and heated until all solids dissolved. Then the solution was allowed to cool to room temperature for inducing crystallization/precipitation. The resulting crystals/precipitated solids were collected and washed with 60% diethyl ether in absolute ethanol (3 × 10 mL) and 100% diethyl ether (3 x 10 mL). The collection of precipitants was repeated three times from the mother liquor. The collected solids were combined and basified with 10% NaOH (10 mL) and extracted with EtOAc (10 mL). The aqueous layer was washed with EtOAc (2 × 10 mL) and the combined organic extracts were washed with brine, dried over anhydrous Na_2SO_4, filtered and concentrated to give **7a** (40 mg, 24%) as a yellow solid. **^{1}H-NMR** ($CDCl_3$, 600 MHz) δ 8.98 (d, J = 8.3 Hz, 1H), 7.62-7.59 (m, 3H), 7.47 (t, J = 7.1 Hz, 1H), 7.41-7.33 (m, 6H), 7.28-7.23 (m, 4H), 5.54 (d, J = 8.3 Hz, 1H), 3.75

(d, J = 10.0 Hz, 1H), 3.49 (s, 3H), 3.37 (d, J = 13.8 Hz, 1H), 2.70-2.66 (m, 1H), 1.59 (s, 2H); **^{13}C-NMR** ($CDCl_3$, 150 MHz) δ 174.8, 167.8, 167.6, 142.9, 138.2, 138.1, 131.9, 130.7, 130.6, 129.8, 129.3, 129.0, 128.7, 128.2, 126.7, 124.4, 121.5, 67.2, 56.8, 40.9, 35.3.

Note, the reaction was repeated with yields of 24–35%.

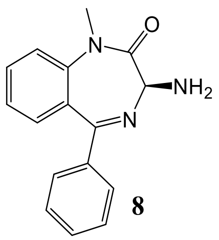

8

(S)-3-Amino-1-methyl-5-phenyl-1H-benzo[e][1,4]diazepin-2(3H)-one

To solution of **7a** (30 mg, 0.073 mmol) in DCM (2 mL) was added PhNCS (9.56 µL, 0.08 mmol). The mixture was heated at 30 °C for 1 h and 40 °C for 30 minutes. After completion of the reaction, the mixture was concentrated and the residue was purified by column chromatography on silica gel (EtOAc/DCM, 5:95 to 10:90) to afford the intermediate thiourea as a white foam. This intermediate was dissolved in TFA (2 mL) and heated at 50 °C for 1 h. After completion of the reaction, the mixture was allowed to cool to room temperature and concentrated several times with DCM (3 x 5 mL). The residue was purified by column chromatography on silica gel (MeOH/DCM, 5:95 to 15:95) to afford **8** (17 mg, 88%) as a purple solid. **^{1}H-NMR** ($CDCl_3$, 600 MHz) δ 7.62 (d, J = 7.6 Hz, 2H), 7.58 (t, J = 7.7 Hz, 1H), 7.47-7.33 (m, 5H), 7.21 (t, J = 7.6 Hz, 1H), 4.49 (s, 1H), 3.47 (s, 3H), 2.55 (s, 2H); **^{13}C-NMR** ($CDCl_3$, 150 MHz) δ 170.4, 166.1, 143.2, 138.2, 131.6, 130.5, 130.2, 129.6, 129.2, 128.2, 124.0, 121.3, 70.2, 35.2.

Note, the reaction was repeated with yields of 49–84%. Also this material was used immediately since racemization was noted upon storage.

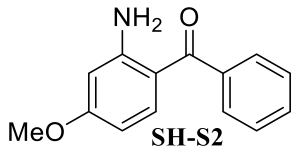
SH-S2

(2-Amino-4-methoxyphenyl)(phenyl)methanone

BCl_3 (1M in DCM, 1.8 mL, 1.78 mmol) was added dropwise to a solution of *m*-anisidine (181 µL, 1.62 mmol) in tetrachloroethane at 0 °C and stirred for 15 min. Benzonitrile (320 µL, 3.24 mmol) and $AlCl_3$ (240 mg, 1.62 mmol) were added and mixture was allowed to warm to room temperature over 30 minutes and then heated at reflux for 6 h. After completion of the reaction, the mixture was cooled to 0 °C and 1.5 N HCl (2 mL) was added dropwise before the mixture was heated to 80 °C for 30 minutes. The mixture was allowed to cool to room temperature and then extracted with DCM (2 x 10 mL). The combined organic extracts were concentrated and purified by column chromatography on silica gel (EtOAc/hexane, 15:85 to 30:70 to afford **SH-S2** (255 mg, 69%) as a dark brown solid. **^{1}H-NMR** ($CDCl_3$, 600 MHz) δ 7.58-7.39 (m, 6H), 6.84 (s, 2H), 6.22 (dd, J = 6.9, 2.4 Hz, 2H), 3.83 (s, 3H); **^{13}C-NMR** ($CDCl_3$, 150 MHz) δ 198.3, 164.8, 152.9, 140.0, 137.2, 130.8, 128.8, 128.1, 112.5, 105.0, 99.8, 55.37.

Note, the reaction was repeated with yields of 45–69%.

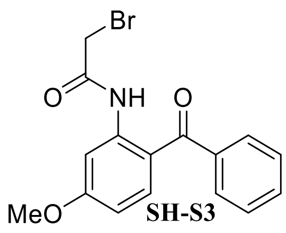

SH-S3

N-(2-Benzoyl-5-methoxyphenyl)-2-bromoacetamide

To solution of **SH-S2** (300 mg, 1.32 mmol) in DCM (5 mL) was added TEA (211 µL, 1.51 mmol). Bromoacetyl chloride (90 µL, 1.11 mmol) was added dropwise at 0 °C and then the mixture was allowed to warm to room temperature for 6 h. After completion of the reaction, the mixture was diluted with water (10 mL) and extracted with EtOAc (10 mL). The aqueous layer was washed with EtOAc (2 × 10 mL) and the combined organic extracts were washed with brine, dried over anhydrous Na_2SO_4, filtered and concentrated. The residue was purified by column chromatography on silica gel (EtOAc/hexane, 5:95 to 10:90) to afford **SH-S3** (480 mg, 82%) as a light-yellow solid. **^{1}H-NMR** ($CDCl_3$, 600 MHz) δ 12.19 (s, 1H), 8.35 (s, 1H), 7.65 (d, J = 7.9 Hz, 2H), 7.59-7.56 (m, 2H), 7.48 (t, J = 7.6 Hz, 2H), 6.63 (d, J = 8.6 Hz, 1H), 4.05 (s, 2H), 3.91 (s, 3H); **^{13}C-NMR** ($CDCl_3$, 150 MHz) δ 198.7, 165.5, 164.4, 142.8, 139.1, 136.3, 131.9, 129.4, 128.2, 116.2, 109.6, 105.2, 55.73, 29.61.

Note, the reaction was repeated with yields of 62–82%.

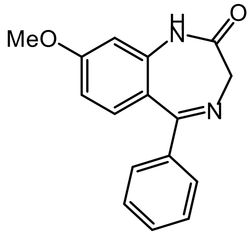

2b

8-Methoxy-5-phenyl-1H-benzo[e][1,4]diazepin-2(3H)-one

To solution of **SH-S3** (150 mg, 0.43 mmol) in MeOH (1 mL) was added 7 N NH_3 in MeOH (3 mL) at room temperature. The mixture was stirred for overnight, then concentrated and purified by column chromatography on silica gel (MeOH/DCM, 5:95 to 10:90) to afford **2b** (41 mg, 35%) as a light-yellow solid. **^{1}H-NMR** ($CDCl_3$, 600 MHz) 8.43 (s, 1H), 7.53 (d, J = 7.6 Hz, 2H), 7.44 (q, J = 7.2 Hz, 1H), 7.39 (t, J = 7.8 Hz, 2H), 7.24 (d, J = 8.6 Hz, 1H), 6.71 (dd, J = 9.0, 1.7 Hz, 1H), 6.58 (s, 1H), 4.32 (s, 2H), 3.88 (s, 3H); **^{13}C-NMR** ($CDCl_3$, 150 MHz) 171.8, 171.0, 161.9, 140.4, 139.7, 132.9, 130.2, 129.7, 128.1, 120.2, 110.7, 104.7, 56.75, 55.64.

Note, the reaction was repeated with yields of 35–86%.

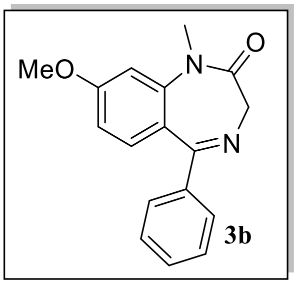

8-Methoxy-1-methyl-5-phenyl-1H-benzo[e][1,4]diazepin-2(3H)-one

To a solution of **2b** (50 mg, 0.187 mmol) in THF (4 mL) was added NaH (60% mineral oil, 9 mg, 0.375 mmol). The mixture was stirred at room temperature for 1.5 h. MeI (17 µL, 0.281 mmol) was added and the mixture was stirred for overnight. After completion of the reaction, the mixture was concentrated, diluted with water (10 mL) and extracted with EtOAc (10 mL). The aqueous layer was washed with EtOAc (2 × 10 mL) and the combined organic extracts were washed with brine, dried over anhydrous Na_2SO_4, filtered and concentrated. The residue was purified by column chromatography on silica gel (MeOH/DCM, 5:95 to 10:90) to afford **3b** (38 mg, 63%) as a yellow solid. **^{1}H-NMR** ($CDCl_3$, 600 MHz) δ 7.60 (d, J = 7.2 Hz, 2H), 7.45 (t, J =

7.4 Hz, 1H), 7.39 (t, J = 7.6 Hz, 2H), 7.23 (d, J = 8.6 Hz, 1H), 6.81 (d, J = 2.1 Hz, 1H), 6.73 (dd, J = 8.6, 1.4 Hz, 1H), 4.78 (d, J = 10.7 Hz, 1H), 3.89 (d, J = 8.8 Hz, 3H), 3.80 (d, J = 10.7 Hz, 1H), 3.41 (s, 3H); **^{13}C-NMR** ($CDCl_3$, 150 MHz) δ 170.1, 161.6, 145.6, 139.1, 132.1, 130.2, 129.6, 128.1, 121.9, 109.7, 106.1, 57.1, 55.6, 35.00.

Note, the reaction was repeated with yields of 34–82%.

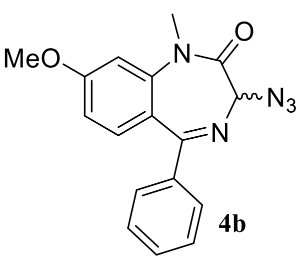

4b

3-Azido-8-methoxy-1-methyl-5-phenyl-1H-benzo[e][1,4]diazepin-2(3H)-one

To a solution of **3b** (300 mg, 1.07 mmol) in THF (10 mL) at – 78 °C was added 0.7 M KHMDS in toluene (1.83 mL, 1.28 mmol). After 15 minutes, 2,4,6-triisopropylbenzenesulfonyl azide (**SH-S1**, 662 mg, 2.14 mmol) in THF (2 mL) was added to the mixture. After 30 minutes, AcOH (245 µL, 4.28 mmol) was added and reaction was warmed to 30 °C for 2 h. After completion of the reaction, aqueous $NaHCO_3$ (10 mL) was added and then the mixture was extracted with DCM (2 x 10 mL). The organic extracts were combined, dried over anhydrous Na_2SO_4, filtered and concentrated. The residue was purified by column chromatography on silica gel (EtOAc/DCM, 5:95 to 10:90) to afford **4b** (295 mg, 85%) as a light-yellow solid. **^{1}H-NMR** ($CDCl_3$, 600 MHz) δ 7.69 (d, J = 7.2 Hz, 2H), 7.49 (t, J = 7.4 Hz, 1H), 7.42 (t, J = 7.6 Hz, 2H), 7.30 (d, J = 9.0 Hz, 1H), 6.83 (d, J = 2.1 Hz, 1H), 6.78 (dd, J = 8.8, 2.2 Hz, 1H), 4.59 (s, 1H), 3.91 (s, 3H), 3.46 (s, 3H); **^{13}C-NMR** ($CDCl_3$, 150 MHz) 167.0, 166.8, 162.2, 144.6, 137.7, 132.0, 130.9, 130.0, 128.3, 121.7, 110.5, 106.6, 75.9, 55.7, 35.5.

Note, the reaction was repeated with yields of 80–85%.

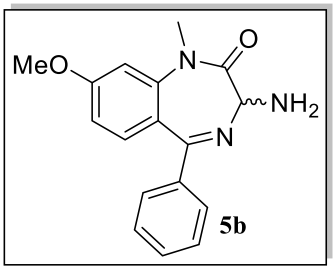

5b

3-Amino-8-methoxy-1-methyl-5-phenyl-1H-benzo[e][1,4]diazepin-2(3H)-one

To a solution of **4b** (295 mg, 0.918 mmol) in THF (10 mL) and H_2O (350 µL) was added PPh_3 (772 mg, 2.75 mmol). The mixture was stirred at room temperature for 24 h. After completion of the reaction, the mixture was diluted with EtOAc (10 mL) and washed with NH_4Cl (5 mL). The aqueous layer was washed with EtOAc (2 × 5 mL) and the combined organic extracts were washed with brine, dried over anhydrous Na_2SO_4, filtered and concentrated. The residue was purified by column chromatography on silica gel (MeOH/DCM, 5:95 to 10:90) to afford **5b** (220 mg, 81%) as a light wheat solid. **^{1}H-NMR** ($CDCl_3$, 600 MHz) δ 7.61 (d, J = 7.6 Hz, 2H), 7.46-7.44 (m, 1H), 7.39 (t, J = 7.6 Hz, 2H), 7.25 (d, J = 8.6 Hz, 1H), 6.82 (d, J = 2.1 Hz, 1H), 6.74 (dd, J = 8.6, 2.1 Hz, 1H), 4.49 (s, 1H), 3.90 (s, 3H), 3.46 (s, 3H), 2.36 (s, 2H); **^{13}C-NMR** ($CDCl_3$, 150 MHz) δ 170.2, 166.0, 161.8, 144.8, 138.5, 131.8, 130.3, 129.7, 128.2, 122.4, 110.0, 106.3, 70.42, 55.67, 35.40.

Note, the reaction was repeated with yields of 81–98%.

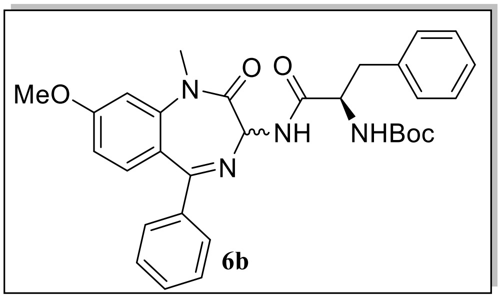

6b

***tert*-Butyl ((2R)-1-((8-methoxy-1-methyl-2-oxo-5-phenyl-2,3-dihydro-1H-benzo[e][1,4]diazepin-3-yl)amino)-1-oxo-3-phenylpropan-2-yl)carbamate**

To a solution of **5b** (50 mg, 0.156 mmol) in DCM (4 mL) were added Boc-D-Phe (40 mg, 0.186 mmol), EDC·HCl (36 mg, 0.186 mmol), HOBt hydrate (26 mg, 0.186 mmol) and triethylamine (43 µL, 0.312 mmol). The reaction mixture was stirred at room temperature for overnight. After completion of the reaction, the mixture was diluted with water (10 mL). The aqueous layer was washed with EtOAc (2 × 10 mL) and the combined organic extracts were washed with brine, dried over anhydrous Na_2SO_4, filtered and concentrated. The residue was purified by column chromatography on silica gel (EtOAc/DCM, 5:95 to 15:85) to afford **6b** (72 mg, 85%) as a yellow solid. **^{1}H-NMR** ($CDCl_3$, 600 MHz) (Note: data were reported as major [minor] if the corresponding peaks were separated from each other or as a range for both of the major and minor diastereomers if the peaks were overlapping. δ 7.79 (d, J = 6.9 Hz, 1H), 7.69-7.63 (m, 1H), 7.59 (t, J = 7.7 Hz, 2H), 7.46 (q, J = 6.7 Hz, 1H), 7.39 (q, J = 7.2 Hz, 2H), 7.33-7.27 (m, 5H), 7.24 (d, J = 7.2 Hz, 1H), 6.83 (s, 1H), 6.78 (d, J = 9.0 Hz, 1H), 5.51-5.48 (m, 1H), 5.03 (dd, J = 17.9, 7.9 Hz, 1H), 4.60 (s, 1H), 3.91 [3.90] (s, 3H), 3.44 (s, 3H), 3.29-3.09 (m, 2H), 1.40 [1.41] (s, 9H); **^{13}C-NMR** ($CDCl_3$, 150 MHz) (Note: Due to many similar peaks and intensities, all peaks were written without assignment for major or minor diastereomers): δ 171.3, 167.3, 167.3, 167.0, 144.4, 138.3, 138.2, 136.6, 136.5, 132.3, 132.3, 132.1, 132.0, 131.9, 130.6, 130.6, 129.8,

129.6, 129.5, 128.5, 128.5, 128.4, 128.2, 126.8, 126.8, 122.0, 110.5, 106.7, 67.5, 67.3, 55.7, 55.5, 53.4, 38.5, 38.4, 35.5, 35.5, 28.2.

Note, the reaction was repeated with yields of 60–85%.

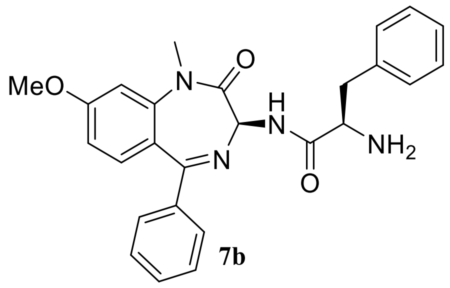

7b

(R)-2-Amino-N-((S)-8-methoxy-1-methyl-2-oxo-5-phenyl-2,3-dihydro-1H-benzo[e][1,4]diazepin-3-yl)-3-phenylpropanamide

6b (500 mg, 1.13 mmol) was dissolved in anhydrous EtOAc (10 mL) and cooled to 0 °C. The mixture was saturated with $HCl_{(g)}$. After 1 h, the precipitant was collected and washed with anhydrous EtOAc (3 x 5 mL). The solid was placed in absolute ethanol (4 mL) and then the mixture was heated until all solids dissolved. The solution was allowed to cool to room temperature for inducing crystallization/precipitation. The resulting crystals/precipitated solids were collected and washed with 60% diethyl ether in absolute ethanol (2 × 10 mL) and 100% diethyl ether (2 × 10 mL). Precipitation collection was repeated three times from mother liquor. The combined solids were basified with 10% NaOH (10 mL) and extracted with EtOAc (10 mL). The aqueous layer was washed with EtOAc (2 × 10 mL), and the combined organic extracts were washed with brine, dried over anhydrous Na_2SO_4, filtered and concentrated to give **7b** (120 mg, 29%) as a light yellow solid. **^{1}H-NMR** ($CDCl_3$, 600 MHz) 8.95 (d, J = 8.3 Hz, 1H), 7.60 (d, J = 7.2 Hz, 2H), 7.46 (t, J = 7.2 Hz, 1H), 7.36 (dt, J = 29.8, 7.5 Hz, 4H), 7.29-7.27 (m, 4H), 6.84 (d, J = 2.4 Hz, 1H), 6.78 (dd,

J = 8.6, 2.4 Hz, 1H), 5.55 (d, J = 8.3 Hz, 1H), 3.91 (s, 3H), 3.74 (dd, J = 10.2, 3.6 Hz, 1H), 3.47 (s, 3H), 3.37 (dd, J = 13.8, 3.4 Hz, 1H), 2.69 (dd, J = 13.6, 10.5 Hz, 1H), 1.55 (s, 2H); **^{13}C-NMR** ($CDCl_3$, 150 MHz) 174.7, 167.6, 167.5, 162.1, 144.5, 138.4, 138.2, 132.3, 130.5, 129.8, 129.3, 128.7, 128.2, 126.7, 122.2, 110.5, 106.7, 67.2, 56.8, 55.7, 40.9, 35.5.

Note, the reaction was repeated with yields of 24–35%.

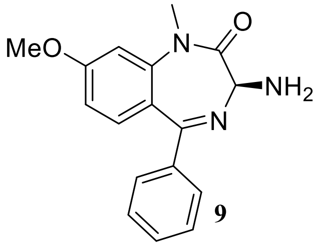

9

(S)-3-Amino-8-methoxy-1-methyl-5-phenyl-1H-benzo[e][1,4]diazepin-2(3H)-one

To a solution of **7b** (170 mg, 0.384 mmol) in DCM (5 mL) was added PhNCS (55 µL, 0.46 mmol). The mixture was heated to 30 °C for 1 h and 40 °C for 30 minutes. The reaction mixture was concentrated, and the residue purified by column chromatography on silica gel (EtOAc/DCM, 5:95 to 10:90) to afford intermediate thiourea as a white foam. This intermediate was dissolved in TFA (5 mL), and the solution was heated to 50 °C for 1 h. After the completion of the reaction, the mixture was allowed to cool to room temperature and then concentrated several times with DCM (3 x 5 mL). The residue was purified by column chromatography on silica gel (MeOH/DCM, 5:95 to 15:85) to afford **9** (76 mg, 67%) as a light purple solid. **^{1}H-NMR** ($CDCl_3$, 600 MHz) δ 7.61 (d, J = 7.2 Hz, 2H), 7.45 (t, J = 7.2 Hz, 1H), 7.39 (t, J = 7.6 Hz, 2H), 7.25 (d, J = 9.0 Hz, 1H), 6.82 (d, J = 2.1 Hz, 1H), 6.74 (dd, J = 9.0, 2.1 Hz, 1H), 4.48 (s, 1H), 3.90 (s, 3H), 3.46 (s, 3H); **^{13}C-NMR** ($CDCl_3$, 150 MHz) δ 170.2, 166.0, 161.8, 144.8, 138.5, 131.8, 130.3, 129.7, 128.2,

122.4, 110.0, 106.3, 70.4, 55.6, 35.3. This material was used immediately since racemization was noted for compound **8**.

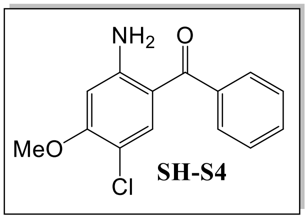

(2-amino-5-chloro-4-methoxyphenyl)(phenyl)methanone

BCl_3 (1M in DCM, 5.32 mL, 5.32 mmol) was added dropwise to a solution of 4-chloro-3-methoxyaniline (700 mg, 4.44 mmol) in tetrachloroethane at 0 °C and stirred for 15 min. Benzonitrile (914 µL, 8.88 mmol) and $AlCl_3$ (592 mg, 4.44 mmol) were added and mixture was allowed to warm to room temperature over 30 minutes and then heated at reflux for 6 h. After completion of the reaction, the mixture was cooled to 0 °C and 1.5 N HCl (10 mL) was added dropwise before the mixture was heated to 80 °C for 30 minutes. The mixture was allowed to cool to room temperature and then extracted with DCM (2 x 30 mL). The combined organic extracts were concentrated and purified by column chromatography on silica gel (EtOAc/hexane, 15:85 to 30:70 to afford **SH-S4** (998 mg, 86%) as a brown solid. **^{1}H-NMR** ($CDCl_3$, 600 MHz) δ 7.58 (d, J = 7.0 Hz, 2H), 7.53 (m, 6H), 7.47 (m, 3H), 6.41 (s, 2H), 6.20 (s, 1H), 3.92 (s, 3H); **^{13}C-NMR** ($CDCl_3$, 150 MHz) δ 196.9, 159.5, 152.2, 139.9, 135.6, 130.9, 128.7, 128.2, 112.0, 109.6, 98.8, 56.16.

Note, the reaction was repeated with yields of 72–86%.

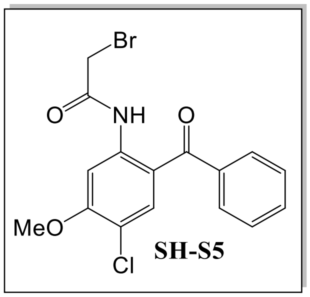

N-(2-benzoyl-4-chloro-5-methoxyphenyl)-2-bromoacetamide

To solution of **SH-S4** (1 g, 3.82 mmol) in ACN (5 mL) was added Na_2CO_3 (607 mg, 5.73 mmol). Bromoacetyl chloride (350 µL, 4.2 mmol) was added dropwise at 0 °C and then the mixture was allowed to warm to room temperature and stirred 8 hours. After completion of the reaction, the mixture was diluted with water (50 mL) and extracted with EtOAc (30 mL). The aqueous layer was washed with EtOAc (2 × 20 mL) and the combined organic extracts were washed with brine, dried over anhydrous Na_2SO_4, filtered and concentrated. The residue was purified by column chromatography on silica gel (EtOAc/hexane, 5:95 to 10:90) to afford **SH-S5** (1.09 g, 74%) as a yellow solid. **^{1}H-NMR** ($CDCl_3$, 600 MHz) δ 12.1 (s, 1H), 8.50 (s, 1H), 7.66 (d, J = 7.2 Hz, 2H), 7.63 (s, 1H), 7.61 (t, J = 7.5 Hz, 1H), 7.52 (t, J = 7.7 Hz, 2H), 4.05 (s, 2H), 4.03 (s, 3H); **^{13}C-NMR** ($CDCl_3$, 150 MHz) δ 197.9, 165.5, 159.4, 141.4, 138.4, 135.2, 132.3, 129.4, 128.5, 116.5, 116.2, 104.2, 56.67, 29.51.

Note, the reaction was repeated with yields of 58–74%.

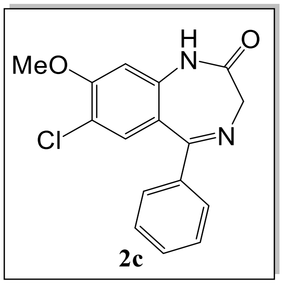

7-chloro-8-methoxy-5-phenyl-1H-benzo[e][1,4]diazepin-2(3H)-one

To solution of **SH-S5** (1 g, 2.61 mmol) in MeOH (5 mL) was added 7 N NH_3 in MeOH (20 mL) at room temperature. The mixture was stirred for overnight. After the completion of reaction, it was concentrated and purified by column chromatography on silica gel (MeOH/DCM, 5:95 to 10:90) to afford **2c** (630 mg, 80%) as a light-yellow solid. **^{1}H-NMR** ($CDCl_3$, 600 MHz) δ 9.68 (s, 1H), 7.52 (d, J = 7.2 Hz, 2H), 7.47 (t, J = 7.4 Hz, 1H), 7.40 (t, J = 7.6 Hz, 2H), 7.31 (s, 1H), 6.68 (s, 1H), 4.33 (Br, s, 2H), 3.97 (s, 3H); **^{13}C-NMR** ($CDCl_3$, 150 MHz) δ 171.6, 169.9, 157.2, 139.0, 138.9, 132.3, 130.5, 129.6, 128.3, 120.5, 117.8, 103.8, 56.7, 56.5.

Note, the reaction was repeated with yields of 80–82%.

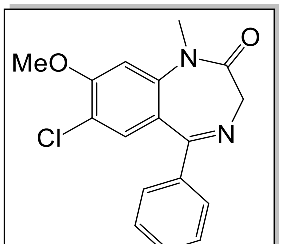

3c

7-chloro-8-methoxy-1-methyl-5-phenyl-1H-benzo[e][1,4]diazepin-2(3H)-one

To a solution of **2c** (520 mg, 1.73 mmol) in THF (10 mL) was added NaH (60% mineral oil, 83 mg, 3.46 mmol). The mixture was stirred at room temperature for 1.5 h. MeI (161 µL, 2.59 mmol) was added and the mixture was stirred for overnight. Additional NaH was added (120 mg, because reaction was not complete). After completion of the reaction, the mixture was concentrated, diluted with water (30 mL) and extracted with EtOAc (20 mL). The aqueous layer was washed with EtOAc (2 × 20 mL) and the combined organic extracts were washed with brine, dried over anhydrous Na_2SO_4, filtered and concentrated. The residue was purified by column chromatography on silica gel (MeOH/DCM, 5:95 to 10:90) to afford **3c** (512 mg, 94%) as a yellow

solid. **¹H-NMR** ($CDCl_3$, 600 MHz) δ 7.60 (d, J = 7.6 Hz, 2H), 7.48 (t, J = 7.3 Hz, 1H), 7.41 (t, J = 7.6 Hz, 2H), 7.30 (s, 1H), 6.81 (s, 1H), 4.82 (d, J = 10.7 Hz, 1H), 4.01 (s, 3H), 3.79 (d, J = 10.7 Hz, 1H), 3.42 (s, 3H); **¹³C-NMR** ($CDCl_3$, 150 MHz) δ 169.8, 169.0, 156.8, 144.1, 138.4, 131.5, 130.6, 129.5, 128.3, 122.2, 118.2, 104.1, 57.0, 56.4, 35.1.

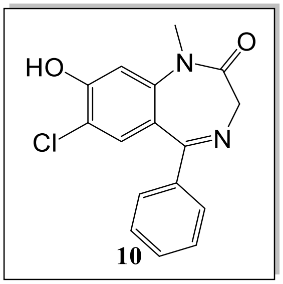

7-chloro-8-hydroxy-1-methyl-5-phenyl-1H-benzo[e][1,4]diazepin-2(3H)-one

To a solution of **3c** (250 mg, 0.794 mmol) in DCM (1 mL) was added 1M BBr_3 (1 mL) dropwise at 0 °C. The reaction was maintained at room temperature for overnight. Additional BBr_3 (2 mL) was added. After completion of the reaction, the mixture was quenched by saturated aqueous Na_2CO_3. The aqueous layer was washed with EtOAc (2 × 20 mL) and the combined organic extracts were washed with brine, dried over anhydrous Na_2SO_4, filtered and concentrated. The residue was purified by column chromatography on silica gel (MeOH/DCM, 2:98 to 5:95) to afford **10** (180 mg, 75%) with an estimated purity of 80%, which was used for the next step without further purification. **¹H-NMR** ($CDCl_3$, 600 MHz) δ 7.59 (d, J = 7.2 Hz, 2H), 7.49 (t, J = 7.3 Hz, 1H), 7.42 (t, J = 7.6 Hz, 2H), 7.25 (d, J = 3.3 Hz, 1H), 6.91 (d, J = 5.7 Hz, 1H), 4.78 (d, J = 10.8 Hz, 1H), 3.81 (d, J = 11.0 Hz, 1H), 3.31 (s, 3H).

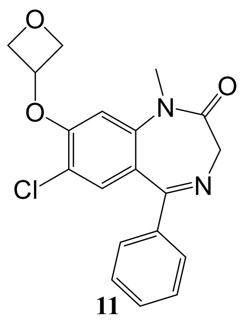

11

7-chloro-1-methyl-8-(oxetan-3-yloxy)-5-phenyl-1H-benzo[e][1,4]diazepin-2(3H)-one

To a solution of **10** (180 mg, 0.598 mmol) in DMF (4 mL) was added 3-bromooxetane (137 µL, 1.79 mmol) and K_2CO_3 (248 mg, 1.79 mmol). The reaction was stirred at 100 °C for overnight. After completion of the reaction, the mixture was diluted with water (30 mL). The aqueous layer was washed with EtOAc (2 × 20 mL) and the combined organic extracts were washed with additional water (30 mL), brine, dried over anhydrous Na_2SO_4, filtered, and concentrated. The residue was purified by column chromatography on silica gel (MeOH/DCM, 5:95 to 10:90) to afford **11** (157 mg, 73%) as a yellow solid. **^{1}H-NMR** ($CDCl_3$, 600 MHz) δ 7.59 (d, J = 7.2 Hz, 2H), 7.49 (t, J = 7.4 Hz, 1H), 7.42 (t, J = 7.6 Hz, 2H), 7.34 (s, 1H), 6.34 (s, 1H), 5.36-5.32 (m, 1H), 5.05 (q, J = 6.8 Hz, 2H), 4.93-4.86 (m, 2H), 4.82 (d, J = 10.8 Hz, 1H), 3.77 (d, J = 10.8 Hz, 1H), 3.36 (s, 3H); **^{13}C-NMR** ($CDCl_3$, 150 MHz) δ 169.7, 168.8, 154.1, 143.9, 138.2, 132.1, 130.7, 129.5, 128.4, 123.1, 118.6, 104.9, 77.3, 71.4, 56.9, 35.0.

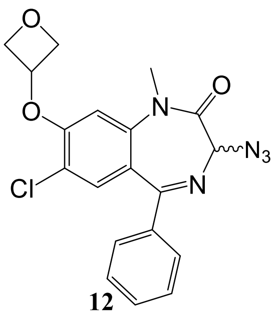

12

3-azido-7-chloro-1-methyl-8-(oxetan-3-yloxy)-5-phenyl-1H-benzo[e][1,4]diazepin-2(3H)-one

To a solution of **11** (160 mg, 0.448 mmol) in THF (10 mL) at – 78 °C was added 0.5 M KHMDS in toluene (1 mL, 0.896 mmol). After 15 minutes, 2,4,6-triisopropylbenzenesulfonyl azide (**SH-S1**, 276 mg, 0.892 mmol) in THF (2 mL) was added to the mixture. After 30 minutes, AcOH (104 µL, 1.79 mmol) was added and reaction was warmed to 30 °C for 2.5 h. After completion of the reaction, aqueous $NaHCO_3$ (20 mL) was added and then the mixture was extracted with DCM (2 x 20 mL). The organic extracts were combined, dried over anhydrous Na_2SO_4, filtered and concentrated. The residue was purified by column chromatography on silica gel (EtOAc/DCM, 5:95 to 10:90) to afford **12** (151 mg, 84%) as a light-yellow solid. **^{1}H-NMR** ($CDCl_3$, 600 MHz) δ 7.67 (d, J = 7.6 Hz, 2H, 7.53 (t, J = 7.3 Hz, 1H), 7.45 (t, J = 7.7 Hz, 2H), 7.41 (s, 1H), 6.36 (s, 1H), 5.37-5.33 (m, 1H), 5.05 (q, J = 6.9 Hz, 2H), 4.88 (ddd, J = 30.0, 7.2, 5.4 Hz, 2H), 4.55 (s, 1H), 3.41 (s, 3H); **^{13}C-NMR** ($CDCl_3$, 150 MHz) δ 166.5, 165.7, 154.7, 142.9, 136.9, 131.9, 131.3, 129.9, 128.5, 123.0, 119.5, 105.3, 77.26, 77.1, 75.8, 71.5, 35.6.

Note, the reaction was repeated with yields of 84–85%.

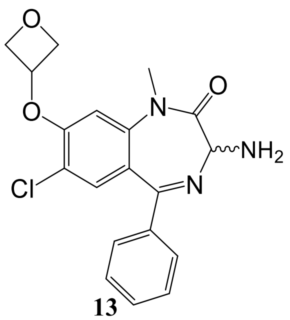

13

3-amino-7-chloro-1-methyl-8-(oxetan-3-yloxy)-5-phenyl-1H-benzo[e][1,4]diazepin-2(3H)-one

To a solution of **12** (50 mg, 0.125 mmol) in THF (3 mL) and H_2O (1 mL) was added PPh_3 (98 mg, 0.377 mmol). The mixture was stirred at room temperature for 24 h. After completion of the reaction, the mixture was diluted with EtOAc (10 mL) and washed with NH_4Cl (5 mL). The aqueous layer was washed with EtOAc (2 × 5 mL) and the combined organic extracts were washed with brine, dried over anhydrous Na_2SO_4, filtered and concentrated. The residue was purified by column chromatography on silica gel (MeOH/DCM, 5:95 to 10:90) to afford **13** (39 mg, 83%) as a light wheat solid. **^{1}H-NMR** ($CDCl_3$, 600 MHz) δ 7.59 (d, J = 8.4 Hz, 2H), 7.49 (t, J = 7.3 Hz, 1H), 7.42 (t, J = 7.6 Hz, 2H), 7.36 (s, 1H), 6.36 (s, 1H), 5.35 (m, 1H), 5.07-5.03 (m, 2H), 4.88 (m, 2H), 4.47 (s, 1H), 3.41 (s, 3H), 2.53 (Br, s, 2H); **^{13}C-NMR** ($CDCl_3$, 150 MHz) δ 164.6, 154.3, 143.1, 137.6, 131.7, 130.8 129.6, 128.4, 123.6, 118.9, 105.1, 77.30, 77.1, 71.4, 35.4.

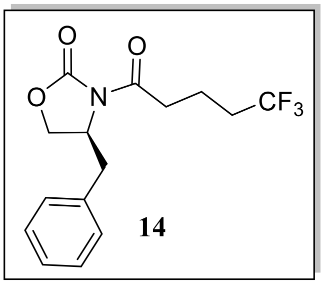

14

(S)-4-Benzyl-3-(5,5,5-trifluoropentanoyl)oxazolidin-2-one

To solution of 5,5,5-trifluoropentanoic acid (70 µL, 0.58 mmol) in DCM (3 mL) were added DMF (2 drops) and oxalyl chloride (50 µL, 0.58 mmol) over 5 minutes. The mixture was stirred at room temperature for 2 h and then concentrated to give an acid chloride intermediate. To a solution of (*S*)-4-benzyloxazolidin-2-one (76 mg, 0.43 mmol) in THF (2 mL) at -78 °C was added solution of 2.5 M n-BuLi in hexane (230 µL, 0.58 mmol) over 5 minutes. The mixture was stirred at -78 °C for 10 minutes and the acid chloride intermediate in THF (3 mL) was added via cannula. The resulting mixture was stirred at -78 °C for 1.5 h and then allowed to cool to room temperature.

After the completion of the reaction, the mixture was diluted with saturated NH_4Cl (10 mL), and the aqueous layer was washed with diethyl ether (2 × 10 mL). The combined organic extracts were washed with 1 M aqueous NaOH, brine, dried over anhydrous Na_2SO_4, filtered and concentrated. The residue was purified by column chromatography on silica gel (EtOAc/hexane, 15:85 to 30:70) to afford **14** (109 mg, 60%) as a clear oil. **^{1}H-NMR** ($CDCl_3$, 600 MHz) δ 7.34 (t, J = 7.4 Hz, 2H), 7.28 (t, J = 7.4 Hz, 1H), 7.21 (d, J = 6.9 Hz, 2H), 4.68 (dq, J = 13.1, 3.6 Hz, 1H), 4.24-4.18 (m, 2H), 3.29 (dd, J = 13.3, 3.3 Hz, 1H), 3.03 (qt, J = 17.4, 7.2 Hz, 2H), 2.78 (dd, J = 13.4, 9.6 Hz, 1H), 2.25-2.17 (m, 2H), 2.00-1.95 (m, 2H); **^{13}C-NMR** ($CDCl_3$, 150 MHz) δ 171.9, 153.4, 135.1, 129.4, 129.0, 127.4, 126.9 (q, J_{CF3} = 274.3 Hz), 66.4, 55.1, 37.9, 34.2, 32.9 (q, J_{CF3} = 28.8 Hz), 16.6, 16.6.

Note, the reaction was repeated with yields of 70–83%.

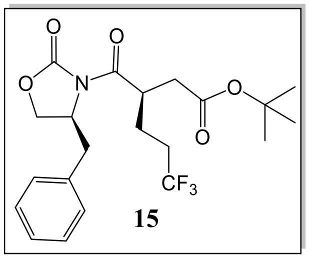

15

(R)-tert-Butyl 3-((S)-4-benzyl-2-oxooxazolidine-3-carbonyl)-6,6,6-trifluorohexanoate

To a solution of **14** (120 mg, 0.38 mmol) in THF (2 mL) at -78 °C was added 1M NaHMDS (419 µL, 0.42 mmol). After 2 h, t-butyl bromoacetate (170 µL, 1.143 mmol) was added over 30 minutes and the resulting mixture was stirred at -78 °C for 9 h. After completion of the reaction, the mixture was allowed to warm to room temperature, diluted with saturated NH_4Cl (10 mL), and extracted with EtOAc (20 mL). The aqueous layer was washed with EtOAc (2 × 10 mL) and the combined organic extracts were washed with brine, dried over anhydrous Na_2SO_4, filtered and

concentrated. The residue was purified by column chromatography on silica gel (EtOAc/hexane, 15:85 to 30:70) to afford **15** (25.8 mg, 16%) as a clear oil. **^{1}H-NMR** ($CDCl_3$, 600 MHz) δ 7.35 (t, J = 7.2 Hz, 2H), 7.27 (dd, J = 17.0, 7.1 Hz, 3H), 4.68 (t, J = 8.6 Hz, 1H), 4.23-4.18 (m, 3H), 3.35-3.33 (m, 1H), 2.84 (dd, J = 16.7, 9.8 Hz, 1H), 2.74 (dd, J = 13.1, 10.3 Hz, 1H), 2.46 (dd, J = 16.7, 4.6 Hz, 1H), 2.19-2.14 (m, 2H), 1.98-1.94 (m, 1H), 1.79-1.75 (m, 1H), 1.44 (d, J = 12.4 Hz, 9H) ; **^{13}C-NMR** ($CDCl_3$, 150 MHz) δ 174.4, 170.5, 153.1, 135.4, 129.4, 128.9, 127.3, 126.6 (q, J_{CF3} = 274.3 Hz), 81.2, 66.1, 55.4, 38.2, 37.4, 37.0, 31.2 (q, J_{CF3} = 30.15 Hz), 28.0, 23.7.

Note, the reaction was repeated with yields of 52–65%. NaHMDS also was replaced with LiHMDS for a few repeats.

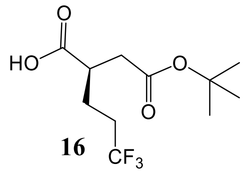

16

(R)-2-(2-(tert-Butoxy)-2-oxoethyl)-5,5,5-trifluoropentanoic acid

To a solution of **15** (50 mg, 0.12 mmol) in THF (3 mL) and water (1 mL) at 0 °C were added 30% aqueous solution of H_2O_2 (47.5 µL, 0.48 mmol) and solution of LiOH (10 mg) in water (1 mL). After 10 minutes, the mixture was allowed to warm to room temperature. After 1.5 h, the reaction was cooled to 0 °C and saturated $NaHCO_3$ (5 mL) and Na_2SO_3 (5 mL) were added. After stirring 15 minutes, the mixture was concentrated (removing THF) and the aqueous layer was washed with DCM (10 mL). The aqueous layer was acidified to pH 3 with 1M HCl. The acidified aqueous layer was extracted with EtOAc (2 x 10 mL) and the combined organic extracts were washed with brine, dried over anhydrous Na_2SO_4, filtered and concentrated to afford **16** (32 mg), which was used in the next step without purification. **^{1}H-NMR** ($CDCl_3$, 600 MHz) δ 2.90-2.85 (m,

1H), 2.67 (q, J = 8.3 Hz, 1H), 2.44 (dd, J = 16.5, 5.5 Hz, 1H), 2.27-2.14 (m, 2H), 1.97-1.90 (m, 1H), 1.84-1.79 (m, 1H), 1.44 (s, 9H); **^{13}C-NMR** ($CDCl_3$, 150 MHz) δ 179.6, 170.3, 126.6 (q, J_{CF3} = 275.8 Hz), 81.6, 40.1, 37.0, 31.4 (q, J_{CF3} = 30.1 Hz), 27.9, 23.5.

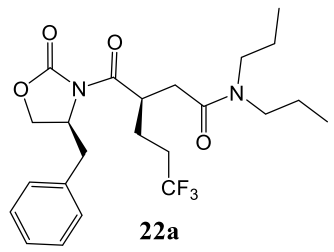

22a

(R)-3-((S)-4-Benzyl-2-oxooxazolidine-3-carbonyl)-6,6,6-trifluoro-N,N-dipropylhexanamide

To a solution of **15** (200 mg, 0.53 mmol) in DCM (2 mL) at 0 °C was added TFA (1 mL). The mixture was stirred at 0 °C for 1 h and at room temperature for 2.5 h. After completion of the reaction, the mixture was diluted with DCM (3 x 5 mL) several times and concentrated to afford a carboxylic acid intermediate (190 mg), which was used in the next step without purification. **^{1}H-NMR** ($CDCl_3$, 600 MHz) δ 10.85 (s, 1H), 7.34-7.18 (m, 5H), 4.68 (dq, J = 13.1, 3.4 Hz, 1H), 4.24-4.16 (m, 3H), 3.26 (dd, J = 13.8, 3.1 Hz, 1H), 2.99 (dd, J = 17.4, 9.8 Hz, 1H), 2.72 (dd, J = 13.4, 9.6 Hz, 1H), 2.56 (dd, J = 17.6, 4.5 Hz, 1H), 2.19-2.09 (m, 2H), 1.99-1.93 (m, 1H), 1.80-1.74 (m, 1H); **^{13}C-NMR** ($CDCl_3$, 150 MHz) δ 177.6, 173.9, 153.2, 135.1, 129.4, 129.0, 127.4, 126.5 (q, J_{CF3} = 270.0 Hz),, 66.2, 55.4, 37.8, 37.2, 35.3, 31.2 (q, J_{CF3} = 28.6 Hz), 23.8. Note, the reaction was repeated with yields of 92–95%.

To solution of the carboxylic acid intermediate (450 mg, 1.21 mmol) in THF (10 mL) at 0 °C were added triethylamine (500 µL, 3.62 mmol) and disuccinimidyl carbonate (617.4 mg, 2.41 mmol). After 15 minutes, the mixture was allowed to warm to room temperature. After 3 h, the precipitated solids were filtered and dipropylamine (413 µL, 6.02 mmol) was added to the filtrate

and stirred for 5 h at room temperature. After completion of the reaction, the organic solvent was concentrated and the residue was purified by column chromatography on silica gel (EtOAc/DCM, 5:95 to 15:85) to afford **22a** (412 mg, 75%) as a clear oil. **^{1}H-NMR** ($CDCl_3$, 600 MHz) δ 7.33 (t, J = 7.4 Hz, 2H), 7.27 (d, J = 4.5 Hz, 3H), 4.66 (qd, J = 7.0, 3.4 Hz, 1H), 4.35 (td, J = 10.5, 6.3 Hz, 1H), 4.20-4.15 (m, 2H), 3.37 (dd, J = 13.6, 2.9 Hz, 1H), 3.31-3.20 (m, 3H), 3.18-3.15 (m, 1H), 3.00 (dd, J = 16.2, 10.7 Hz, 1H), 2.75 (dd, J = 13.6, 10.5 Hz, 1H), 2.48 (dd, J = 16.0, 4.0 Hz, 1H), 2.20 (td, J = 18.7, 10.0 Hz, 2H), 1.97-1.91 (m, 1H), 1.81-1.75 (m, 1H), 1.69-1.58 (m, 2H), 1.50 (td, J = 15.0, 7.3 Hz, 2H), 0.98-0.93 (m, 3H), 0.87-0.82 (m, 3H); **^{13}C-NMR** ($CDCl_3$, 150 MHz) δ 175.3, 169.7, 153.2, 135.9, 129.4, 128.9, 127.1, 126.7 (q, J_{CF3} = 275.8 Hz), 66.0, 55.4, 49.5, 47.6, 38.2, 37.2, 35.7, 31.4 (q, J_{CF3} = 28.6 Hz), 24.0, 22.0, 20.8, 11.3.

Note, the reaction was repeated with yields of 65–75%.

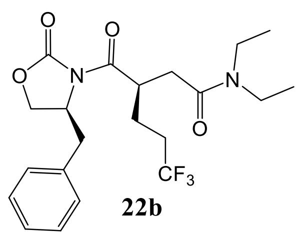

22b

(R)-3-((S)-4-Benzyl-2-oxooxazolidine-3-carbonyl)-N,N-diethyl-6,6,6-trifluorohexanamide

The reaction was performed following the procedure described for **22a** but diethylamine was used. The residue was purified by column chromatography on silica gel (EtOAc/DCM, 5:95 to 15:85) to afford **22b** (385 mg, 70%) as a clear oil. **^{1}H-NMR** ($CDCl_3$, 600 MHz) δ 7.35-7.32 (m, 2H), 7.27-7.25 (m, 6H), 4.67 (qd, J = 7.0, 3.4 Hz, 1H), 4.35 (td, J = 10.7, 6.5 Hz, 1H), 4.20-4.15 (m, 2H), 3.40-3.29 (m, 5H), 2.99 (dd, J = 16.0, 10.8 Hz, 1H), 2.76 (dd, J = 13.8, 10.3 Hz, 1H), 2.49 (dd, J = 16.0, 4.0 Hz, 1H), 2.23-2.16 (m, 2H), 1.97-1.91 (m, 1H), 1.82-1.76 (m, 1H), 1.22 (t, J =

7.2 Hz, 3H), 1.07 (t, J = 7.2 Hz, 3H); **^{13}C-NMR** ($CDCl_3$, 150 MHz) δ 175.3, 169.2, 153.2, 135.9, 129.5, 128.9, 127.1, 126.7 (q, J_{CF3} = 274.3 Hz), 66.0, 55.4, 41.9, 40.3, 38.1, 37.2, 35.7, 31.4 (q, J_{CF3} = 28.8 Hz), 24.1, 14.2, 13.1.

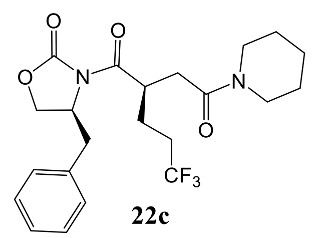

22c

(R)-1-((S)-4-Benzyl-2-oxooxazolidin-3-yl)-4-(piperidin-1-yl)-2-(3,3,3-trifluoropropyl)butane-1,4-dione

The reaction was performed following the procedure described for **22a** but piperidine was used. The residue was purified by column chromatography on silica gel (EtOAc/DCM, 5:95 to 15:85) to afford **22c** (107.7 mg, 58%) as a clear oil. **^{1}H-NMR** ($CDCl_3$, 600 MHz) δ 7.34-7.25 (m, 5H), 4.69-4.66 (m, 1H), 4.34-4.32 (m, 1H), 4.20-4.16 (m, 2H), 3.61-3.58 (m, 1H), 3.45-3.36 (m, 4H), 2.98 (dd, J = 16.0, 10.8 Hz, 1H), 2.78-2.74 (m, 1H), 2.50 (dd, J = 16.0, 3.3 Hz, 1H), 2.20 (dt, J = 28.0, 9.7 Hz, 2H), 1.96-1.90 (m, 1H), 1.82-1.76 (m, 1H), 1.71-1.50 (m, 6H); **^{13}C-NMR** ($CDCl_3$, 150 MHz) δ 175.3, 168.3, 153.2, 135.9, 129.5, 128.9, 127.1, 126.7 (q, J_{CF3} = 274.3 Hz), 66.0, 55.4, 46.4, 42.8, 38.0, 37.2, 35.7, 31.3 (q, J_{CF3} = 28.8 Hz), 26.3, 25.4, 24.4, 24.0.

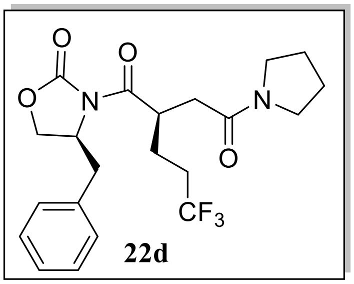

22d

(R)-1-((S)-4-Benzyl-2-oxooxazolidin-3-yl)-4-(pyrrolidin-1-yl)-2-(3,3,3-trifluoropropyl)butane-1,4-dione

The reaction was performed following the procedure described for **22a** but pyrrolidine was used. The residue was purified by column chromatography on silica gel (EtOAc/DCM, 5:95 to 15:85) to afford **22d** (99.5 mg, 55%) as a clear oil. **^{1}H-NMR** ($CDCl_3$, 600 MHz) δ 7.36-7.25 (m, 5H), 4.68 (td, J = 6.8, 3.3 Hz, 1H), 4.34 (td, J = 10.3, 6.3 Hz, 1H), 4.20-4.16 (m, 2H), 3.48-3.38 (m, 5H), 2.94-2.87 (m, 1H), 2.76 (dd, J = 13.3, 10.8 Hz, 1H), 2.45 (dd, J = 16.4, 3.6 Hz, 1H), 2.20 (dt, J = 27.8, 9.8 Hz, 2H), 1.99-1.91 (m, 3H), 1.87-1.75 (m, 3H); **^{13}C-NMR** ($CDCl_3$, 150 MHz) δ 175.3, 168.5, 153.2, 135.9, 129.4, 128.9, 127.1, 126.7 (q, J_{CF3} = 275.7 Hz), 66.0, 55.5, 46.5, 45.7, 37.8, 37.3, 37.0, 31.3 (q, J_{CF3} = 28.8 Hz), 26.0, 24.3, 24.1.

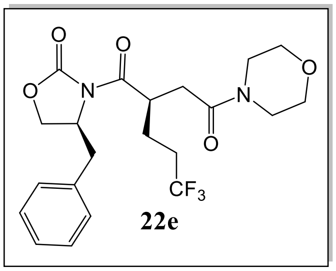

22e

(R)-1-((S)-4-Benzyl-2-oxooxazolidin-3-yl)-4-morpholino-2-(3,3,3-trifluoropropyl)butane-1,4-dione

The reaction was performed following the procedure described for **22a** but morpholine was used. The residue was purified by column chromatography on silica gel (EtOAc/DCM, 5:95 to

15:85) to afford **22e** (108.5 mg, 58%) as a clear oil. **^{1}H-NMR** ($CDCl_3$, 600 MHz) δ 7.33 (t, J = 7.6 Hz, 2H), 7.27 (d, J = 6.5 Hz, 3H), 4.68 (t, J = 8.4 Hz, 1H), 4.35-4.32 (m, 1H), 4.22-4.17 (m, 2H), 3.73-3.46 (m, 8H), 3.36 (d, J = 13.4 Hz, 1H), 2.97 (dd, J = 16.2, 10.7 Hz, 1H), 2.76 (dd, J = 13.3, 10.5 Hz, 1H), 2.49 (dd, J = 16.2, 3.8 Hz, 1H), 2.20 (td, J = 19.1, 9.8 Hz, 2H), 1.94 (td, J = 14.6, 7.6 Hz, 1H), 1.79 (td, J = 14.8, 7.5 Hz, 1H); **^{13}C-NMR** ($CDCl_3$, 150 MHz) δ 175.0, 168.9, 153.2, 135.7, 129.5, 128.9, 127.1, 126.6 (q, J_{CF3} = 274.3 Hz), 66.7, 66.4, 66.0, 55.4, 45.7, 41.9, 37.9, 37.2, 35.4, 31.3 (q, J_{CF3} = 28.8 Hz), 24.0.

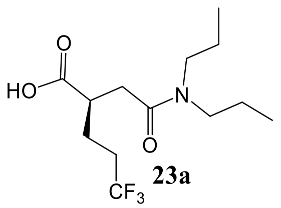

23a

(R)-2-(2-(Dipropylamino)-2-oxoethyl)-5,5,5-trifluoropentanoic acid

To solution of **22a** (20 mg, 0.044 mmol) in THF (3 mL) and water (1 mL) at 0 °C were added 30% aqueous solution of H_2O_2 (30 µL, 0.18 mmol) and solution of LiOH (5 mg) in water (1 mL). After 10 minutes, mixture was allowed to warm to room temperature. After 1 h, the reaction was allowed to cool to 0 °C and saturated $NaHCO_3$ (5 mL) and Na_2SO_3 (5 mL) were added. After stirring 15 minutes, mixture was concentrated (removing THF) and the aqueous layer was washed with DCM (10 mL). The aqueous layer was acidified to pH 3 with 1M HCl. The acidified aqueous layer was extracted with EtOAc (2 x 10 mL) and the combined organic extracts were washed with brine, dried over anhydrous Na_2SO_4, filtered and concentrated to afford **23a** (13.5 mg), which was used in the next step without purification. **^{1}H-NMR** ($CDCl_3$, 600 MHz) δ 3.32-3.26 (m, 2H), 3.26-3.17 (m, 2H), 2.99-2.94 (m, 1H), 2.75 (dd, J = 16.5, 9.3 Hz, 1H), 2.55 (dd,

J = 16.9, 3.8 Hz, 1H), 2.36-2.27 (m, 1H), 2.22-2.14 (m, 1H), 2.01 (tt, J = 14.4, 5.3 Hz, 1H), 1.75-1.70 (m, 1H), 1.67-1.53 (m, 4H), 1.30-1.22 (m, 1H), 0.95 (t, J = 7.4 Hz, 3H), 0.84-0.90 (m, 3H); **^{13}C-NMR** ($CDCl_3$, 150 MHz) δ 176.3, 171.3, 126.8 (q, J_{CF3} = 275.8 Hz), 49.9, 48.3, 40.2, 35.1, 32.0, 31.7 (q, J_{CF3} = 28.8 Hz), 24.2, 21.9, 20.7, 11.3, 11.2.

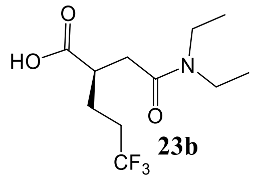

23b

(R)-2-(2-(Diethylamino)-2-oxoethyl)-5,5,5-trifluoropentanoic acid

The reaction was performed following the procedure described for **23a** and **22b** was used as starting material and crude **23b** (101.7 mg, 45%) was afforded. **23b** was used for the next step without purification. **^{1}H-NMR** ($CDCl_3$, 600 MHz) δ 3.44-3.29 (m, 4H), 3.01-2.97 (m, 1H), 2.78 (dd, J = 16.5, 9.0 Hz, 1H), 2.50 (dd, J = 16.4, 4.3 Hz, 1H), 2.35-2.14 (m, 2H), 2.00-1.93 (m, 1H), 1.80 -1. 74 (m, 1H), 1.21 (t, J = 7.1 Hz, 3H), 1.12 (t, J = 7.1 Hz, 3H); **^{13}C-NMR** ($CDCl_3$, 150 MHz) δ 177.2, 170.5, 126.8 (q, J_{CF3} = 274.3 Hz), 42.3, 40.8, 40.3, 35.0, 32.0, 31.7 (q, J_{CF3} = 28.6 Hz), 24.2, 14.0, 12.8.

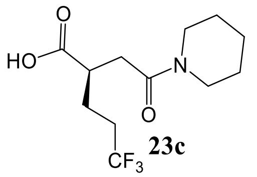

23c

(R)-5,5,5-Trifluoro-2-(2-oxo-2-(piperidin-1-yl)ethyl)pentanoic acid

The reaction was performed following the procedure described for **23a** and **22c** was used as starting material and crude **23c** (61.3 mg, 51%) was afforded. **23c** was used for the next step

without purification. **^{1}H-NMR** ($CDCl_3$, 600 MHz) δ 3.59-3.52 (m, 2H), 3.42 (t, J = 5.2 Hz, 2H), 2.98-2.95 (m, 1H), 2.78 (dd, J = 16.5, 9.0 Hz, 1H), 2.50 (dd, J = 16.5, 4.1 Hz, 1H), 2.35-2.14 (m, 2H), 1.99-1.93 (m, 1H), 1.80-1.74 (m, 1H), 1.63 (dq, J = 36.6, 5.6 Hz, 4H), 1.54 (d, J = 23.1 Hz, 2H); **^{13}C-NMR** ($CDCl_3$, 150 MHz) δ 177.4, 169.4, 126.8 (q, J_{CF3} = 275.8 Hz), 60.4, 46.7, 43.2, 40.2, 35.2, 31.7 (q, J_{CF3} = 28.6 Hz), 26.2, 25.4, 24.3, 24.2, 14.2.

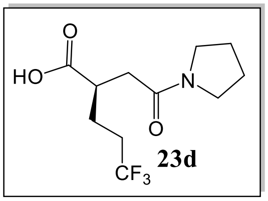

23d

(R)-5,5,5-Trifluoro-2-(2-oxo-2-(pyrrolidin-1-yl)ethyl)pentanoic acid

The reaction was performed following the procedure described for **23a** and **22d** was used as starting material and crude **23d** (60.8 mg, 51%) was afforded. **23d** was used for the next step without purification. **^{1}H-NMR** ($CDCl_3$, 600 MHz) δ 3.52-3.40 (m, 4H), 3.00-2.95 (m, 1H), 2.73 (dd, J = 16.5, 9.3 Hz, 1H), 2.49-2.43 (m, 1H), 2.35-2.15 (m, 2H), 2.02-1.85 (m, 5H), 1.81-1.75 (m, 1H); **^{13}C-NMR** ($CDCl_3$, 150 MHz) δ 176.6, 170.1, 126.8 (q, J_{CF3} = 275.7 Hz), 46.9, 46.2, 39.9, 36.4, 31.6 (q, J_{CF3} = 28.6 Hz), 25.9, 24.3, 24.2.

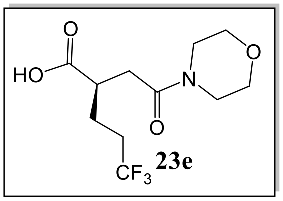

23e

(R)-5,5,5-Trifluoro-2-(2-morpholino-2-oxoethyl)pentanoic acid

The reaction was performed following the procedure described for **23a** and **22e** was used as starting material and crude **23e** (65 mg, 53%) was afforded. **23e** was used for the next step without purification. **^{1}H-NMR** ($CDCl_3$, 600 MHz) δ 3.70 -3. 47 (m, 8H), 2.99 (d, J = 4.1 Hz, 1H), 2.80 (dd, J = 16.2, 9.0 Hz, 1H), 2.46 (dd, J = 16.5, 3.8 Hz, 1H), 2.33-2.14 (m, 2H), 1.97 -1.89 (m, 1H), 1.83-1.79 (m, 1H); **^{13}C-NMR** ($CDCl_3$, 150 MHz) δ 178.0, 169.5, 126.7 (q, J_{CF3} = 274.3 Hz), 66.7, 66.3, 45.8, 42.2, 40.0, 34.9, 31.7 (q, J_{CF3} = 28.6 Hz), 24.2.

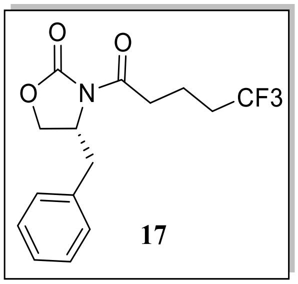

17

(R)-4-Benzyl-3-(5,5,5-trifluoropentanoyl)oxazolidin-2-one

The reaction was performed following the procedure described for **14**. 5,5,5-trifluoropentanoic acid (1 g, 5.65 mmol) and (*R*)-4-benzyloxazolidin-2-one were used. The residue was purified by column chromatography on silica gel (EtOAc/hexane, 15:85 to 30:70) to afford **17** (1.49 g, 78%) as a yellow oil. **^{1}H-NMR** ($CDCl_3$, 600 MHz) δ 7.34 (t, J = 7.2 Hz, 2H), 7.30-7.26 (m, 1H), 7.21 (d, J = 7.6 Hz, 2H), 4.70-4.67 (m, 1H), 4.24-4.19 (m, 2H), 3.30 (dd, J = 13.4, 3.1 Hz, 1H), 3.04 (qt, J = 18.0, 7.2 Hz, 2H), 2.78 (dd, J = 13.1, 9.6 Hz, 1H), 2.25-2.16 (m, 2H), 2.00-1.90 (m, 2H); **^{13}C-NMR** ($CDCl_3$, 150 MHz) δ 171.9, 153.4, 135.0, 129.3, 129.0, 127.4, 126.8 (q, J_{CF3} = 273.0 Hz), 66.3, 55.1, 37.9, 34.2, 32.9 (q, J_{CF3} = 28.8 Hz), 16.6.

Note, the reaction was repeated with yields of 84–94%.

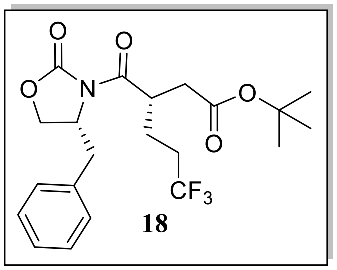

(S)-tert-Butyl 3-((R)-4-benzyl-2-oxooxazolidine-3-carbonyl)-6,6,6-trifluorohexanoate

The reaction was performed following the procedure described for **15.** The compound **17** (2.53 g, 8.02 mmol) and 1M LiHMDS were used. The residue was purified by column chromatography on silica gel (EtOAc/hexane, 15:85 to 30:70) to afford **18** (2.24 g, 65%) as a yellow oil. **^{1}H-NMR** ($CDCl_3$, 600 MHz) δ 7.35 (t, J = 7.4 Hz, 2H), 7.29 (d, J = 7.6 Hz, 1H), 7.26 (d, J = 5.5 Hz, 2H), 4.70-4.66 (m, 1H), 4.22-4.17 (m, 3H), 3.35-3.32 (dd, J = 13.4, 3.1 Hz, 1H), 2.84 (dd, J = 16.7, 9.8 Hz, 1H), 2.74 (dd, J = 13.3, 10.2 Hz, 1H), 2.46 (dd, J = 16.7, 4.6 Hz, 1H), 2.20-2.12 (m, 2H), 1.99-1.93 (m, 1H), 1.80-1.75 (m, 1H), 1.43 (s, 9H); **^{13}C-NMR** ($CDCl_3$, 150 MHz) δ 174.45, 170.57, 153.09, 135.47, 129.46, 128.98, 128.4 (q, J_{CF3} = 274.9 Hz), 127.32, 81.2, 66.1, 55.4, 38.2, 37.4, 37.0, 31.2 (q, J_{CF3} = 28.6 Hz), 28.0, 23.7.

Note, the reaction was repeated with yields of 33–65%.

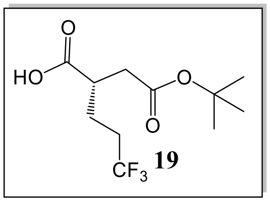

(S)-2-(2-(tert-Butoxy)-2-oxoethyl)-5,5,5-trifluoropentanoic acid

The reaction was performed following the procedure described for **23a** and **18** (1.1 g, 2.56 mmol) was used and product **19** (552 mg, 79%) was used for the next step without purification. **^{1}H-NMR** ($CDCl_3$, 600 MHz) δ 2.90-2.85 (m, 1H), 2.67 (q, J = 8.3 Hz, 1H), 2.44 (dd, J = 16.4, 5.7

Hz, 1H), 2.27-2.14 (m, 2H), 1.97-1.90 (m, 1H), 1.85-1.80 (m, 1H), 1.44 (s, 9H); **^{13}C-NMR** ($CDCl_3$, 150 MHz) δ 179.8, 170.4, 126.7 (q, J_{CF3} = 274.3 Hz), 81.7, 40.1, 37.0, 31.5 (q, J_{CF3} = 28.6 Hz), 28.0, 23.6.

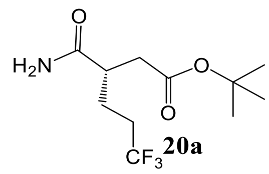

20a

(S)-tert-Butyl 3-carbamoyl-6,6,6-trifluorohexanoate

The reaction was performed following the procedure described for **6**. **19** (75 mg, 0.28 mmol) and ammonium chloride (10 mmol) were used and the product was purified by column chromatography on silica gel (EtOAc/DCM, 5:95 to 15:85) to afford **20a** (63 mg, 85%) as a light yellow solid. **^{1}H-NMR** ($CDCl_3$, 600 MHz) δ 6.14 (s, 1H), 5.95 (s, 1H), 2.73 (q, J = 4.5 Hz, 1H), 2.66 (dd, J = 16.7, 9.5 Hz, 1H), 2.35 (dd, J = 16.7, 4.0 Hz, 1H), 2.22-2.09 (m, 2H), 1.96-1.89 (m, 1H), 1.72-1.66 (m, 1H), 1.44 (s, 9H); **^{13}C-NMR** ($CDCl_3$, 150 MHz) δ 175.9, 171.4, 126.8 (q, J_{CF3} = 274.3 Hz), 81.5, 40.6, 38.0, 31.3 (q, J_{CF3} = 28.8 Hz), 28.0, 24.3.

Note, the reaction was repeated with yields of 43–85%.

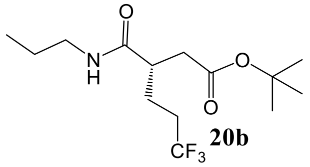

20b

(S)-tert-Butyl 6,6,6-trifluoro-3-(propylcarbamoyl)hexanoate

The reaction was performed following the procedure described for **6**. **19** (50 mg, 0.18 mmol) and propylamine (25 µL, 0.28 mmol) were used and the product was purified by column

chromatography on silica gel (EtOAc/DCM, 5:95 to 15:85) to afford **20b** (35 mg, 32%) as a light-yellow solid. **^{1}H-NMR** ($CDCl_3$, 600 MHz) δ 6.04 (s, 1H), 3.23-3.19 (m, 2H), 2.66-2.61 (m, 2H), 2.35-2.32 (m, 1H), 2.17-2.06 (m, 2H), 1.96-1.92 (m, 1H), 1.66-1.64 (m, 1H), 1.54-1.49 (m, 2H), 1.43 (d, J = 9.3 Hz, 9H), 0.93-0.90 (m, 3H); **^{13}C-NMR** ($CDCl_3$, 150 MHz) δ 173.1, 171.5, 126.9 (q, J_{CF3} = 274.3 Hz), 81.3, 41.4, 41.2, 38.3, 31.4 (q, J_{CF3} = 28.8 Hz), 28.0, 24.4, 22.8, 11.3.

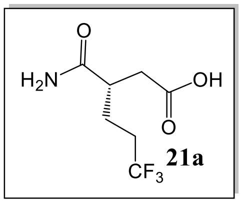

21a

(S)-3-Carbamoyl-6,6,6-trifluorohexanoic acid

The reaction was performed following the procedure described for **22**. **20a** (127 mg, 0.47 mmol) was used and product **21a** was still impure after attempted purification by column chromatography. Therefore, it was used for the next step without further purification. **^{1}H-NMR** (DMSO, 600 MHz) δ 12.47-11.85 (s, 1H), 7.45 (s, 1H), 6.95 (s, 1H), 2.62 (dd, J = 13.9, 7.5 Hz, 1H), 2.48 (d, J = 8.6 Hz, 1H), 2.30 (dd, J = 16.5, 5.9 Hz, 1H), 2.23-2.15 (m, 2H), 1.66-1.59 (m, 2H).

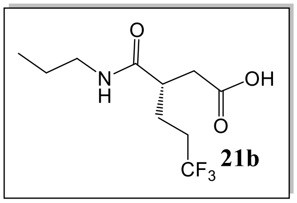

21b

(S)-6,6,6-Trifluoro-3-(propylcarbamoyl)hexanoic acid

The reaction was performed following the procedure described for **22**. **20b** (35 mg, 0.12 mmol) was used and product **21b** (31 mg) was used for the next step without purification. **^{1}H-NMR** ($CDCl_3$, 600 MHz) δ 6.27 (s, 1H), 3.24-3.20 (m, 2H), 2.77 (dd, J = 17.0, 9.1 Hz, 1H), 2.66 (t, J = 4.3 Hz, 1H), 2.49 (dd, J = 17.0, 3.6 Hz, 1H), 2.17-2.06 (m, 2H), 1.97-1.91 (m, 1H), 1.76-1.70 (m, 1H), 1.52 (td, J = 14.4, 7.1 Hz, 2H), 0.92 (q, J = 7.6 Hz, 3H); **^{13}C-NMR** ($CDCl_3$, 150 MHz) δ 175.4, 173.8, 126.7 (q, J_{CF3} = 274.3 Hz), 41.5, 41.3, 36.5, 34.9, 31.2 (q, J_{CF3} = 28.6 Hz), 24.5, 22.6, 11.2.

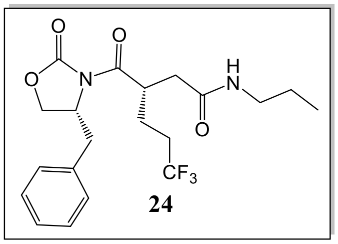

24

(S)-3-((R)-4-Benzyl-2-oxooxazolidine-3-carbonyl)-6,6,6-trifluoro-N-propylhexanamide

The reaction was performed following the procedure described for **22**. **18** (910 mg, 2.12 mmol) and the previously described carboxylic acid intermediate (200 mg, 0.47 mmol) were used. The resulting product was then used without purification to couple with propylamine. The product the amide was purified by column chromatography on silica gel (EtOAc/DCM, 5:95 to 10:90) to afford **24** (110 mg, 57%) as a light-yellow oil and white solids. **^{1}H-NMR** ($CDCl_3$, 600 MHz) δ 7.34 (t, J = 7.4 Hz, 2H), 7.29-7.25 (m, 1H), 7.23 (d, J = 7.2 Hz, 2H), 5.82 (s, 1H), 4.82-4.68 (m, 1H), 4.25-4.20 (m, 2H), 4.13-4.08 (m, 1H), 3.34 (dd, J = 13.4, 3.1 Hz, 1H), 3.20 (tt, J = 20.6, 6.7 Hz, 2H), 2.80-2.72 (m, 2H), 2.33 (dd, J = 14.6, 6.4 Hz, 1H), 2.23-2.11 (m, 2H), 2.05-1.99 (m, 1H), 1.85-1.79 (m, 1H), 1.51 (td, J = 14.5, 7.2 Hz, 2H), 0.91 (t, J = 7.4 Hz, 3H); **^{13}C-NMR** ($CDCl_3$, 150

MHz) δ 174.1, 169.9, 153.3, 135.3, 129.4, 128.9, 127.3, 126.7 (q, J_{CF3} = 274.3 Hz), 66.3, 55.4, 41.3, 39.4, 38.5, 37.5, 31.3 (q, J_{CF3} = 28.8 Hz), 23.5, 22.8, 11.3.

Note, the reaction was repeated with yields of 57–85%.

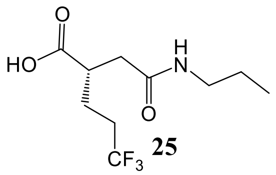

25

(S)-5,5,5-Trifluoro-2-(2-oxo-2-(propylamino)ethyl)pentanoic acid

The reaction was performed following the procedure described for **23a. 24** (110 mg, 0.27 mmol) was used to produce **25** (27.2 mg), which was used for the next step without purification. **^{1}H-NMR** ($CDCl_3$, 600 MHz) δ 6.17 (s, 1H), 3.24-3.16 (m, 2H), 2.93-2.88 (m, 1H), 2.67-2.61 (m, 1H), 2.48-2.41 (m, 1H), 2.31-2.06 (m, 2H), 1.98-1.88 (m, 1H), 1.83-1.71 (m, 1H), 1.58-1.50 (m, 2H), 0.93-0.84 (m, 3H); **^{13}C-NMR** ($CDCl_3$, 150 MHz) δ 177.1, 171.5, 127.2 (q, J_{CF3} = 274.2 Hz), 41.7, 40.4, 37.5, 31.5 (q, J_{CF3} = 30.1 Hz), 23.9, 22.5, 11.2.

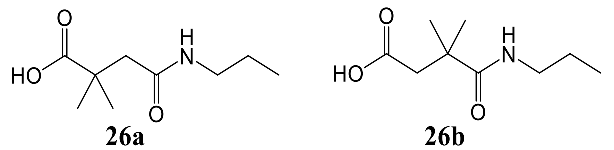

26a **26b**

26a : 2,2-dimethyl-4-oxo-4-(propylamino)butanoic acid

26b : 3,3-dimethyl-4-oxo-4-(propylamino)butanoic acid

2,2-Dimethylsuccinic anhydride (300 mg, 2.34 mmol) and propylamine (380 µL, 4.68 mmol) were dissolved in anhydrous THF (4 mL) and maintained at room temperature for overnight. After completion of the reaction, the mixture was concentrated and the residue was purified by

column chromatography on silica gel (MeOH/DCM, 5:95 to 15:85) to afford product (**26a** : 70 mg, **26b** : 20 mg) as white solids. A sample was purified using Method B. HPLC purity via Method B: ≥ 95%, **26a**: t_R = 16 min. **26b**: t_R = 15 min.

26a ^{1}H-NMR ($CDCl_3$, 600 MHz) 6.15 (t, J = 4.8 Hz, 1H), 3.25 (q, J = 6.7 Hz, 2H), 2.64 (s, 2H), 1.55 (td, J = 14.6, 7.2 Hz, 2H), 1.32 (s, 6H), 0.93 (t, J = 7.4 Hz, 3H); **26a ^{13}C-NMR** ($CDCl_3$, 150 MHz) 178.9, 173.8, 45.5, 41.7, 40.5, 26.1, 22.5, 11.2.

26b ^{1}H-NMR ($CDCl_3$, 600 MHz) 6.51 (t, J = 4.2 Hz, 1H), 3.24 (q, J = 6.8 Hz, 2H), 2.52 (s, 2H), 1.54 (td, J = 14.6, 7.5 Hz, 2H), 1.29 (s, 6H), 0.92 (t, J = 7.4 Hz, 3H); **26b ^{13}C-NMR** ($CDCl_3$, 150 MHz) 180.2, 172.3, 44.9, 41.6, 41.2, 25.7, 22.5, 11.3.

Note, each product was confirmed by HMBC analysis.

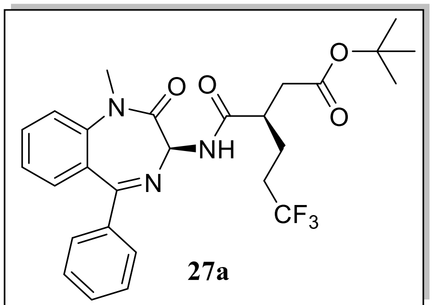

27a

(R)-tert-Butyl-6,6,6-trifluoro-3-(((S)-1-methyl-2-oxo-5-phenyl-2,3-dihydro-1H-benzo[e][1,4]diazepin-3-yl)carbamoyl)hexanoate

To a solution of **8** (21.8 mg, 0.082 mmol) and **16** (20 mg, 0.074 mmol) in DMF (2 mL) were added TBTU (28.9 mg, 0.09 mmol) and triethylamine (17 µL, 0.12 mmol). The reaction was stirred at room temperature for overnight. After completion of the reaction, the mixture was diluted with water (10 mL). The aqueous layer was washed with EtOAc (2 × 10 mL), and the combined organic extracts were washed with brine, dried over anhydrous Na_2SO_4, filtered and concentrated.

The residue was purified by column chromatography on silica gel (EtOAc/DCM, 5:95 to 15:85) to afford **27a** (32 mg, 78%) as a white solid. **^{1}H-NMR** ($CDCl_3$, 600 MHz) δ 7.59 (t, J = 8.6 Hz, 3H), 7.48-7.35 (m, 6H), 7.23 (t, J = 7.6 Hz, 1H), 5.51 (d, J = 7.9 Hz, 1H), 3.47 (s, 3H), 2.87 (d, J = 14.1 Hz, 1H), 2.74 (q, J = 8.0 Hz, 1H), 2.41 (dd, J = 16.5, 6.2 Hz, 1n), 2.27-2.21 (m, 2H), 1.99-1.93 (m, 1H), 1.81-1.76 (m, 1H), 1.48 (s, 9H); **^{13}C-NMR** ($CDCl_3$, 150 MHz) δ 173.2, 170.6, 167.6, 167.4, 142.8, 137.8, 131.9, 130.7, 130.5, 129.7, 128.9, 128.2, 126.9 (q, J_{CF3} = 275.7 Hz), 124.5, 121.7, 81.2, 67.6, 41.7, 38.2, 35.4, 31.3 (q, J_{CF3} = 28.8 Hz), 28.0, 24.4.

Note, the reaction was repeated with yields of 63–78%. Instead of TBTU, EDC•HCl, HOBt hydrate and TEA also used for other trials.

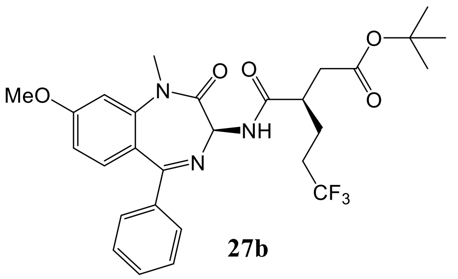

27b

(R)-tert-Butyl 6,6,6-trifluoro-3-(((S)-8-methoxy-1-methyl-2-oxo-5-phenyl-2,3-dihydro-1H-benzo[e][1,4]diazepin-3-yl)carbamoyl)hexanoate

The reaction was performed following the procedure described for **6**. **9** (40 mg, 0.135 mmol) was used. The residue was purified by column chromatography on silica gel (MeOH/DCM, 2:98 to 5:95) to afford product **27b** (60 mg, 81%) as a white solid. **^{1}H-NMR** ($CDCl_3$, 600 MHz) δ 7.59 (d, J = 7.6 Hz, 2H), 7.46 (t, J = 7.2 Hz, 1H), 7.40-7.36 (m, 3H), 7.25 (d, J = 8.3 Hz, 1H), 6.84 (d, J = 2.1 Hz, 1H), 6.77 (dd, J = 9.0, 2.1 Hz, 1H), 5.51 (d, J = 7.9 Hz, 1H), 3.90 (s, 3H), 3.45 (s, 3H), 2.87 (s, 1H), 2.73 (q, J = 8.1 Hz, 1H), 2.41 (dd, J = 16.4, 6.4 Hz, 1H), 2.25 (qd, J = 10.2, 5.9 Hz,

2H), 1.97-1.94 (m, 1H), 1.80-1.77 (m, 1H), 1.44-1.48 (m, 9n); **^{13}C-NMR** ($CDCl_3$, 150 MHz) δ 173.2, 170.6, 167.4, 167.3, 162.1, 144.5, 138.1, 132.1, 130.6, 129.8, 128.1, 126.9 (q, J_{CF3} = 274.3 Hz), 122.0, 110.6, 106.7, 81.2, 67.7, 55.7, 41.7, 38.1, 35.5, 31.3 (q, J_{CF3} = 27.3 Hz), 28.0, 24.4.24.3.

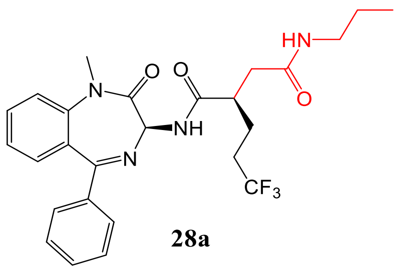

28a

(R)-N1-((S)-1-Methyl-2-oxo-5-phenyl-2,3-dihydro-1H-benzo[e][1,4]diazepin-3-yl)-N4-propyl-2-(3,3,3-trifluoropropyl)succinamide

To a solution of **27a** (30 mg, 0.058 mmol) in DCM (1 mL) at 0 °C was added TFA (1 mL). The reaction was stirred at 0 °C for 1 h and at room temperature for 2.5 h. After completion of the reaction, the mixture was diluted with DCM (3 x 5 mL) several times and concentrated to afford carboxylic acid intermediate. Then, it was used for the next step without purification. To a solution of carboxylic acid intermediate (30 mg, 0.065 mmol) in THF (3 mL) at 0 °C were added triethylamine (30 μL, 0.20 mmol) and disuccinimidyl carbonate (33 mg, 0.13 mmol). After 15 minutes, the mixture was allowed warm to room temperature and stirred for 3 h. The precipitated solids were removed by filtration and then propylamine (~ 10 μL, 0.13 mmol) was added to the filtrate and stirred 6 h at room temperature. After completion of the reaction, the mixture was concentrated and the residue was purified by column chromatography on silica gel (EtOAc/DCM, 5:95 to 15:85) to afford **28a** (12 mg, 38%) as a white solid. **^{1}H-NMR** ($CDCl_3$, 600 MHz) δ 7.74 (d, J = 7.6 Hz, 1H), 7.64-7.57 (m, 3H), 7.50 (t, J = 7.4 Hz, 1H), 7.41 (q, J = 7.7 Hz, 3H), 7.35 (d, J = 7.9 Hz, 1H), 7.26 (d, J = 2.8 Hz, 1H), 6.13 (s, 1H), 5.48 (d, J = 7.6 Hz, 1H), 3.48 (s, 3H), 3.21

(dq, J = 13.1, 3.6 Hz, 2H), 3.02-2.97 (m, 1H), 2.66 (dd, J = 14.5, 9.0 Hz, 1H), 2.41 (dd, J = 14.5, 5.2 Hz, 1H), 2.30-2.21 (m, 2H), 1.99-1.93 (m, 1H), 1.80 (dq, J = 19.1, 5.4 Hz, 1H), 1.50 (td, J = 14.4, 7.1 Hz, 2H), 0.87 (t, J = 7.4 Hz, 3H); ^{13}**C-NMR** ($CDCl_3$, 150 MHz) δ 173.9, 171.0, 168.3, 167.2, 142.9, 137.1, 132.4, 131.2, 130.7, 129.9, 128.5, 128.3, 126.8 (q, J_{CF3} = 274.3 Hz), 124.6, 121.8, 67.6, 42.4, 41.5, 39.0, 35.5, 31.3 (q, J_{CF3} = 28.8 Hz), 24.7, 22.6, 11.3; A sample for bioassay assessment was purified using Method B. HPLC purity via Method B: ≥ 95%, t_R = 22.6 min. HRMS (ESI): m/z [M + H]+ calculated for $C_{26}H_{29}F_3N_4O_3H$: 503.2270; found: 503.2265.

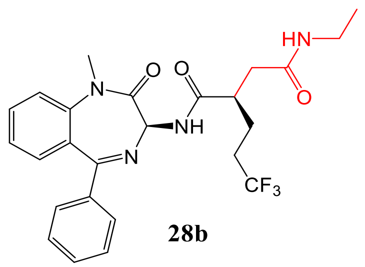

28b

((R)-N4-Ethyl-N1-((S)-1-methyl-2-oxo-5-phenyl-2,3-dihydro-1H-benzo[e][1,4]diazepin-3-yl)-2-(3,3,3-trifluoropropyl)succinimide

The reaction was performed using 20 mg of carboxylic acid intermediate from **27a** and ethylamine by following the procedure described for **28a**. The residue was purified by column chromatography on silica gel (EtOAc/DCM, 5:95 to 15:85) to afford **28b** (21 mg, 80%) as a white solid. ^{1}**H-NMR** ($CDCl_3$, 600 MHz) δ 7.73 (d, J = 7.6 Hz, 1H), 7.65-7.62 (m, 1H), 7.58 (d, J = 7.6 Hz, 2H), 7.50 (t, J = 7.4 Hz, 1H), 7.43-7.35 (m, 4H), 7.26 (t, J = 7.6 Hz, 1H), 6.08 (s, 1H), 5.48 (d, J = 7.6 Hz, 1H), 3.48 (s, 3H), 3.30 (td, J = 12.8, 7.1 Hz, 2H), 2.98 (td, J = 9.3, 4.7 Hz, 1H), 2.65 (dd, J = 14.5, 9.0 Hz, 1H), 2.40 (dd, J = 14.5, 5.2 Hz, 1H), 2.29-2.22 (m, 2H), 2.00-1.93 (m, 1H), 1.84-1.79 (m, 1H), 1.12 (t, J = 7.4 Hz, 3H); ^{13}**C-NMR** ($CDCl_3$, 150 MHz) δ 173.9, 170.8, 168.4, 167.2, 142.9, 137.1, 132.4, 131.6, 131.3, 130.8, 130.6, 129.9, 128.5, 128.3, 126.8 (q, J_{CF3} = 275.7

Hz), 124.6, 121.8, 67.6, 42.4, 39.0, 35.6, 34.7, 31.4 (q, J_{CF3} = 28.6 Hz), 24.7, 14.5; A sample for bioassay assessment was purified using Method B. HPLC purity via Method B: ≥ 95%, t_R = 21.8 min. HRMS (ESI): m/z [M + H]+ calculated for $C_{25}H_{27}F_3N_4O_3H$: 489.2110; found: 489.2108.

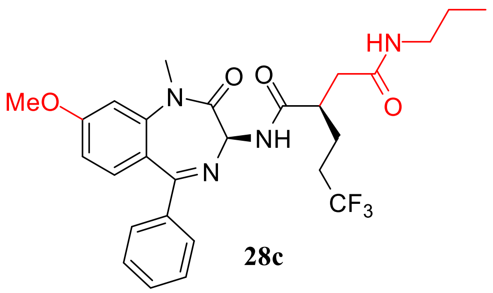

28c

(R)-N1-((S)-8-Methoxy-1-methyl-2-oxo-5-phenyl-2,3-dihydro-1H-benzo[e][1,4]diazepin-3-yl)-N4-propyl-2-(3,3,3-trifluoropropyl)succinimide

The reaction was performed following the procedure described for **28a** using **27b** (60 mg, 0.11 mmol) to give carboxylic acid intermediate, which was used for the next step without purification. The reaction was performed using 60 mg of carboxylic acid intermediate by following the procedure described for **28a**. The residue was purified by column chromatography on silica gel (MeOH/DCM, 2:98 to 5:95) to afford **28c** (42 mg, 61%) as a white solid. **^{1}H-NMR** ($CDCl_3$, 600 MHz) δ 8.32 (d, J = 7.2 Hz, 1H), 7.60 (q, J = 7.0 Hz, 3H), 7.47 (t, J = 7.7 Hz, 2H), 7.31 (d, J = 9.0 Hz, 1H), 6.91 (d, J = 1.7 Hz, 1H), 6.86 (dd, J = 9.0, 2.1 Hz, 1H), 6.41 (t, J = 5.4 Hz, 1n), 5.61 (d, J = 7.6 Hz, 1H), 3.95 (s, J = 6.9 Hz, 3H), 3.51 (s, 3H), 3.24-3.20 (m, 2H), 3.01-2.98 (m, 1H), 2.75 (dd, J = 14.5, 8.3 Hz, 1H), 2.43 (dd, J = 14.5, 5.5 Hz, 1H), 2.25-2.20 (m, 2H), 2.01-1.97 (m, 1H), 1.85-1.81 (m, 1H), 1.54-1.49 (m, 2H), 0.89 (t, J = 7.4 Hz, 3H); **^{13}C-NMR** ($CDCl_3$, 150 MHz) δ 173.8, 170.7, 168.4, 167.0, 162.7, 144.8, 137.1, 132.6, 131.3, 130.1, 128.3, 126.8 (q, J_{CF3} = 274.2 Hz), 121.4, 110.9, 106.9, 67.4, 55.8, 42.4, 41.4, 39.2, 35.7, 31.4 (q, J_{CF3} = 28.8 Hz), 24.7, 22.7,

11.3; A sample for bioassay assessment was purified using Method B. HPLC purity via Method B: ≥ 95%, t_R = 23.0 min. HRMS (ESI): m/z [M + Na]+ calculated for $C_{27}H_{31}F_3N_4O_4Na$: 555.2190; found: 555.2190.

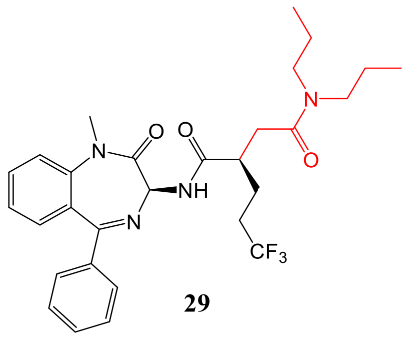

29

(R)-N1-((S)-1-Methyl-2-oxo-5-phenyl-2,3-dihydro-1H-benzo[e][1,4]diazepin-3-yl)-N4,N4-dipropyl-2-(3,3,3-trifluoropropyl)succinamide

To solution of **8** (10.5 mg, 0.040 mmol) in DCM (4 mL) was added **23a** (13 mg, 0.044 mmol), and EDC·HCl (11 mg, 0.06 mmol), HOBt hydrate (10 mg, 0.06 mmol) and triethylamine (10 µL, 0.06 mmol). The reaction was stirred at room temperature for overnight. After completion of the reaction, the mixture was concentrated and the residue was purified by column chromatography on silica gel (EtOAc/DCM, 5:95 to 15:85) to afford **29**(17 mg, 78%) as a white solid. **^{1}H-NMR** ($CDCl_3$, 600 MHz) δ 7.76 (d, J = 7.6 Hz, 1H), 7.60 (q, J = 7.7 Hz, 3H), 7.48 (t, J = 7.2 Hz, 1H), 7.41-7.38 (m, 3H), 7.34 (d, J = 7.9 Hz, 1H), 7.23 (t, J = 7.6 Hz, 1H), 5.47 (d, J = 7.6 Hz, 1H), 3.47 (s, 3H), 3.34-3.23 (m, 3H), 3.19-3.09 (m, 2H), 2.86 (q, J = 8.1 Hz, 1H), 2.45 (dd, J = 16.2, 5.2 Hz, 1H), 2.35-2.22 (m, 2H), 2.00-1.94 (m, 1H), 1.81-1.75 (m, 1H), 1.64-1.52 (m, 4H), 0.93 (t, J = 7.4 Hz, 3H), 0.88 (t, J = 7.4 Hz, 3H); **^{13}C-NMR** ($CDCl_3$, 150 MHz) δ 174.2, 170.4, 168.0, 167.3, 143.0, 137.4, 132.2, 131.0, 130.6, 129.9, 128.7, 128.2, 126.9 (q, J_{CF3} = 274.2 Hz),

124.4, 121.7, 67.7, 49.8, 48.1, 41.7, 35.8, 35.5, 31.6 (q, J_{CF3} = 28.6 Hz), 24.8, 22.1, 20.8, 11.3, 11.2; A sample for bioassay assessment was purified using Method B. HPLC purity via Method B: ≥ 95%, t_R = 25.6 min. HRMS (ESI): m/z [M + H]+ calculated for $C_{29}H_{35}F_3N_4O_3H$: 545.2735; found: 545.2734.

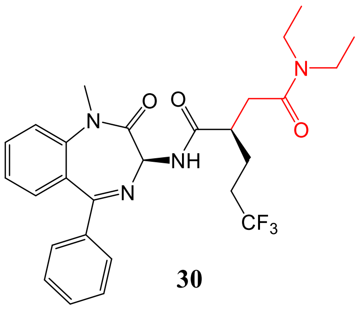

30

(R)-N4,N4-Diethyl-N1-((S)-1-methyl-2-oxo-5-phenyl-2,3-dihydro-1H-benzo[e][1,4]diazepin-3-yl)-2-(3,3,3-trifluoropropyl)succinamide

The reaction was performed following the procedure described for **29** using amine **8** (20 mg, 0.08 mmol) and carboxylic acid **23b**. The residue was purified by column chromatography on silica gel (MeOH/DCM, 5:95 to 10:90) to afford **30** (15 mg, 36%) as a white solid. **^{1}H-NMR** ($CDCl_3$, 600 MHz) δ 7.59 (dd, J = 16.2, 7.6 Hz, 4H), 7.46 (t, J = 7.4 Hz, 1H), 7.39-7.33 (m, 4H), 7.22 (t, J = 7.6 Hz, 1H), 5.47 (d, J = 7.6 Hz, 1H), 3.46 (s, 3H), 3.41-3.29 (m, 4H), 3.11 (s, 1H), 2.84 (q, J = 8.1 Hz, 1H), 2.43 (dd, J = 16.2, 5.2 Hz, 1H), 2.35-2.25 (m, 2H), 1.98 (d, J = 10.3 Hz, 1H), 1.80 (d, J = 12.7 Hz, 1H), 1.19 (t, J = 7.1 Hz, 3H), 1.12 (t, J = 6.9 Hz, 3H); **^{13}C-NMR** ($CDCl_3$, 150 MHz) δ 174.3, 170.0, 168.3, 167.3, 142.9, 137.3, 132.3, 131.1, 130.7, 129.9, 128.6, 128.2, 126.9 (q, J_{CF3} = 274.3 Hz), 124.5, 121.7, 67.6, 42.2, 41.6, 40.7, 35.7, 35.5, 31.5 (q, J_{CF3} = 28.8 Hz), 24.8, 14.1, 12.9; A sample for bioassay assessment was purified using Method B. HPLC

purity via Method B: ≥ 95%, t_R = 23.7 min. HRMS (ESI): m/z [M + H]+ calculated for $C_{27}H_{31}F_3N_4O_3H$: 517.2420; found: 517.2421.

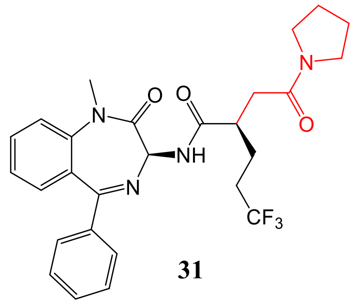

31

(R)-5,5,5-Trifluoro-N-((S)-1-methyl-2-oxo-5-phenyl-2,3-dihydro-1H-benzo[e][1,4]diazepin-3-yl)-2-(2-oxo-2-(pyrrolidin-1-yl)ethyl)pentanamide

The reaction was performed following the procedure described for **29** using amine **8** (15 mg, 0.057 mmol) and carboxylic acid **23d**. The residue was purified by column chromatography on silica gel (MeOH/DCM, 5:95 to 10:90) to afford **31** (27 mg, 92%) as a white solid. **^{1}H-NMR** ($CDCl_3$, 600 MHz) δ 7.95 (d, J = 7.2 Hz, 1H), 7.64-7.57 (m, 3H), 7.50 (t, J = 7.4 Hz, 1H), 7.42-7.39 (m, 3H), 7.34 (dd, J = 7.7, 1.5 Hz, 1H), 7.25-7.23 (m, 1H), 5.48 (d, J = 7.2 Hz, 1H), 3.53-3.46 (m, 6H), 3.45-3.41 (m, 1H), 3.12-3.08 (m, 1H), 2.84 (dd, J = 16.2, 9.0 Hz, 1H), 2.44 (dd, J = 16.2, 4.8 Hz, 1H), 2.36-2.23 (m, 2H), 2.02-1.93 (m, 3H), 1.88-1.77 (m, 3H); **^{13}C-NMR** ($CDCl_3$, 150 MHz) δ 174.2, 169.7, 168.6, 167.2, 143.0, 137.1, 132.4, 131.2, 130.9, 130.0, 128.5, 128.3, 126.9 (q, J_{CF3} = 274.3 Hz), 124.6, 121.7, 67.6, 46.9, 46.1, 41.3, 37.2, 35.5, 31.5 (q, J_{CF3} = 28.8 Hz), 25.9, 24.8, 24.3; A sample for bioassay assessment was purified using Method B. HPLC purity via Method B: ≥ 95%, t_R = 23.0 min. HRMS (ESI): m/z [M + H]+ calculated for $C_{27}H_{29}F_3N_4O_3H$: 515.2264; found: 515.2265.

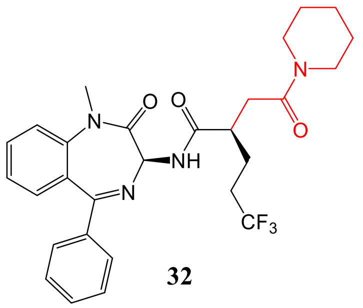

32

(R)-5,5,5-Trifluoro-N-((S)-1-methyl-2-oxo-5-phenyl-2,3-dihydro-1H-benzo[e][1,4]diazepin-3-yl)-2-(2-oxo-2-(piperidin-1-yl)ethyl)pentanamide

The reaction was performed following the procedure described for **29** using amine **8** (13.6 mg, 0.051 mmol) and carboxylic acid **23c**. The residue was purified by column chromatography on silica gel (MeOH/DCM, 5:95 to 10:90) to afford **32** (25 mg, 85%) as a white solid. ^{1}**H-NMR** ($CDCl_3$, 600 MHz) δ 7.85 (d, J = 7.2 Hz, 1H), 7.63-7.58 (m, 3H), 7.49 (t, J = 7.4 Hz, 1H), 7.42-7.39 (m, 3H), 7.33 (dd, J = 7.9, 1.4 Hz, 1H), 7.26-7.23 (m, 1H), 5.49 (d, J = 7.6 Hz, 1H), 3.64-3.60 (m, 1H), 3.55-3.51 (m, 1H), 3.48 (s, 3H), 3.46-3.40 (m, 2H), 3.10-3.05 (m, 1H), 2.90 (dd, J = 16.4, 8.8 Hz, 1H), 2.46 (dd, J = 16.2, 4.8 Hz, 1H), 2.35-2.24 (m, 2H), 1.99-1.93 (m, 1H), 1.83-1.77 (m, 1H), 1.67-1.54 (m, 6H); ^{13}**C-NMR** ($CDCl_3$, 150 MHz) δ 174.4, 169.0, 168.5, 167.3, 143.0, 137.2, 132.4, 131.2, 130.8, 129.9, 128.6, 128.3, 126.9 (q, J_{CF3} = 274.3 Hz), 124.5, 121.7, 67.6, 46.7, 43.2, 41.6, 35.8, 35.5, 31.5 (q, J_{CF3} = 30.1 Hz), 26.3, 25.4, 24.9, 24.3; A sample for bioassay assessment was purified using Method B. HPLC purity via Method B: ≥ 95%, t_R = 24.1 min. HRMS (ESI): m/z [M + H]+ calculated for $C_{28}H_{31}F_3N_4O_3H$: 529.2420; found: 529.2421.

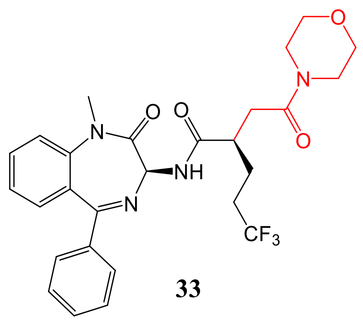

33

(R)-5,5,5-Trifluoro-N-((S)-1-methyl-2-oxo-5-phenyl-2,3-dihydro-1H-benzo[e][1,4]diazepin-3-yl)-2-(2-morpholino-2-oxoethyl)pentanamide

The reaction was performed following the procedure described for **29** using amine **8** (13 mg, 0.051 mmol) and carboxylic acid **23e**. The residue was purified by column chromatography on silica gel (MeOH/DCM, 5:95 to 10:90) to afford **33** (24 mg, 80%) as a white solid. **^{1}H-NMR** ($CDCl_3$, 600 MHz) δ 7.68 (d, J = 7.2 Hz, 1H), 7.62-7.58 (m, 3H), 7.48 (t, J = 7.4 Hz, 1H), 7.41-7.38 (m, 3H), 7.33 (dd, J = 7.9, 1.4 Hz, 1H), 7.25-7.22 (m, 1H), 5.48 (d, J = 7.6 Hz, 1H), 3.71-3.60 (m, 6H), 3.53-3.45 (m, 5H), 3.11-3.06 (m, 1H), 2.89 (q, J = 8.3 Hz, 1H), 2.41 (dd, J = 16.2, 4.8 Hz, 1H), 2.34-2.26 (m, 2H), 2.00-1.93 (m, 1H), 1.84-1.80 (m, 1H); **^{13}C-NMR** ($CDCl_3$, 150 MHz) δ 174.0, 169.3, 168.1, 167.4, 142.9, 137.5, 132.1, 131.0, 130.6, 129.8, 128.8, 128.3, 126.9 (q, J_{CF3} = 274.2 Hz), 124.5, 121.7, 67.7, 66.8, 66.4, 45.8, 42.1, 41.5, 35.5, 35.5, 31.4 (q, J_{CF3} = 28.8 Hz), 25.0; A sample for bioassay assessment was purified using Method B. HPLC purity via Method B: ≥ 95%, t_R = 22.0 min. HRMS (ESI): m/z [M + H]+ calculated for $C_{27}H_{29}F_3N_4O_4H$: 531.2216; found: 531.2214.

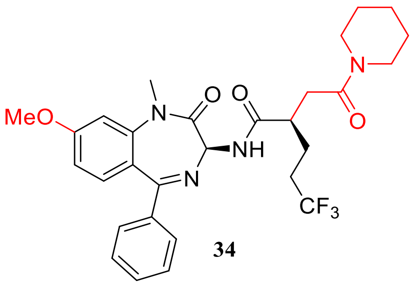

34

(R)-5,5,5-Trifluoro-N-((S)-8-methoxy-1-methyl-2-oxo-5-phenyl-2,3-dihydro-1H-benzo[e][1,4]diazepin-3-yl)-2-(2-oxo-2-(piperidin-1-yl)ethyl)pentanamide

The reaction was performed following the procedure described for **29** using amine **9** (15.9 mg, 0.054 mmol) and carboxylic acid **23c** (16.8 mg, 0.06 mmol). The residue was purified by column chromatography on silica gel (MeOH/DCM, 5:95 to 10:90) to afford **34** (22 mg, 72%) as a white solid. **^{1}H-NMR** ($CDCl_3$, 600 MHz) δ 7.60 (d, J = 7.6 Hz, 2H), 7.53 (d, J = 7.2 Hz, 1H), 7.45 (t, J = 7.4 Hz, 1H), 7.38 (t, J = 7.6 Hz, 2H), 7.24 (d, J = 8.6 Hz, 1H), 6.84 (d, J = 2.1 Hz, 1H), 6.75 (dd, J = 8.6, 2.1 Hz, 1H), 5.48 (d, J = 7.2 Hz, 1H), 3.90 (s, 3H), 3.61-3.52 (m, 2H), 3.45-3.43 (m, 3H), 3.41-3.38 (m, 2H), 3.09-3.05 (m, 1H), 2.85 (q, J = 8.1 Hz, 1H), 2.43 (dd, J = 16.2, 5.2 Hz, 1H), 2.38-2.25 (m, 2H), 2.00-1.94 (m, 1H), 1.84-1.81 (m, 1H), 1.66-1.52 (m, 6H); **^{13}C-NMR** ($CDCl_3$, 150 MHz) δ 174.1, 168.6, 167.4, 167.3, 162.0, 144.6, 138.1, 132.0, 130.6, 129.8, 128.1, 127.0 (q, J_{CF3} = 275.7 Hz), 122.1, 110.6, 106.6, 68.1, 55.7, 46.4, 42.9, 41.6, 35.9, 35.5, 31.5 (q, J_{CF3} = 28.65 Hz), 26.3, 25.5, 24.9, 24.5; A sample for bioassay assessment was purified using Method B. HPLC purity via Method B: ≥ 95%, t_R = 23.8 min. HRMS (ESI): m/z [M + H]+ calculated for $C_{29}H_{33}F_3N_4O_4H$: 559.2527; found: 559.256.

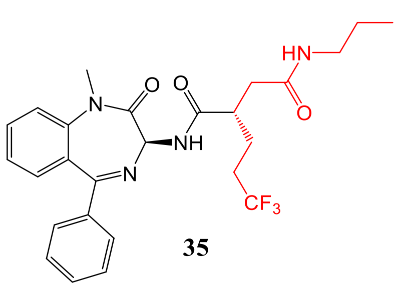

35

(S)-N1-((S)-1-Methyl-2-oxo-5-phenyl-2,3-dihydro-1H-benzo[e][1,4]diazepin-3-yl)-N4-propyl-2-(3,3,3-trifluoropropyl)succinimide

The reaction was performed following the procedure described for **29** using amine **8** (20 mg, 0.08 mmol) and carboxylic acid **25** (25 mg, 0.098 mmol). The residue was purified by column chromatography on silica gel (MeOH/DCM, 5:95 to 10:90) to afford **35** (25 mg, 62%) as a white solid. **^{1}H-NMR** ($CDCl_3$, 600 MHz) 7.85 (d, J = 6.5 Hz, 1H), 7.63-7.57 (m, 3H), 7.49 (t, J = 7.4 Hz, 1H), 7.41 (t, J = 7.7 Hz, 3H), 7.38 (dd, J = 7.9, 1.4 Hz, 1H), 7.26 (t, J = 7.2 Hz, 1H), 6.16 (t, J = 4.2 Hz, 1H), 5.47 (d, J = 7.2 Hz, 1H), 3.46 (s, 3H), 3.25-3.14 (m, 2H), 2.99 (t, J = 4.3 Hz, 1H), 2.66 (dd, J = 14.6, 8.8 Hz, 1H), 2.40 (dd, J = 14.8, 4.5 Hz, 1H), 2.30-2.18 (m, 2H), 2.02-1.97 (m, 1H), 1.84-1.79 (m, 1H), 1.48 (td, J = 14.5, 7.3 Hz, 2H), 0.87 (t, J = 7.2 Hz, 3H); **^{13}C-NMR** ($CDCl_3$, 150 MHz) 173.9, 171.1, 168.1, 167.5, 142.7, 137.3, 132.2, 131.1, 130.7, 129.8, 128.7, 128.3, 126.8 (q, J_{CF3} = 274.3 Hz), 124.7, 121.7, 67.8, 41.9, 41.5, 39.0, 35.5, 31.4 (q, J_{CF3} = 28.6 Hz), 24.5, 22.5, 11.2; A sample for bioassay assessment was purified using Method B. HPLC purity via Method B: ≥ 95%, t_R = 23.2 min. HRMS (ESI): m/z [M + H]+ calculated for $C_{26}H_{29}F_3N_4O_3H$: 503.2265; found: 503.2265.

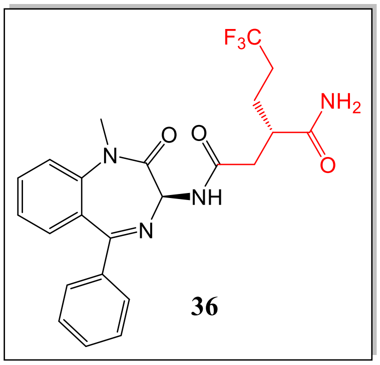

36

(S)-N1-((S)-1-Methyl-2-oxo-5-phenyl-2,3-dihydro-1H-benzo[e][1,4]diazepin-3-yl)-3-(3,3,3-trifluoropropyl)succinimide

The reaction was performed following the procedure described for **27a** using amine **8** (56 mg, 0.21 mmol) and carboxylic acid **21a** (45 mg, 0.21 mmol). The residue was purified by column chromatography on silica gel (MeOH/DCM, 5:95 to 15:85) to afford **36** (50 mg, 51%) as a white solid. **^{1}H-NMR** ($CDCl_3$, 600 MHz) δ 7.87 (d, J = 6.5 Hz, 1H), 7.65-7.62 (m, 1H), 7.57 (d, J = 7.6 Hz, 2H), 7.50 (t, J = 7.4 Hz, 1H), 7.42-7.36 (m, 4H), 7.28 (d, J = 7.9 Hz, 1H), 6.63 (s, 1H), 6.38 (s, 1H), 5.47 (d, J = 7.6 Hz, 1H), 3.47 (s, 3H), 2.89-2.86 (m, 1H), 2.76 (dd, J = 15.7, 9.8 Hz, 1H), 2.52 (dd, J = 15.5, 3.1 Hz, 1H), 2.19-2.10 (m, 2H), 1.98-1.95 (m, 1H), 1.73 (dt, J = 13.4, 5.3 Hz, 1H); **^{13}C-NMR** ($CDCl_3$, 150 MHz) δ 177.3, 171.2, 168.7, 167.2, 142.7, 137.2, 132.6, 131.3, 130.9, 129.9, 128.5, 128.4, 126.7 (q, J_{CF3} = 275.7 Hz), 124.9, 121.7, 67.2, 40.8, 38.7, 35.6, 31.4 (q, J_{CF3} = 28.6 Hz), 24.3; A sample for bioassay assessment was purified using Method B. HPLC purity via Method B: ≥ 94%, t_R = 20.4 min. HRMS (ESI): m/z [M + H]+ calculated for $C_{23}H_{23}F_3N_4O_3$: 461.1799; found: 461.1795.

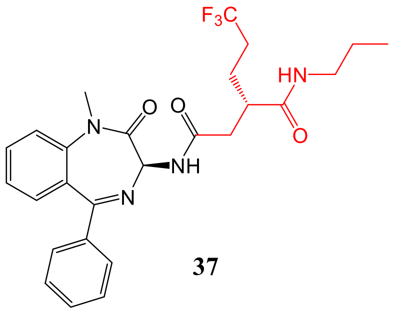

37

(S)-N4-((S)-1-Methyl-2-oxo-5-phenyl-2,3-dihydro-1H-benzo[e][1,4]diazepin-3-yl)-N1-propyl-2-(3,3,3-trifluoropropyl)succinamide

The reaction was performed following the procedure described for **29** using amine **8** (15 mg, 0.057 mmol) and carboxylic acid **21b** (21 mg, 0.084 mmol). The residue was purified by column chromatography on silica gel (MeOH/DCM, 5:95 to 15:85) to afford **37** (16 mg, 56%) as a white solid. ^{1}**H-NMR** ($CDCl_3$, 600 MHz) δ 7.68 (d, J = 7.6 Hz, 1H), 7.65-7.62 (m, 1H), 7.59-7.56 (m, 2H), 7.51-7.49 (m, 1H), 7.42-7.37 (m, 4H), 7.29-7.27 (m, 1H), 6.34 (t, J = 5.5 Hz, 1H), 5.45 (d, J = 7.6 Hz, 1H), 3.48 (s, 3H), 3.23 (td, J = 13.4, 7.2 Hz, 1H), 3.12 (ddd, J = 20.3, 7.2, 5.7 Hz, 1H), 2.81-2.75 (m, 2H), 2.53 (dd, J = 19.6, 8.3 Hz, 1H), 2.20-2.06 (m, 2H), 2.01 (qd, J = 9.2, 4.6 Hz, 1H), 1.76-1.71 (m, 1H), 1.46 (td, J = 14.6, 7.2 Hz, 2H), 0.85 (t, J = 7.4 Hz, 3H); ^{13}**C-NMR** ($CDCl_3$, 150 MHz) δ 173.6, 171.4, 168.5, 167.1, 142.7, 137.3, 132.4, 131.2, 131.0, 129.9, 128.6, 128.4, 126.8 (q, J_{CF3} = 274.3 Hz), 124.8, 121.6, 67.1, 41.6, 41.3, 38.9, 35.5, 31.5 (q, J_{CF3} = 28.6 Hz), 24.5, 22.6, 11.2; A sample for bioassay assessment was purified using Method B. HPLC purity via Method B: ≥ 95%, t_R = 22.6 min. HRMS (ESI): m/z [M + H]+ calculated for $C_{26}H_{29}F_3N_4O_3H$: 503.2271; found: 503.2265.

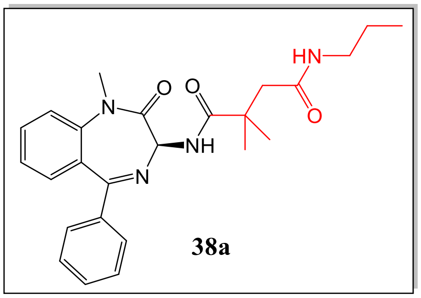

(S)-2,2-Dimethyl-N1-(1-methyl-2-oxo-5-phenyl-2,3-dihydro-1H-benzo[e][1,4]diazepin-3-yl)-N4-propylsuccinamide

The reaction was performed following the procedure described for **29** using amine **8** (10 mg, 0.037 mmol) and 2,2-dimethyl-4-oxo-4-(propylamino)butanoic acid (**26a**, 8.5 mg, 0.045 mmol). The residue was purified by column chromatography on silica gel (MeOH/DCM, 5:95 to 15:85) to afford **38a** (9 mg, 56%) as a white solid. **^{1}H-NMR** ($CDCl_3$, 600 MHz) 7.64 (d, J = 7.2 Hz, 1H), 7.60 (d, J = 7.9 Hz, 3H), 7.48 (t, J = 7.4 Hz, 1H), 7.41-7.36 (m, 4H), 7.24 (t, J = 7.8 Hz, 1H), 6.39 (t, J = 4.2 Hz, 1H), 5.47 (d, J = 7.2 Hz, 1H), 3.48 (s, 3H), 3.21-3.12 (m, 2H), 2.54 (dd, J = 45.1, 13.8 Hz, 2H), 1.48 (td, J = 14.6, 7.3 Hz, 2H), 1.43 (s, 3H), 1.40 (s, 3H), 0.85 (t, J = 7.2 Hz, 3H); **^{13}C-NMR** ($CDCl_3$, 150 MHz) 177.8, 170.6, 167.8, 167.6, 142.7, 137.9, 132.0, 130.8, 130.5, 129.7, 129.0, 128.2, 124.5, 121.6, 67.9, 47.1, 41.9, 41.1, 35.4, 26.2, 25.6, 22.8, 11.4; A sample for bioassay assessment was purified using Method B. HPLC purity via Method B: ≥ 95%, t_R = 21.2 min. HRMS (ESI): m/z $[M + H]^+$ calculated for $C_{25}H_{30}N_4O_3H$: 435.2391; found: 435.2391.

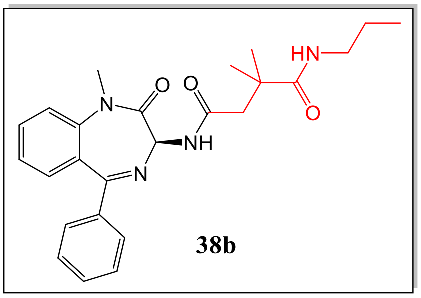

(S)-2,2-Dimethyl-N4-(1-methyl-2-oxo-5-phenyl-2,3-dihydro-1H-benzo[e][1,4]diazepin-3-yl)-N1-propylsuccinamide

The reaction was performed following the procedure described for **29** using amine **8** (10 mg, 0.037 mmol) and 3,3-dimethyl-4-oxo-4-(propylamino)butanoic acid (**26b**, 8.5 mg, 0.045 mmol). The residue was purified by column chromatography on silica gel (MeOH/DCM, 5:95 to 15:85) to afford **38b** (8 mg, 50%) as a white solid. **^{1}H-NMR** ($CDCl_3$, 600 MHz) δ 7.63-7.61 (m, 1H), 7.59 (t, J = 6.9 Hz, 3H), 7.48 (t, J = 7.2 Hz, 1H), 7.40 (t, J = 7.2 Hz, 3H), 7.35 (dd, J = 7.7, 1.2 Hz, 1H), 7.24 (t, J = 7.6 Hz, 1H), 6.74 (t, J = 6.1 Hz, 1H), 5.48 (d, J = 7.9 Hz, 1H), 3.46 (s, 3H), 3.21 (qd, J = 13.1, 5.9 Hz, 2H), 2.73 (d, J = 14.5 Hz, 1H), 2.63 (d, J = 14.5 Hz, 1H), 1.51 (td, J = 14.6, 7.3 Hz, 2H), 1.36-1.32 (m, 6H), 0.88 (t, J = 7.4 Hz, 3H); **^{13}C-NMR** ($CDCl_3$, 150 MHz); 177.4, 171.6, 167.8, 167.5, 142.8, 137.7, 132.0, 130.9, 130.7, 129.8, 128.8, 128.2, 124.5, 121.6, 67.3, 46.2, 41.5, 41.4, 35.4, 26.5, 25.9, 22.6, 11.3. A sample for bioassay assessment was purified using Method B. HPLC purity via Method B: ≥ 95%, t_R = 21.1 min. HRMS (ESI): m/z [M + H]$^+$ calculated for $C_{25}H_{30}N_4O_3H$: 435.2394; found: 435.2391.

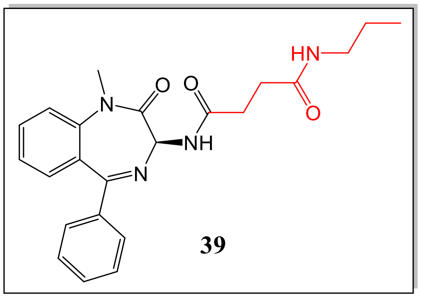

39

(S)-N1-(1-Methyl-2-oxo-5-phenyl-2,3-dihydro-1H-benzo[e][1,4]diazepin-3-yl)-N4-propylsuccinamide

The reaction was performed following the procedure described for **29** using amine **8** (15 mg, 0.057 mmol) and 4-oxo-4-(propylamino)butanoic acid (10.8 mg, 0.068 mmol). The residue

was purified by column chromatography on silica gel (MeOH/DCM, 5:95 to 15:85) to afford **39** (18 mg, 78%) as a white solid. **^{1}H-NMR** ($CDCl_3$, 600 MHz) δ 7.59 (t, J = 6.7 Hz, 3H), 7.50-7.45 (m, 2H), 7.40-7.35 (m, 4H), 7.24 (t, J = 7.6 Hz, 1H), 6.07 (s, 1H), 5.50 (d, J = 7.6 Hz, 1H), 3.47 (s, 3H), 3.19 (dq, J = 23.8, 6.4 Hz, 2H), 2.75 (t, J = 6.4 Hz, 2H), 2.57-2.51 (m, 2H), 1.49 (td, J = 14.6, 7.3 Hz, 2H), 0.89 (t, J = 7.4 Hz, 3H); **^{13}C-NMR** ($CDCl_3$, 150 MHz) δ 172.2, 171.8, 167.6, 167.5, 142.7, 138.0, 131.9, 130.7, 130.6, 129.7, 129.0, 128.2, 124.5, 121.5, 67.2, 41.3, 35.4, 31.8, 31.6, 22.8, 11.3; A sample for bioassay assessment was purified using Method B. HPLC purity via Method B: ≥ 95%, t_R = 19.6 min. HRMS (ESI): m/z $[M + H]^+$ calculated for $C_{23}H_{26}N_4O_3H$: 407.2080; found: 407.2078.

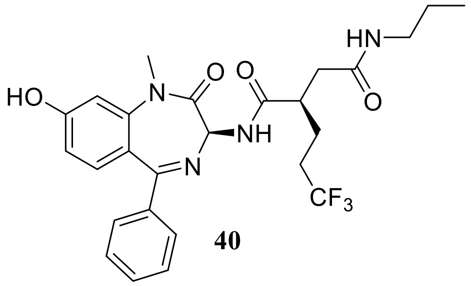

40

(R)-N1-((S)-8-hydroxy-1-methyl-2-oxo-5-phenyl-2,3-dihydro-1H-benzo[e][1,4]diazepin-3-yl)-N4-propyl-2-(3,3,3-trifluoropropyl)succinimide

To a solution of **28c** (10 mg, 0.018 mmol) in DCM (2 mL) was added BBr_3 (112 µL, 0.112 mmol) dropwise at 0 °C. The reaction was maintained at room temperature for overnight. Additional BBr_3 (540 µL, 0.54 mmol) was divided into four portions and added over 3 days. After completion of the reaction, the mixture was quenched by saturated aqueous Na_2CO_3. The aqueous layer was washed with EtOAc (2 × 10 mL) and the combined organic extracts were washed with brine, dried over anhydrous Na_2SO_4, filtered and concentrated. The residue was purified by column

chromatography on silica gel (MeOH/DCM, 2:98 to 5:95) to afford **40** (7 mg) with an estimated purity of 70%, which was used for the next step without further purification. **^{1}H-NMR** ($CDCl_3$, 600 MHz) δ 7.67 (d, J = 6.5 Hz, 1H), 7.57-7.54 (m, 3H), 7.45 (t, J = 7.2 Hz, 1H), 7.36 (t, J = 7.2 Hz, 2H), 7.00 (d, J = 8.6 Hz, 1H), 6.75 (s, 1H), 6.54 (d, J = 8.6 Hz, 1H), 5.85 (s, 1H), 5.35 (d, J = 6.5 Hz, 1H), 3.38 (s, 3H), 3.22-3.17 (m, 2H), 3.05 (s, 1H), 2.68-2.62 (m, 1H), 2.41-2.33 (m, 3H), 2.03-1.99 (m, 1H), 1.85 (m, 1H), 1.51-1.47 (m, 2H), 0.89-0.84 (m, 3H).

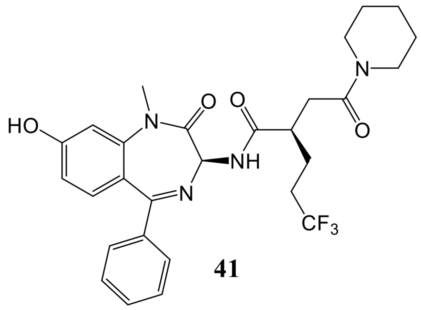

41

(R)-5,5,5-trifluoro-N-((S)-8-hydroxy-1-methyl-2-oxo-5-phenyl-2,3-dihydro-1H-benzo[e][1,4]diazepin-3-yl)-2-(2-oxo-2-(piperidin-1-yl)ethyl)pentanamide

The reaction was performed following the procedure described for **40** using **34** (80 mg, 0.143 mmol). The residue was purified by column chromatography on silica gel (MeOH/DCM, 2:98 to 5:95) to afford **41** (65 mg) with an estimated purity of 80%, which was used for the next step without further purification. **^{1}H-NMR** ($CDCl_3$, 600 MHz) δ 7.62 (d, J = 6.2 Hz, 1H), 7.57 (d, J = 7.6 Hz, 2H), 7.43 (t, J = 6.9 Hz, 1H), 7.35 (t, J = 7.2 Hz, 3H), 6.94 (d, J = 8.6 Hz, 1H), 6.70 (s, 1H), 6.48 (d, J = 7.6 Hz, 1H), 5.31 (d, J = 6.2 Hz, 1H), 3.52 (d, J = 6.2 Hz, 2H), 3.41 (d, J = 1.7 Hz, 2H), 3.37 (s, 3H), 3.14-3.07 (m, 1H), 2.94-2.87 (m, 1H), 2.48-2.34 (m, 3H), 2.06-2.01 (m, 1H), 1.91-1.82 (m, 1H), 1.62-1.44 (s, 6H).

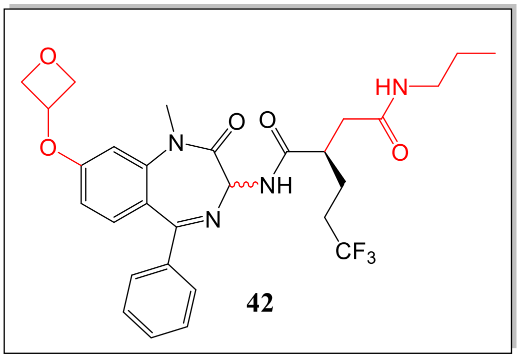

42

(R)-N1-((S)-1-methyl-8-(oxetan-3-yloxy)-2-oxo-5-phenyl-2,3-dihydro-1H-benzo[e][1,4]diazepin-3-yl)-N4-propyl-2-(3,3,3-trifluoropropyl)succinimide

To a solution of **40** (22 mg, 0.042 mmol) in DMF (2 mL) was added 3-bromooxetane (6.3 µL, 0.084 mmol) and K_2CO_3 (11 mg, 0.084 mmol). The reaction was stirred at 100 °C for overnight. After completion of the reaction, the mixture was diluted with water (10 mL). The aqueous layer was washed with EtOAc (2 × 20 mL) and the combined organic extracts were washed with additional water (20 mL), brine, dried over anhydrous Na_2SO_4, filtered, and concentrated. The residue was purified by column chromatography on silica gel (MeOH/DCM, 5:95 to 10:90) to afford **42** (13 mg, 53%) as a white solid. Note, epimerization at carbon 3 occurred under the reaction conditions. The two diastereomers were separated by HPLC using Method B. The configurations at carbon 3 have not been assigned. NMRs were written based on retention times. **42-Diastereomer A**, HPLC purity via Method B: 87%, t_R = 22.5 min. HRMS (ESI): m/z [M + Na]$^+$ calculated for $C_{29}H_{33}F_3N_4O_5Na$: 597.2293; found: 597.2292. **^{1}H-NMR** ($CDCl_3$, 600 MHz) δ 7.91 (d, J = 6.2 Hz, 1H), 7.55 (dd, J = 22.9, 7.1 Hz, 3H), 7.43 (d, J = 7.2 Hz, 2H), 7.28 (s, 1H), 6.74 (s, 1H), 6.55 (d, J = 8.6 Hz, 1H), 6.14 (s, 1H), 5.52 (d, J = 7.6 Hz, 1H), 5.31 (d, J = 5.2 Hz, 1H), 5.03 (d, J = 6.5 Hz, 2H), 4.82 (s, 2H), 3.46 (s, 3H), 3.22 (m, 2H), 2.98 (d, J = 3.4 Hz, 1H), 2.70-2.67 (m, 1H), 2.41 (d, J = 14.1 Hz, 1H), 2.25 (s, 2H), 1.97 (d, J = 4.1 Hz, 1H), 1.82 (d, J = 3.1 Hz, 1H), 1.51 (q, J = 6.6 Hz, 2H), 0.89-0.84 (t, J = 6.6 Hz, 3H); **^{13}C-NMR** ($CDCl_3$, 150 MHz)

δ 174.0, 171.0, 168.9, 166.7, 159.9, 145.1, 136.4, 133.4, 131.8, 130.3, 128.4, 126.8 (q, J_{CF3} = 274.2 Hz), 121.8, 110.8, 108.0, 77.5, 77.3, 70.7, 67.1, 42.3, 41.5, 39.0, 35.8, 31.4 (q, J_{CF3} = 30.1 Hz), 24.6, 22.6, 11.3. **42-Diastereomer B**, HPLC purity via Method B: 88%, t_R = 22.9 min. HRMS (ESI): m/z $[M + Na]^+$ calculated for $C_{29}H_{33}F_3N_4O_5Na$: 597.2294; found: 597.2293. **^{1}H-NMR** ($CDCl_3$, 600 MHz) δ 7.88 (s, 1H), 7.57 (d, J = 6.9 Hz, 2H), 7.50 (s, 1H), 7.42 (d, J = 6.5 Hz, 2H), 7.29 (d, J = 8.6 Hz, 1H), 6.72 (s, 1H), 6.53 (d, J = 8.6 Hz, 1H), 6.10 (s, 1H), 5.49 (d, J = 6.2 Hz, 1H), 5.30 (s, 1H), 5.03 (d, J = 6.2 Hz, 2H), 4.81 (s, 2H), 3.43 (s, 3H), 3.23-3.16 (m, 2H), 2.98 (m, 1H), 2.68-2.64 (m, 1H), 2.39 (d, J = 14.5 Hz, 1H), 2.28-2.18 (m, 2H), 2.05-2.00 (m, 1H), 1.81 (m, 1H), 1.50 (q, J = 6.6 Hz, 2H), 0.93-0.84 (t, J = 6.6 Hz, 3H); **^{13}C-NMR** ($CDCl_3$, 150 MHz) δ 174.0, 170.9, 168.1, 167.2, 159.5, 144.8, 137.1, 132.9, 131.3, 130.0, 128.4, 126.8 (q, J_{CF3} = 274.3 Hz), 122.4, 110.6, 107.8, 77.5, 77.4, 70.7, 67.6, 41.9, 41.5, 39.0, 35.6, 31.51 (q, J_{CF3} = 28.6 Hz), 24.4, 22.5, 11.3.

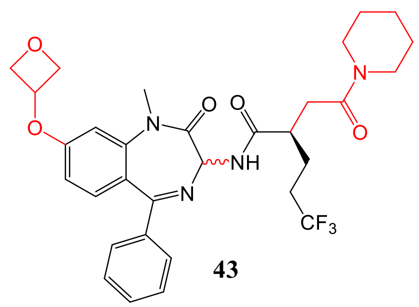

43

(R)-5,5,5-trifluoro-N-((S)-1-methyl-8-(oxetan-3-yloxy)-2-oxo-5-phenyl-2,3-dihydro-1H-benzo[e][1,4]diazepin-3-yl)-2-(2-oxo-2-(piperidin-1-yl)ethyl)pentanamide

The reaction was performed following the procedure described for **42** using **41** (40 mg, 0.073 mmol). The residue was purified by column chromatography on silica gel (MeOH/DCM, 2:98 to 5:90) to afford **43** (33 mg, 75%) as a white solid. Note, epimerization at carbon 3 occurred

under the reaction conditions. The two diastereomers were separated by HPLC using Method B. The configurations at carbon 3 have not been assigned. NMRs were written based on retention times. **43-Diastereomer A**, HPLC purity via Method B: 90%, t_R = 23.5 min. HRMS (ESI): m/z $[M + Na]^+$ calculated for $C_{31}H_{35}F_3N_4O_5Na$: 623.2451; found: 623.2450. **^{1}H-NMR** ($CDCl_3$, 600 MHz) δ 7.59 (d, J = 7.6 Hz, 2H), 7.53 (d, J = 7.2 Hz, 1H), 7.46 (t, J = 6.9 Hz, 1H), 7.38 (t, J = 7.6 Hz, 2H), 7.24 (d, J = 8.6 Hz, 1H), 6.69 (s, 1H), 6.50 (d, J = 8.6 Hz, 1H), 5.48 (d, J = 7.2 Hz, 1H), 5.29 (t, J = 5.3 Hz, 1H), 5.02 (q, J = 6.5 Hz, 2H), 4.82-4.80 (m, 2H), 3.60-3.53 (m, 2H), 3.43-3.41 (m, 5H), 3.07 (s, 1H), 2.85 (q, J = 8.1 Hz, 1H), 2.43 (dd, J = 16.4, 4.3 Hz, 1H), 2.38-2.23 (m, 2H), 1.97 (dd, J = 23.8, 14.1 Hz, 1H), 1.83-1.79 (m, 1H), 1.64-1.54 (m, 6H); **^{13}C-NMR** ($CDCl_3$, 150 MHz) δ 174.1, 168.6, 167.3, 167.1, 159.0, 144.8, 137.9, 132.3, 130.7, 129.8, 128.2, 127.0 (q, J_{CF3} = 276 Hz), 122.9, 110.4, 107.6, 77.6, 77.4, 70.5, 68.1, 46.4, 42.9, 41.5, 35.9, 35.5, 31.5 (J_{CF3} = 28.5 Hz), 26.3, 25.5, 24.9, 24.4. **43-Diastereomer B**, HPLC purity via Method B: 90%, t_R = 24.0 min. HRMS (ESI): m/z $[M + Na]^+$ calculated for $C_{31}H_{35}F_3N_4O_5Na$: 623.2451; found: 623.2450. **^{1}H-NMR** ($CDCl_3$, 600 MHz) δ 7.84 (d, J = 7.2 Hz, 1H), 7.57 (d, J = 7.6 Hz, 2H), 7.45 (t, J = 7.4 Hz, 1H), 7.38 (t, J = 7.4 Hz, 2H), 7.27 (s, 1H), 6.67 (s, 1H), 6.49 (d, J = 8.6 Hz, 1H), 5.49 (d, J = 7.2 Hz, 1H), 5.29 (t, J = 5.3 Hz, 1H), 5.02 (q, J = 6.9 Hz, 2H), 4.80 (t, J = 5.9 Hz, 2H), 3.58-3.49 (m, 2H), 3.41-3.34 (m, 5H), 3.09 (s, 1H), 2.82 (q, J = 8.3 Hz, 1H), 2.40 (dd, J = 16.7, 4.0 Hz, 1H), 2.37-2.22 (m, 2H), 2.02-1.98 (m, 1H), 1.82 (m, 1H), 1.62-1.52 (m, 6H); **^{13}C-NMR** ($CDCl_3$, 150 MHz) δ 174.4, 168.6, 167.3, 167.0, 158.9, 144.7, 137.9, 132.4, 130.7, 129.7, 128.2, 127.0 (q, J_{CF3} = 276 Hz), 122.9, 110.3, 107.6, 77.6, 77.4, 70.5, 67.6, 46.4, 42.9, 41.2, 35.9, 35.4, 31.7 (q, J_{CF3} = 28.5 Hz), 26.2, 25.4, 24.8, 24.4.

Note, 3-iodooxetane was also used for another trial.

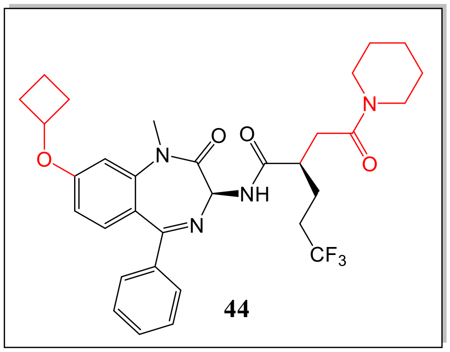

44

(R)-N-((S)-8-cyclobutoxy-1-methyl-2-oxo-5-phenyl-2,3-dihydro-1H-benzo[e][1,4]diazepin-3-yl)-5,5,5-trifluoro-2-(2-oxo-2-(piperidin-1-yl)ethyl)pentanamide

The reaction was performed following the procedure described for **42** using **41** (18 mg, 0.033 mmol) and bromocyclobutane (6.0 µL, 0.066 mmol) The residue was purified by column chromatography on silica gel (MeOH/DCM, 2:98 to 5:90) to afford **44** (33 mg, 61%) as a white solid. Note, epimerization at carbon 3 occurred under the reaction conditions. The two distereomers were separated by HPLC using Method B. The configurations at carbon 3 have not been assigned. NMRs were written based on retention times. **44-Diastereomer A**, HPLC purity via Method B: ≥ 95%, t_R = 26.2 min. HRMS (ESI): m/z $[M + Na]^+$ calculated for $C_{32}H_{37}F_3N_4O_4Na$: 621.2658; found: 621.2655. **^{1}H-NMR** ($CDCl_3$, 600 MHz) δ 7.63 (d, J = 6.5 Hz, 1H), 7.59 (d, J = 7.6 Hz, 2H), 7.46 (t, J = 7.1 Hz, 1H), 7.38 (t, J = 7.4 Hz, 2H), 7.20 (d, J = 9.0 Hz, 1H), 6.74 (s, 1H), 6.64 (d, J = 8.6 Hz, 1H), 5.49 (d, J = 7.2 Hz, 1H), 4.70 (q, J = 7.1 Hz, 1H), 3.58-3.53 (m, 2H), 3.42 (m, 5H), 3.07 (s, 1H), 2.86 (q, J = 8.1 Hz, 1H), 2.49-2.41 (m, 3H), 2.37-2.19 (m, 4H), 2.00-1.90 (m, 2H), 1.83-1.72 (m, 2H), 1.63-1.54 (m, 6H); **^{13}C-NMR** ($CDCl_3$, 150 MHz) δ 174.1, 168.7, 167.7, 167.3, 160.3, 144.7, 137.9, 132.2, 130.7, 129.9, 128.2, 127.0 (q, J_{CF3} = 274.5 Hz), 121.7, 111.4, 107.6, 72.0, 67.9, 46.5, 43.0, 41.6, 35.9, 35.5, 31.5 (q, J_{CF3} = 28.5 Hz), 30.5, 30.4, 26.3, 25.5, 24.9, 24.4, 13.3. **44-Diastereomer B**, HPLC purity via Method B: ≥ 95%, t_R = 26.8 min. HRMS (ESI): m/z $[M + Na]^+$ calculated for $C_{32}H_{37}F_3N_4O_4Na$: 621.2668; found: 621.2668. **^{1}H-**

NMR ($CDCl_3$, 600 MHz) δ 7.94 (s, 1H), 7.58 (d, J = 7.2 Hz, 2H), 7.45 (t, J = 7.2 Hz, 1H), 7.38 (t, J = 7.6 Hz, 2H), 7.22 (t, J = 9.3 Hz, 1H), 6.70 (s, 1H), 6.64 (dd, J = 8.6, 2.1 Hz, 1H), 5.50 (d, J = 7.6 Hz, 1H), 4.73-4.68 (m, 1H), 3.58-3.49 (m, 2H), 3.39 (m, 5H), 3.08 (s, 1H), 2.83 (q, J = 8.3 Hz, 1H), 2.49 (t, J = 7.4 Hz, 2H), 2.41-2.18 (m, 5H), 2.03-1.90 (m, 2H), 1.84-1.72 (m, 2H), 1.61-1.51 (m, 6H); **^{13}C-NMR** ($CDCl_3$, 150 MHz) δ 174.4, 168.7, 167.4, 160.1, 144.5, 138.0, 132.1, 130.6, 129.8, 128.2, 127.0 (q, J_{CF3} = 276 Hz), 121.8, 111.4, 107.5, 71.9, 67.6, 46.4, 42.9, 41.2, 35.9, 35.4, 31.7 (q, J_{CF3} = 28.5 Hz), 30.5, 30.4, 26.2, 25.4, 24.8, 24.4, 13.3.

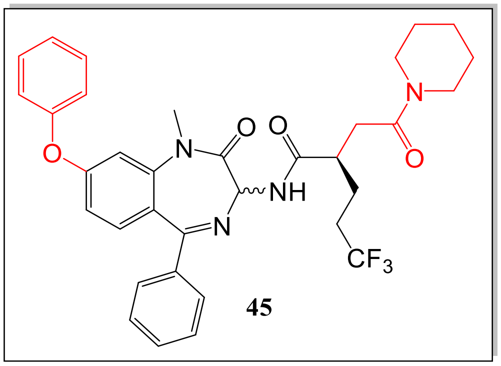

45

(2R)-5,5,5-trifluoro-N-(1-methyl-2-oxo-8-phenoxy-5-phenyl-2,3-dihydro-1H-benzo[e][1,4]diazepin-3-yl)-2-(2-oxo-2-(piperidin-1-yl)ethyl)pentanamide

To solution of **41** (15 mg, 0.028 mmol) in DCM (5 mL) was added phenylboronic acid (10 mg, 0.083 mmol), $Cu(OAc)_2$ (10 mg, 0.055 mmol) and pyridine (9 µl, 0.11 mmol). The mixture was stirred at room temperature and maintained to 40 °C for overnight. After completion, the mixture was diluted with water (20 mL). The aqueous layer was washed with EtOAc (2 × 10 mL), and the combined organic extracts were washed with brine, dried over anhydrous Na_2SO_4, filtered, and concentrated. The residue was purified by column chromatography on silica gel (MeOH/DCM, 5:95 to 10:90) to afford **45** (10 mg, 57%) as a white solid. Note, epimerization at carbon 3 occurred under the reaction conditions. The two diastereomers were separated by HPLC using Method B.

The configurations at carbon 3 have not been assigned. NMRs were written based on retention times. **45-Diastereomer A**, HPLC purity via Method B: 95%, t_R = 27.3 min. HRMS (ESI): m/z $[M + H]^+$ calculated for $C_{34}H_{35}F_3N_4O_4H$: 621.2683; found: 621.2689. **^{1}H-NMR** ($CDCl_3$, 600 MHz) δ 8.19 (d, J = 7.4 Hz, 1H), 7.59 (d, J = 7.4 Hz, 2H), 7.53 (t, J = 7.5 Hz, 1H), 7.47-7.41 (m, 4H), 7.28 (d, J = 7.4 Hz, 1H), 7.25 (d, J = 5.9 Hz, 1H), 7.13 (d, J = 7.7 Hz, 2H), 6.94 (d, J = 2.2 Hz, 1H), 6.80 (dd, J = 8.8, 2.2 Hz, 1H), 5.58 (d, J = 7.4 Hz, 1H), 3.65-3.61 (m, 1H), 3.53-3.49 (m, 1H), 3.47-3.40 (m, 5H), 3.10-3.07 (m, 1H), 2.92 (dd, J = 16.2, 9.0 Hz, 1H), 2.47 (dd, J = 16.4, 4.6 Hz, 1H), 2.33-2.25 (m, 2H), 1.97-1.93 (m, 1H), 1.83-1.78 (m, 1H), 1.68-1.55(m, 6H) ; **^{13}C-NMR** ($CDCl_3$, 150 MHz) δ 174.6, 169.2, 169.1, 166.6, 161.9, 154.5, 145.1, 136.3, 133.3, 131.8, 130.4, 130.3, 128.4, 126.9 (q, J_{CF3} = 274.5 Hz), 125.4, 122.2, 120.4, 113.7, 109.9, 67.0, 46.6, 43.2, 41.4, 35.8, 35.7, 31.5 (q, J_{CF3} = 28.5 Hz), 26.2, 25.4, 24.8, 24.3. **45-Diastereomer B**, HPLC purity via Method B: 95%, t_R = 27.9 min. HRMS (ESI): m/z $[M + H]^+$ calculated for $C_{34}H_{35}F_3N_4O_4H$: 621.2683; found: 621.2683. **^{1}H-NMR** ($CDCl_3$, 600 MHz) δ 8.12 (d, J = 6.7 Hz, 1H), 7.58 (d, J = 7.4 Hz, 2H), 7.50 (t, J = 7.4 Hz, 1H), 7.43 (m, 4H), 7.28 (d, J = 8.8 Hz, H), 7.25 (d, J = 7.6 Hz, 1H), 7.13 (d, J = 7.7 Hz, 2H), 6.92 (d, J = 2.2 Hz, 1H), 6.80 (dd, J = 8.8, 2.2 Hz, 1H), 5.57 (d, J = 7.6 Hz, 1H), 3.60-3.56 (m, 1H), 3.50-3.46 (m, 1H), 3.44-3.38 (m, 5H), 3.07 (m, 1H), 2.88 (dd, J = 16.4, 9.1 Hz, 1H), 2.44 (dd, J = 16.4, 4.5 Hz, 1H), 2.37-2.23 (m, 2H), 2.05-1.99 (m, 1H), 1.86-1.80 (m, 1H), 1.66-1.52 (m, 6H); **^{13}C-NMR** ($CDCl_3$, 150 MHz) δ 174.7, 169.0, 168.4, 167.0, 161.3, 154.7, 144.8, 136.8, 132.9, 131.4, 130.3, 130.1, 128.40, 126.9 (q, J_{CF3} = 276.0 Hz), 125.2, 122.7, 120.3, 113.7, 109.9, 67.1, 46.6, 43.2, 41.3, 35.7, 35.5, 31.50 (q, J_{CF3} = 28.5 Hz), 26.2, 25.4, 24.8, 24.3.

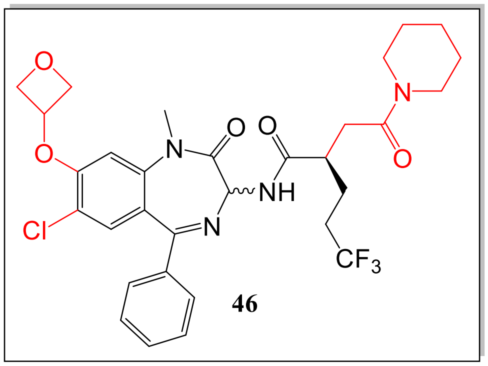

46

(2R)-N-(7-chloro-1-methyl-8-(oxetan-3-yloxy)-2-oxo-5-phenyl-2,3-dihydro-1H-benzo[e][1,4]diazepin-3-yl)-5,5,5-trifluoro-2-(2-oxo-2-(piperidin-1-yl)ethyl)pentanamide

To solution of **13** (30 mg, 0.08 mmol) in DCM (5 mL) was added **23c** (27 mg, 0.096 mmol), EDC·HCl (23.1 mg, 0.121 mmol), HOBt hydrate (18.5 mg, 0.121 mmol) and triethylamine (16.8 µl, 0.121 mmol). The reaction was maintained at room temperature for overnight. After completion, the mixture was diluted with water (20 mL). The aqueous layer was washed with EtOAc (2 × 10 mL), and the combined organic extracts were washed with brine, dried over anhydrous Na_2SO_4, filtered, and concentrated. The residue was purified by column chromatography on silica gel (MeOH/DCM, 5:95 to 10:90) to afford **46** (47 mg, 91%) as a white solid. Note, epimerization at carbon 3 occurred under the reaction conditions. The two diastereomers were separated by HPLC using Method B. The configurations at carbon 3 have not been assigned. NMRs were written based on retention times. **46-Diastereomer A**, HPLC purity via Method B: 95%, t_R = 25.6 min. HRMS (ESI): m/z $[M + Na]^+$ calculated for $C_{31}H_{34}ClF_3N_4O_5Na$: 657.2062; found: 657.2050. **^{1}H-NMR** ($CDCl_3$, 600 MHz) δ 7.59 (d, J = 7.4 Hz, 2H), 7.53 (d, J = 7.2 Hz, 1H), 7.49 (t, J = 7.4 Hz, 1H), 7.41 (t, J = 7.7 Hz, 2H), 7.35 (s, 1H), 6.37 (s, 1H), 5.46 (d, J = 7.2 Hz, 1H), 5.35-5.31 (m, 1H), 5.06-5.04 (m, 2H), 4.89 (ddd, J = 28.8, 7.3, 5.3 Hz, 2H), 3.60-3.52 (m, 2H), 3.41 (m, 5H), 3.09-3.06 (m, 1H), 2.83 (q, J = 8.3 Hz, 1H), 2.44-2.26 (m, 3H), 1.99-1.94 (m, 1H), 1.83-1.78 (m, 1H), 1.66-1.51 (m, 6H); **^{13}C-NMR** ($CDCl_3$, 150 MHz) δ 174.2, 168.6, 167.1, 165.9, 154.5, 143.0, 137.2,

131.9, 131.0, 129.7, 128.4, 127.0 (q, J_{CF3} = 279 Hz), 123.3, 119.3, 105.5, 77.46, 77.15, 71.5, 68.3, 46.4, 42.9, 41.5, 35.9, 35.6, 31.5 (q, J_{CF3} = 28.3 Hz), 26.3, 25.5, 24.9, 24.4. **46-Diastereomer B**, HPLC purity via Method B: 95%, t_R = 26.1 min. HRMS (ESI): m/z $[M + Na]^+$ calculated for $C_{31}H_{34}ClF_3N_4O_5Na$: 657.2062; found: 657.2056. **^{1}H-NMR** ($CDCl_3$, 600 MHz) δ 7.89 (d, J = 7.4 Hz, 1H), 7.58-7.56 (m, 2H), 7.49 (t, J = 7.4 Hz, 1H), 7.41 (t, J = 7.7 Hz, 2H), 7.38 (s, 1H), 6.34 (s, 1H), 5.47 (d, J = 7.6 Hz, 1H), 5.35-5.31 (m, 1H), 5.06-5.03 (m, 2H), 4.89 (ddd, J = 39.3, 7.3, 5.3 Hz, 2H), 3.59-3.47 (m, 2H), 3.41-3.34 (m, 5H), 3.08 (m, 1H), 2.83 (dd, J = 16.4, 9.0 Hz, 1H), 2.41-2.37 (m, 1H), 2.36-2.21 (m, 2H), 2.04-1.97 (m, 1H), 1.84-1.79 (m, 1H), 1.64-1.48 (m, 6H); **^{13}C-NMR** ($CDCl_3$, 150 MHz) δ 174.5, 168.7, 167.1, 165.8, 154.4, 142.8, 137.3, 132.0, 131.0, 129.6, 128.4, 127.0 (q, J_{CF3} = 275.4 Hz), 123.4, 119.4, 105.4, 77.4, 77.1, 71.4, 67.7, 46.4, 42.9, 41.1, 35.9, 35.4, 31.6 (q, J_{CF3} = 28.8 Hz), 26.2, 25.4, 24.8, 24.4.

***Cryptosporidium* infection of HCT-8 cells and dose-response testing**

Human ileocecal adenocarcinoma (HCT-8, ATCC CCL244) cells were maintained in T-75 tissue culture flasks with RPMI 1640 medium supplemented with 10% heat-inactivated fetal bovine serum, L-glutamine, 100 IU penicillin, and 100 mg/mL streptomycin. For *C. parvum* infection assays, HCT-8 cells were plated (5 µL/well) into 1536-well assay plates at a density of 3,000 cells/well using a MultiFlo FX Multi-Mode Dispenser (Biotek) and allowed to grow for 24 hours. Just prior to infection, experimental compounds are serially diluted 1:3 in 11-points and then acoustically transferred with an Echo 555 (Labcyte) into triplicate 1536-well plates containing HCT-8 cells, with a top concentration of 25 µM. *Cryptosporidium parvum* oocysts (Iowa strain, Bunch Grass Farm, Deary, ID) are excysted in bile salts and prepared for inoculation. The oocysts

are diluted with assay medium to 3,125 oocysts/well and dispensed (3 µL/well, 8 µL/well final volume) onto compound-treated HCT-8 cells within 2 hours of compound transfer. Plates are spun at 150×g for 3 min in a centrifuge to aide in sporozoite attachment. Infected cells are then allowed to grow for 48 h in a humidified incubator at 37 °C.

High-Content Imaging of *Cryptosporidium* proliferation and data analysis

After 48 h incubation, infected cells are fixed with 4% paraformaldehyde and stained with fluorescein isothiocyanate (FITC)-conjugated *Vicia villosa* lectin and 4',6-diamidino-2-phenylindole (DAPI) to visualize host cell nuclei. After sealing the plates with adhesive foil, the cells are imaged with a CellInsight CX5 High Content Screening Platform (Thermo) with a 10× objective. Two channels were used: 384/440 nm for DAPI-stained nuclei, and 485/521 nm for FITC-lectin-labeled *Cryptosporidium* parasites. One microscopic field (802,511.39 μm^2) per well is captured. The software identifies primary objects (HCT-8 host cells) and spots within allowed distances to the nuclei (*Cryptosporidium*). Both cytotoxicity against HCT-8 cells (number of nuclei relative to DMSO-treated controls) and *Cryptosporidium* inhibition (spot counts relative to DMSO-treated controls) are assessed. Host cell and *Cryptosporidium* counts are analyzed in Genedata Screener (v13.0-Standard), with the *Cryptosporidium* Spot Count and HCT-8 cell Selected Object Count are normalized to neutral controls minus inhibitors (floxuridine for Spot Count, and puromycin for Selected Object Count; neutral control is an equivalent volume of dimethyl sulfoxide). Dose-response curves are fit with Genedata Analyzer using the Smart Fit function and EC_{50}, EC_{90} and CC_{50} (cytotoxicity) values are determined.

Experimental procedure for Cp growth inhibition assay with shortened sporozoite exposure time

Isolation of sporozoites and growth of *C. parvum* in cell culture was performed as described previously.[1, 2] Briefly, purified *C. parvum* IOWA isolate oocysts were washed with phosphate-buffered saline (PBS, pH 7.4) to remove the storage buffer (2.5% w/v aqueous potassium dichromate) and resuspended in RPMI 1640 media supplemented with 0.75% sodium taurocholate and incubated for 30 min at 37 °C. After excystation the *C. parvum* oocysts and sporozoite mixture was filtered through a 3.0 µM filter using a syringe and centrifuged, washed and counted. Isolated sporozoites were incubated with compound for 45 min, then washed several times to remove the compound before plating on cell monolayers.

Approximately 1×10^5 sporozoites were dispensed per well in 24 well plates containing confluent HCT-8 cells. Host cells inoculated with the sporozoite were incubated for 3 h and subsequently washed with PBS to remove free parasites (those that had not invaded host cells). PBS was replaced with fresh RPMI 1640 with 10% FBS with or without test compounds and the cultures incubated for 48 h. In addition, RPMI 1640 with 10% FBS supplemented with DMSO (same concentration used with test compounds) was evaluated to determine the effect of inhibition or toxicity on cultures. Culture wells were washed with PBS, and then PBS replaced by Bouin's solution.

After fixation of cultures, the Bouin's solution was removed and decolorized with 70% ethanol, followed by 5 washes with PBS. Culture wells were labelled with an anti-*C. parvum* fluorescein-labeled monoclonal antibody (C3C3-FITC) and parasites enumerated via quantitative fluorescent microscopy at 400X magnification using an Olympus 1X73 microscope. 24 sequential, non-overlapping fields per well were captured as digital images which represented a subsample of the entire well. The parasites were quantified using OpenCFU

(https://sourceforge.net/projects/opencfu/ software (Geissmann, 2013) based on the size of the fluorescing parasites and the fluorescent signal. Data were obtained from four well replicates and each experiment was repeated at least twice. Dose–effect curves and the median effective concentration (EC_{50}) of each compound was determined using CompuSyn (ComboSyn, Inc., Paramus, NJ, ver. 2.1 for Microsoft Windows) computer software. The EC_{50} was defined as the concentration required for reducing the number of parasites by 50% compared with the untreated control.

Experimental procedure and data for Notch assay

HEK293T/17 cells were plated (20,000 cells/well) in a white clear bottom 96 well tissue culture plate one day prior to transfection. Cells were transfected with an expression vector for NotchΔE, a reporter vector containing a Notch-responsive firefly luciferase gene (CSL (CBF1/RBP-JK) and a vector for the constitutive expression of Renilla luciferase (transfection control) using MirusBio Transit-LTI transfection reagent using Notch Pathway Reporter Kit (BPS BioScience, Kit# 60509) following the manufacture's protocol. 24 h post-transfection, media was aspirated and replaced with 50 μL DMEM containing compound or DMSO (vehicle control). The cells were incubated at 37C for 24 h with compound and Notch activity was determined using Dual-GLo Luciferase Assay System (Promega #E2920) according to manufacturer's protocol. Briefly, the 96 well plate was incubated at RT for 30 min to reduce temperature to around 25C (RT). Luciferase reagent (50 μL) was added per well using channel pipettor and the plate was gently agitated in dark for 15 min at which time Firefly luciferase luminescence was measured on BioTek plate reader. Stop & GIO reagent (50 μL) was then added and following another 15 min incubation in dark, Renilla firefly luminescence was measured. To obtain normalized luciferase activity for CSL (CBFI/ RBP-Jk)

reporter, background luminescence was subtracted (background: non-transfected control cells which were otherwise treated identically to other wells [DMSO in media]) and the ratio of firefly luminescence (CSLreporter) to Renilla luminescence was calculated.

Table S1. Notch inhibitory activity of BMS906024 derivatives.

Compd ID	Notch*		
	Activity (%) at 100 nM	Activity (%) at 1000 nM	N
**BMS906024 (1)**	4.3 (±0.1)	N.D.	3
**36**	121.0 (±17.7)	N.D.	3
**28a**	57.5 (±4.5)	10.6 (±0.8)	3
**28b**	103.3 (±28.9)	14.4 (±4.4)	3
**29**	153.6 (±8.4)	69.2 (±14.3)	3
**30**	75.1 (±5.6)	18.2 (±0.7)	3
**27a**	157.5 (±21.9)	43.1 (±8.6)	3
**35**	179.3 (±49.7)	88.1 (±11.8)	3
**37**	93.8 (±15.1)	99.3 (±11.1)	3
**31**	50.8 (±8.6)	8.9 (±1.9)	3
**32**	35.7 (±8.3)	8.1 (±2.8)	3
**33**	91.5 (±14.0)	13.0 (±3.8)	3
**38b**	117.3 (±17.8)	112.4 (±10.4)	3
**38a**	81.1 (±3.1)	79.4 (±3.7)	3
**39**	83.4 (±9.7)	93.5 (±23.7)	3
**28c**	44.5 (±1.5)	5.3 (±0.96)	3
**34**	22.2 (±1.4)	5.2 (±0.2)	3
**44a**	59.4 (±6.7)	10.6 (±0.7)	3
**44b**	115.0 (±8.7)	133.0 (±21.1)	3
**43a**	24.5 (±1.0)	6.7 (±0.8)	3
**43b**	80.3 (±5.5)	65.4 (±3.4)	3
**42a**	63.7 (±2.6)	13.7 (±1.2)	3
**42b**	100.8 (±1.6)	98.7 (±5.7)	3
**45a**	19.4 (±1.6)	5.6 (±0.74)	3
**45b**	85.2 (±2.1)	64.5 (±1.9)	3
**46a**	49.4 (±0.91)	10.4 (±1.4)	3
**46b**	85.1 (±8.1)	88.2 (±8.2)	3

*Notch assays were calculated related to DMSO (100%). N.D.: Not determined

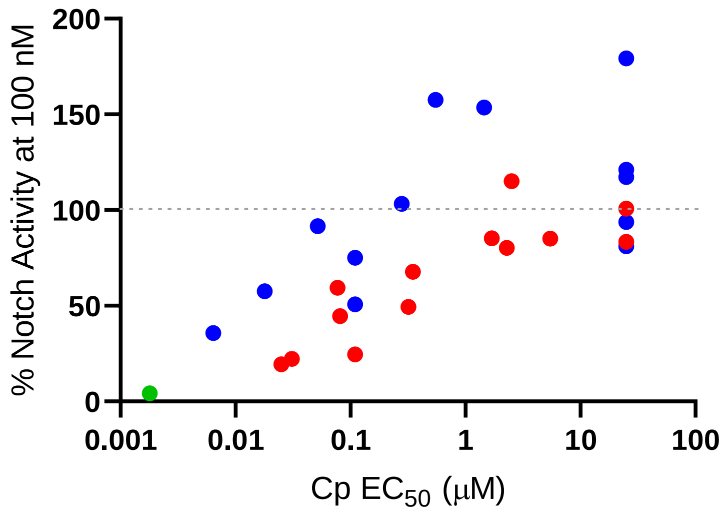

Figure S1. The correlation of *C. parvum* growth (Cp EC_{50}) and Notch inhibitory activities (% Notch activity at 100 nM) for BMS906024 and select derivatives. BMS906024 is shown as a green dot, succinyl portion modifications are shown as blue dots and benzodiazepine portion modifications are shown as red dots.

References

1. Keelaghan AP, Charania R, Mead JR. The Effect of Short-Chain Fatty Acids on Growth of Cryptosporidium parvum In Vitro. *Microorganisms.* 2022;10(9).
2. Geissmann Q. OpenCFU, a new free and open-source software to count cell colonies and other circular objects. *PLoS One.* 2013;8(2): e54072.
